# An annotated checklist and integrative biodiversity discovery of barnacles (Crustacea, Cirripedia) from the Moluccas, East Indonesia

**DOI:** 10.3897/zookeys.945.39044

**Published:** 2020-07-03

**Authors:** Pipit Pitriana, Luis Valente, Thomas von Rintelen, Diana S. Jones, Romanus E. Prabowo, Kristina von Rintelen

**Affiliations:** 1 Museum für Naturkunde- Leibniz Institute for Evolution and Biodiversity Science, Invalidenstrasse 43, 10115 Berlin, Germany Leibniz Institute for Evolution and Biodiversity Science Berlin Germany; 2 Research Centre for Deep-sea, Indonesian Institute of Science (LIPI), Jl. Y. Syaranamual, Poka, Tlk. Ambon, Kota Ambon, Maluku, Indonesia esearch Centre for Deep-sea, Indonesian Institute of Science Kota Ambon Indonesia; 3 Naturalis Biodiversity Center, Understanding Evolution Group, Postbus 9517, 2300 RA Leiden, the Netherlands Freie Universität Berlin Berlin Germany; 4 The Western Australian Museum, 49 Kew Street, Welshpool WA 6106, Locked Bag 49, Welshpool DC WA 6986, Australia Naturalis Biodiversity Center Leiden Netherlands; 5 Faculty of Biology, Universitas Jenderal Soedirman, Purwokerto, 53122, Indonesia The Western Australian Museum Welshpool Australia; 6 Institute of Geological Sciences, Freie Universität Berlin, Malteserstrasse 74-100 Building C and D, 12249 Berlin, Germany Universitas Jenderal Soedirman Purwokerto Indonesia

**Keywords:** Acorn barnacle, coral triangle, Indonesian biodiversity, new records, stalked barnacles, taxonomy

## Abstract

To contribute to the taxonomic knowledge of barnacles in this understudied area, the first checklist of barnacles from the Moluccas is presented, including additional information on morphology, distribution, and substrate as well as molecular data. The species of barnacles from the Moluccas have been determined using morphological analysis and DNA sequences. During 19 field trips conducted between January 2016 and September 2017, 1,513 specimens of 24 species of intertidal and one species of deep-sea barnacles were collected from 51 localities from the islands. Morphological and molecular analysis of the collected material detected members of three families of stalked barnacles and four families of acorn barnacles. In addition to sampling in the field, we also surveyed the literature on barnacles from the Moluccas. In total, our checklist comprises 97 species from the Moluccas including 23 new records, two of them yet to be described species. Results suggest that the Moluccas have a much higher diversity of barnacles than previously known, for example, from the reports of *Challenger* and *Siboga* expeditions. For further work, routine application of molecular systematics could aid the detection of cryptic species, while increased sampling of more islands and a taxonomic revision of several groups would likely lead to an even higher number of species than currently known.

## Introduction

Barnacles (Crustacea, Cirripedia) are an ancient, species-rich and abundant group of crustaceans with about 1,400 extant species (Newman and Abbott 1980). They have a worldwide distribution in tropical and temperate marine environments and at different depths and are adjusted to various lifestyles, from parasites of decapod crustaceans to free-living groups. Most cirripeds usually have two free-swimming planktonic larval stages consisting of distinctive nauplii and a unique non-feeding cyprid (Darwin 1852, 1854; Pochai et al. 2017). In the most abundant group, the Thoracica, adult specimens are permanently attached to various types of substrates, other living organisms (e.g., mangroves, corals, molluscs, other barnacles, sponges), rocks, and man-made materials such as cargo ships and concrete walls (Newman and Abbott 1980; Power et al. 2010). The Thoracica comprise the orders Cyprilepadiformes, Ibliformes, Lepadiformes, Scalpelliformes, and Sessilia (Buckeridge and Newman 2006).

**Figure 1.**
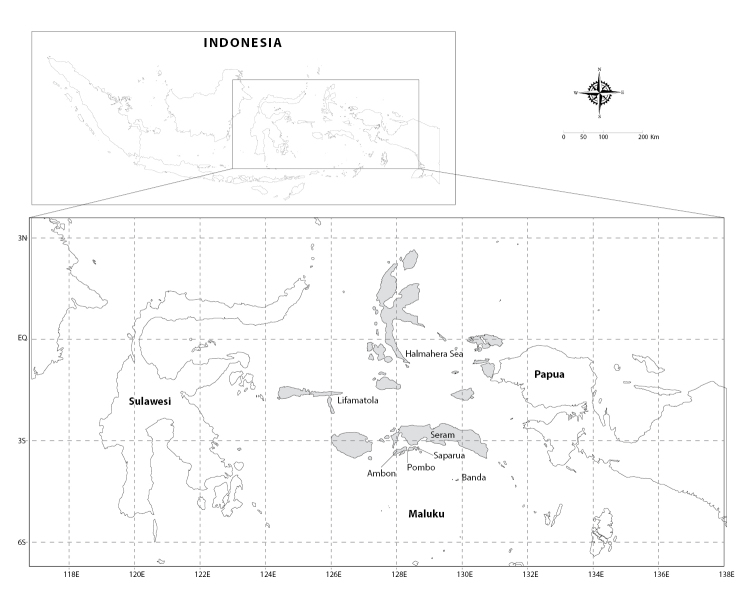
Map of the Moluccan Islands (Maluku in grey) in eastern Indonesia. Map modified from Shorthouse (2010).

The Indonesian Moluccas (or Spice Islands; Fig. 1) are part of the Coral Triangle, one of the most complex biogeographical and oceanographic areas on Earth. Although part of the global epicentre of marine biodiversity, knowledge of the barnacle fauna of the Moluccas is relatively sparse. The exploration of the natural history of the Moluccas dates back to the 17^th^ century, starting with Georg Everhard Rumphius, and later, for example, Alfred Russel Wallace (Strack 1993; Lamoureux 1990). In the 19^th^ and 20^th^ centuries, there were approximately fifty scientific expeditions passing through or specifically targeting the area, such as the British *Challenger* (1872–1876), the Dutch *Siboga* (1899–1900) and the *Snellius* (1929–1930) expeditions (Lamoureux 1990). The most recent being the French *Karubar* expedition in 1991 (Crosnier et al. 1997).

Rumphius provided the first record of a barnacle (the stalked *Mitella* Oken, 1815 (= *Capitulum* Gray, 1825) found on a rock near the beach at Ambon Island) in his posthumously published ‘Amboinsche Rariteitkamer’ (Rumphius 1705). Indonesian and Moluccan barnacles were also studied by Darwin (1854), who assigned them to one of four geographical ‘provinces’, the third being the East Indian Archipelago. Moluccan barnacles have not been studied since Buckeridge (1994) examined some material from the *Karubar* expedition.

To contribute to the taxonomic knowledge of this understudied area, we herein present the first checklist of barnacles from the Moluccas, including additional information on morphology and molecular data, as well as distribution and substrate.

## Material and methods

### Sampling

Specimens examined in this study were collected by the first author during 19 field trips between January 2016 and September 2017 to the intertidal zones of the Moluccan islands of Ambon, Saparua, Seram, Pombo, and Banda Neira (Fig. 1, Table 1, Suppl. material 1: Table S1). Deep-water barnacles (Table 1) from the Lifamatola Passage (250 m) and Halmahera Sea (250 m) were provided by Nurul Fitriya. Additional material used for the molecular analyses was collected from the island of Sulawesi in September to October 2017 (Suppl. material 1: Table S1). In total, 159 lots containing 1,513 specimens were collected from 51 Moluccan localities.

Barnacle specimens firmly attached to hard substrate (rocks, stone, concrete) were sampled using a chisel and hammer whereas those attached to softer substrate using a craft knife. Specimens were fixed and stored in 96% ethanol and transferred into 75% ethanol for long-term preservation.

**Table 1.** Annotated checklist tabulation of barnacle species from the Moluccas, Eastern Indonesia.

ORDER	Family	Genus / Species		Locality	Substrate	References
Suborder	Subfamily	No.	Name			
LEPADIFORMES Heteralepadomorpha	Heteralepadidae	1	*Heteralepas japonica* (Aurivillius, 1892)	Lifamatola Sea and Halmahera Sea	Deep-water cable to mooring	This study
		2	*Heteralepas ovalis* (Hoek, 1907)	West from Kei Islands		Hoek (1913)
		3	*Heteralepas tenuis* (Hoek, 1907)	South of Seram		Hoek (1913)
Lepadomorpha	Oxynaspididae	4	*Oxynaspis connectens* Broch, 1931	Kei Islands		Jones and Hosie (2016)
	Poecilasmatidae	5	*Glyptelasma carinatum* (Hoek, 1883)	Seram Sea		Hoek (1913)
		6	*Megalasma striatum* (Hoek, 1883)	East of Kei Islands		Hoek (1913)
		7	*Octolasmis orthogonia* Darwin, 1852	Tual anchorage		Hoek (1913)
		8	*Octolasmis weberi* (Hoek, 1907)	Kei Islands, Banda Sea		Hoek (1913), Jones and Hosie (2016)
		9	*Poecilasma kaempferi* Darwin, 1852	Banda Sea		Jones and Hosie (2016)
		10	*Temnaspis fissum* (Darwin, 1852)	Ternate anchorage		Hoek (1913)
	Lepadidae	11	*Conchoderma virgatum* Spengler, 1789	Banda Sea		Jones et al. (2001)
		12	*Dosima fascicularis* (Ellis & Solander, 1786)	Ambon	Floating in water at the beach	This study
		13	*Lepas anserifera* Linnaeus, 1767	Ambon, Saparua, Seram, Pombo	Mangrove, stone ship chart and ship wall, port pole, shell of *Megabalanus zebra*	This study
		14	*Lepas pectinata* Spengler, 1793	Banda Sea		Jones and Hosie (2016)
SCALPELLIFORMES	Calanticidae	15	*Calantica pollicipedoides* (Hoek, 1907)	East of Kei Islands		Hoek (1913)
		16	*Euscalpellum rostratum* (Darwin, 1852)	Kei Islands		Jones and Hosie (2016)
	Pollicipedidae	17	*Capitulum mitella* (Linnaeus, 1758)	Ambon, Saparua	Rocks, stone, wall of fortress, port pole and concrete wall	Rumphius (1705), this study
	Scalpellidae	18	*Compressoscalpel-lum inflatum* (Hoek, 1907)	West of Aru Island		Hoek (1913)
	Scalpellinae					
		19	*Scalpellum fissum* Hoek, 1913	West of Halmahera		Hoek (1913)
		20	*Scalpellum stearnsi* Pilsbry, 1890	Near Kei Islands		Hoek (1913)
	Arcoscalpellinae	21	*Amigdoscalpellum vitreum* (Hoek, 1883)	South of Seram, South of Ambon		Hoek (1913)
		22	*Anguloscalpellum pedunculatum* (Hoek, 1883)	Kei Islands		Hoek (1913)
		23	*Arcoscalpellum cilliatum* (Hoek, 1907)	South of Ambon		Hoek (1913)
		24	*Arcoscalpellum discolor* (Hoek, 1907)	Banda Sea		Hoek (1913)
		25	*Arcoscalpellum sculptum* (Hoek, 1907)	Banda Sea		Hoek (1913)
		26	*Arcoscalpellum sociabile* (Annandale, 1905)	Banda Sea		Jones et al. (2001)
SCALPELLIFORMES	Arcoscalpellinae	27	*Planoscalpellum hexagonum* (Hoek, 1907)	Banda Sea		Hoek (1913)
		28	*Teloscalpellum imbricatum* (Hoek, 1907)	Near Kei Islands		Hoek (1913)
		29	*Trianguloscalpellum balanoides* (Hoek, 1883)	Banda Sea		Hoek (1913), Shalaeva and Boxshall (2014)
		30	*Trianguloscalpellum diota* (Hoek, 1907)	Near Kei Islands		Hoek (1913)
		31	*Trianguloscalpellum hamulus* (Hoek, 1907)	Kei Islands		Hoek (1913)
		32	*Trianguloscalpellum hirsutum* (Hoek, 1883)	Moluccan Sea		Hoek (1913), Shalaeva and Boxshall (2014)
		33	*Trianguloscalpellum indicum* (Hoek, 1883)	Banda Sea		Hoek (1913), Shalaeva and Boxshall (2014)
		34	*Trianguloscalpellum moluccanum* (Hoek, 1883)	Banda Sea; West of Aru Island		Hoek (1913), Shalaeva and Boxshall (2014)
		35	*Trianguloscalpellum sessile* (Hoek, 1907)	Seram Sea		Hoek (1913)
		36	*Verum candidum* (Hoek, 1907)	Near Kei Islands		Hoek (1913), Jones and Hosie (2016)
	Meroscalpellinae	37	*Annandaleum japonicum* (Hoek, 1883)	Aru Island		Hoek (1913)
		38	*Annandaleum laccadivicum* (Annandale, 1906)	Kei Islands		Hoek (1913)
SESSILIA Verrucomorpha	Verrucidae	39	*Altiverruca navicula* (Hoek, 1913)	Between Seram and New Guinea, Kei Islands, Tanimbar Island		Hoek (1913), Buckeridge (1994)
		40	*Brochiverruca dens* (Broch, 1932)	Tanimbar Island		Buckeridge (1994)
		41	*Cristallinaverruca cristallina* (Gruvel, 1907)	Banda Sea		Jones and Hosie (2016)
		42	*Metaverruca recta* (Aurivillius, 1898)	Kei Islands		Hoek (1913)
		43	*Newmaniverruca albatrossiana* (Pilsbry, 1912)	East of Kei Islands, Tanimbar Island		Hoek (1913), Buckeridge (1994)
		44	*Rostratoverruca intexta* (Pilsbry, 1912)	Kei Islands, Tanimbar Island		Hoek (1913), Buckeridge (1994)
		45	*Rostratoverruca kruegeri* (Broch, 1922)	Kei Islands, Tanimbar Island		Jones et al. (2001), Buckeridge (1994)
		46	*Verruca capsula* Hoek, 1913	Between Seram and New Guinea		Hoek (1913)
Balanomorpha	Pachylasmatidae	47	*Hexelasma arafurae* Hoek, 1913	Kei Islands,Arafura Sea		Hoek (1913), Jones and Hosie (2016)
	Hexelasmatinae					
		48	*Hexelasma velutinum* Hoek, 1913	Kei Islands		Jones et al. (2001)
	Pachylasmatinae	49	*Pachylasma integrirostrum* Broch, 1931	Ambon		Jones et al. (2001)
Balanomorpha	Pachylasmatinae	50	*Pseudoctomeris sulcata* (Nilsson-Cantell, 1932)	Ambon	Rocks, shell of *Tetraclita squamosa*	This study
	Chthamalidae	51	*Hexechamaesipho pilsbryi* (Hiro, 1936)	Ambon	Rocks	This study
	Notochthamalinae					
		52	*Nesochthamalus intertextus* (Darwin, 1854)	Ambon	Stone	This study
	Euraphiinae	53	*Europhia hembeli* Conrad, 1837	Ambon	Rocks	This study
		54	*Microeuraphia withersi* (Pilsbry, 1916)	Kei Islands		Jones and Hosie (2016)
		55	*Microeuraphia* sp.	Seram Island	Stone, concrete wall at port	This study
	Chthamalinae	56	*Chthamalus malayensis* Pilsbry, 1916	Arafura Sea		Jones et al. (2001)
		57	*Chthamalus moro* Pilsbry, 1916	Ambon, Saparua, Seram, Pombo	Mangrove, stone, port pole, mollusc shell, shells of *Tetraclita squamosa*, *Tesseropora rosea*, and *Capitulum mitella*	This study
	Tetraclitidae	58	Tetraclitella (Eotetraclitella) costata (Darwin, 1854)	Banda		Hoek (1913)
	Tetraclitellinae	59	*Tetraclitella divisa* (Nilsson-Cantell, 1921)	Ambon	Concrete wall at port	This study
		60	*Tetraclitella karandei* Ross, 1971	Ambon	Stone, shells of *Capitulum mitella* and *Euraphia hembeli*	This study
	Tetraclitinae	61	*Tesseropora rosea* (Krauss, 1848)	Ambon, Saparua	Stone, mollusc shell	This study
		62	*Tetraclita kuroshioensis* Chan, Tsang & Chu, 2007	Ambon, Saparua	Rocks, concrete wall at port	This study
		63	*Tetraclita squamosa* (Bruguière, 1789)	Ambon, Saparua	Stone, rocks, concrete bridge and wall at port, shipyard.	This study
	Newmanellinae	64	*Yamaguchiella coerulescens* (Spengler, 1790)	Banda, Kei Islands, Ambon, Saparua	Stone	Hoek (1913), Jones and Hosie (2016), This study
		65	*Neonrosella vitiata* (Darwin, 1854)	Ambon, Banda Neira, Saparua	Port pole, reef, stone	This study
		66	*Newmanella spinosus* Chan & Cheang, 2016	Ambon	Stone, reef surface	This study
	Archaeobalanidae	67	*Armatobalanus allium* (Darwin, 1854)	Banda Sea		Jones and Hosie (2016)
	Archaeobalaninae	68	*Armatobalanus cepa* (Darwin, 1854)	Aru Island		Jones and Hosie (2016)
		69	*Armatobalanus quadrivittatus* (Darwin, 1854)	Banda Sea		Jones and Hosie (2016)
		70	*Conopea dentifer* (Broch, 1922)	Kei Islands		Jones et al. (2001)
		71	*Conopea navicula* (Darwin, 1854)	Near Damar Island (South of Halmahera)		Hoek (1913)
Balanomorpha	Archaeobalanidae	72	*Membranobalanus cuneiformis* (Hiro, 1936)	Arafura Sea		Jones and Hosie (2016)
	Archaeobalaninae	73	*Solidobalanus auricoma* (Hoek, 1913)	Banda Sea, Ternate, Kei Islands		Hoek (1913), Jones et al. (2001)
		74	*Solidobalanus socialis* (Hoek, 1883)	Arafura Sea, Ternate, Kei Islands		Hoek (1913)
		75	*Striatobalanus amaryllis* (Darwin, 1854)	Arafura Sea		Jones and Hosie (2016)
		76	*Striatobalanus kruegeri* (Pilsbry, 1916)	Moluccas		Jones and Hosie (2016)
		77	*Striatobalanus tenuis* (Hoek, 1883)	Kei Islands, Arafura Sea		Hoek (1913), Jones and Hosie (2016)
	Bryozobiinae	78	*Multatria terebratus* (Darwin, 1854)	Kei Islands		Hoek (1913), Jones and Hosie (2016)
		79	*Eoatria quinquevittatus* (Hoek, 1913)	Banda Sea, Ambon Island		Jones and Hosie (2016)
	Pyrgomatidae	80	*Cantellius euspinulosum* (Broch, 1931)	Ambon		Jones and Hosie (2016)
	Pyrgomatinae	81	*Cantellius gregarious* (Sowerby, 1823)	Banda Sea		Jones et al. (2001)
		82	*Cantellius pallidus* (Broch, 1931)	Banda Sea		Jones et al. (2001)
		83	*Galkinius indica* (Annandale, 1924)	Kei Islands		Jones and Hosie (2016)
		84	*Hoekia fornix* Ross & Newman, 1995	Moluccas		Jones and Hosie (2016)
		85	*Nobia grandis* Sowerby, 1839	Kei Islands		Jones et al. (2001)
		86	*Pyrgoma kuri* Hoek, 1913	Kei Islands		Hoek (1913)
	Balanidae	87	*Amphibalanus amphitrite* (Darwin, 1854)	Ambon, Saparua	Stone, mollusc shell, capitulum of *Lepas anserifera*	This study
	Amphibalaninae	88	*Amphibalanus reticulatus* (Utinomi, 1967)	Ambon	Stone, concrete wall at port	This study
		89	*Amphibalanus variegatus* (Darwin, 1854)	Ambon, Saparua	Stone, plastic	This study
		90	*Amphibalanus zhujiangensis* (Ren, 1989)	Ambon, Saparua, Seram	Stone, capitulum of *Lepas anserifera*	This study
		91	*Amphibalanus* sp.	Ambon, Seram	Stone, concrete wall at port	This study
	Balaninae	92	*Balanus arcuatus* Hoek, 1913	Banda		Hoek (1913)
		93	*Balanus hystrix* Hoek, 1913	Ambon		Hoek (1913)
		94	*Balanus longirostrum* Hoek, 1913	Bacan		Hoek (1913)
	Megabalaninae	95	*Megabalanus occator* (Darwin, 1854)	Near Obilatu Island	Coral	Kolosváry (1950)
		96	*Megabalanus tintinnabulum* (Linnaeus, 1758)	Ambon, Saparua	Conrete bridge at port, stone, reef surface	This study
		97	*Megabalanus zebra* (Darwin, 1854)	Ambon	Stone, capitulum of *Lepas anserifera*	This study

### Morphological analysis

For detailed morphological analyses, all samples were studied at the Museum für Naturkunde in Berlin (**ZMB**), Germany. All specimens are deposited at the Museum Zoologicum Bogoriense (**MZB**; Suppl. material 1: Table S1), Research Center for Biology, Indonesian Institute of Sciences- **LIPI**, Indonesia. Barnacle species attached to other barnacle species were not separated (except for specimens dissected and measured) but were kept within the same glass container, enabling further morphological studies of different species attached to each other, e.g., with MicroCT scans.

Specimens were studied by the first author. All species were determined based on external shell morphology, including the pattern of the parietes, opercular plates, mouth parts, and arthropodal characters, as described by Darwin (1852, 1854), Hoek (1907, 1913), Southward and Newman (2003), Chan et al. (2007), Pérez-Losada (2008, 2014), Chan et al. (2009a), Tsang et al. (2015), and Chan et al. (2017).

**Figure 2.**
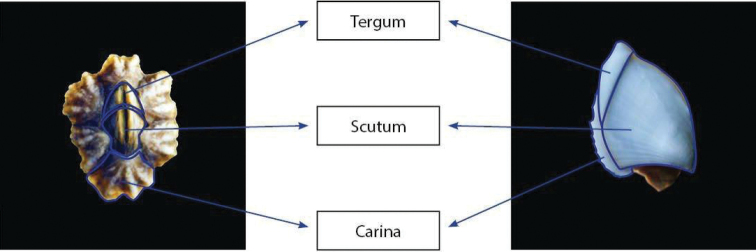
External morphology of barnacles showing the two parts of the operculum (tergum and scutum) and carina (without scale).

Hard body parts (parietes and opercular plates) were separated from soft body parts using a scalpel. Shell plates were separated and cleaned with a bleach solution to remove any organic material, rinsed with fresh water, dried and observed under a stereo microscope (Leica M125) and photographed with a digital camera (Leica Microsystems M205C and Leica Z16 APo-A) (Fig. 2).

The mouthparts (labrum, palps, maxilla, maxillule, and mandible) were dissected using a scalpel, each was mounted on a glass slide and examined under a light microscope (Axioskop 20). The cirri were separated into couples of cirri I–VI and the penis, before being mounted on glass slides. The anatomy of these soft body parts was studied using a light microscope.

All measurements were made using digital callipers (accurate to 0.1 mm; Suppl. material 1: Tables S2–S26) generally following the method described in Beşir and Çınar (2012): basal length of shell, basal width, orifice length, orifice width, and carinal height. For stalked cirripeds, measurement of total height, capitular height, diameter of the base of the capitulum, carina and scutum distance, scutal length, scutal width, tergal length and tergal width were taken following the method described by Igić (2007). For deep-water barnacles, capitular height, capitular width, peduncular length, orifice height, number of crests, capitular thickness, and peduncular width were taken following the method described by Chan et al. (2009b).

### Molecular phylogenetic analyses

We performed molecular phylogenetic analyses including new DNA sequences from our new samples from the Moluccas in combination with sequences of multiple barnacle specimens retrieved from GenBank. Our aim with the molecular analyses was not to provide a robust phylogeny of barnacles or to develop DNA barcodes for Moluccan barnacle taxa. Instead, our goal was to confirm the molecular taxonomic identity of the barnacles from the Moluccas with the published sequences in the GenBank, to examine whether they cluster near to congeneric or conspecific accessions. With this exercise we aimed to gain insights into the taxonomic positions of Moluccan barnacles in addition to those we may gain from morphology.

Genomic DNA was extracted from the adductor muscle tissue using CTab isolation buffer following the method of Doyle and Doyle (1987), as described at http://www.geocities.com/ CapeCanaveral/8431/CTab.html. Tissue was ground and placed in 200 μl CTab buffer and 5 μl Proteinase K, homogenized by shaking, and incubated at 56 °C overnight. DNA was extracted from the lysate using a Qiagen BioSprint 96 using the manufacturer’s protocol. The purified DNA was stored at –20 °C until required, and dilutions of 1 to 10 were used for the polymerase chain reaction (PCR).

We sequenced two gene fragments: cytochrome oxidase subunit I (COI), a rapidly evolving gene from the mitochondrial genome; and the 18S ribosomal RNA gene (18S), a slowly evolving and generally highly conserved gene from the ribosome. We chose these markers because of their contrasting evolutionary rates, but also because they have been widely used in barnacle phylogenetic analyses (Pérez-Losada et al. 2008, 2014; Tsang et al. 2014, 2015).

COI was sequenced using primers LCO1490 (5’-GGT CAA CAA ATC ATA AAG ATA TTG G-3’) and HCO2198 (5’-TAA ACT TCA GGG TGA CCA AAA AAT CA-3’) (Folmer et al. 1994), and 18S using primers ai (5’-CCT GAG AAA CGG CTA CCA CAT C-3’) and 7R (5’-GCA TCA CAG ACC TGT TAT TGC-3’) (Whiting 2002). PCR was performed in 25 μl volumes containing 17.8 µl ddH2O, 2.5 µl 10 × Puffer, 1 µl Mg (25mM), 0.5 µl dNTP Mix, 0.5 µl of each primer, and 0.2 µl Taq Polymerase (2.00 units/25 µl PCR) with 2 µl DNA. The reaction conditions of PCR were 94 °C for 3 minutes before 35 cycles of amplification, with 94 °C for 30 sec, 55 °C for 1 minute and 72 °C for 60 sec, followed by a final extension at 72 °C for 5 minutes. PCR products were sent to Macrogen Europe for cycle sequencing of both strands of each gene.

Chromatograms were edited using CodonCode Aligner version 5.1.5 (http://www.codoncode.com) for COI and Geneious 11 (http://www.geneious.com) for 18S. All new DNA sequences generated for this study are deposited in GenBank under the accession numbers provided in Suppl. material 1: Table S1.

For comparison, 84 COI sequences and 88 18S sequences of related barnacles were downloaded from GenBank (accession numbers are provided in Figs 28, 29). Sequences were aligned using Muscle (Edgar 2004) as implemented in Geneious and later exported as nexus or fasta files.

Phylogenetic trees were reconstructed for each gene using both Maximum Likelihood (ML) and Bayesian Inference (BI). ML analyses were conducted with RAxML Black Box (Stamatakis et al. 2008) with 100 bootstrap replicates and under the GTR + I + G model of sequence evolution. Bayesian analyses were conducted in BEAST 2. As our goal was to obtain a topology and not dates for the branching events, we used a Bayesian relaxed lognormal clock with a rate of 1. Therefore, the ages obtained in the ultrametric trees emerging from this analysis are relative not absolute. Substitution model selection was performed in jModeltest (Posada 2008) using the Akaike information criterion, and GTR + I + G was identified as the best model for both genes. For each analysis, we ran two independent chains of between 10 and 40 million generations, with a birth-death tree prior. Convergence of chains and burn-ins were assessed with Tracer, runs combined using LogCombiner, and maximum clade credibility trees produced in Tree Annotator.

Genetic distances (K2P) were calculated by MEGA version X (Kumar et al. 2018).

## Results

### Checklist tabulation

This study provides the most comprehensive overview of barnacle species from the Moluccan islands (Table 1). The morphological analyses of the collected material revealed 24 intertidal species and one deep-water species from three families of stalked barnacles (Heteralepadidae: one genus and species; Lepadidae: two genera and species; Pollicipedidae: one genus and species) and four families of acorn barnacles (Pachylasmatidae: one genus and species; Chthamalidae: five genera and species; Tetraclitidae: five genera and eight species; Balanidae: two genera and seven species).

Including previous records from the literature, we found a total of 97 species from the Moluccan islands (Table 1) from the superorder Thoracica (free living or epizoic). Of these, 21 are new records and two (*Amphibalanus* sp. and *Microeuraphia* sp.) are currently unidentified species.

All specimens obtained from field work, except for one floating specimen, were attached to types of natural and artificial substrates (Table 1), and several smaller species, e.g., *Chthamalus moro*, were also attached to other larger barnacle species, e.g., *Megabalanus tintinnabulum*. In general, the smallest species was *C. moro* (basal length: 2.4–5.1 mm; basal width: 1.4–4.1; height 0.8–1.7 mm), and the largest *M. tintinnabulum* (basal length: 26.0–49.3 mm; basal width 29.0–43.1 mm; height 20.1–49.4 mm).

### Systematic account of the 25 species morphologically examined for this study

**Class Hexanauplia Oakley, Wolfe, Lindgren & Zaharoff, 2013**

**Subclass Thecostraca Gruvel, 1905**

**Infraclass Cirripedia Burmeister, 1834 (= Cirrhipèdes Lamarck, 1806)**

**Superorder Thoracica Darwin, 1854**

**Order Lepadiformes Buckeridge & Newman, 2006**

**Suborder Heteralepadomorpha Newman, 1987**

**Family Heteralepadidae Nilsson-Cantell, 1921**

**Genus *Heteralepas* Pilsbry, 1907**

#### Heteralepas japonica

Taxon classificationAnimaliaLepadiformesHeteralepadidae

(Aurivillius, 1892)

88DBB493-E1A4-59EE-8135-3CA40F6507A8

**Figure 3.**
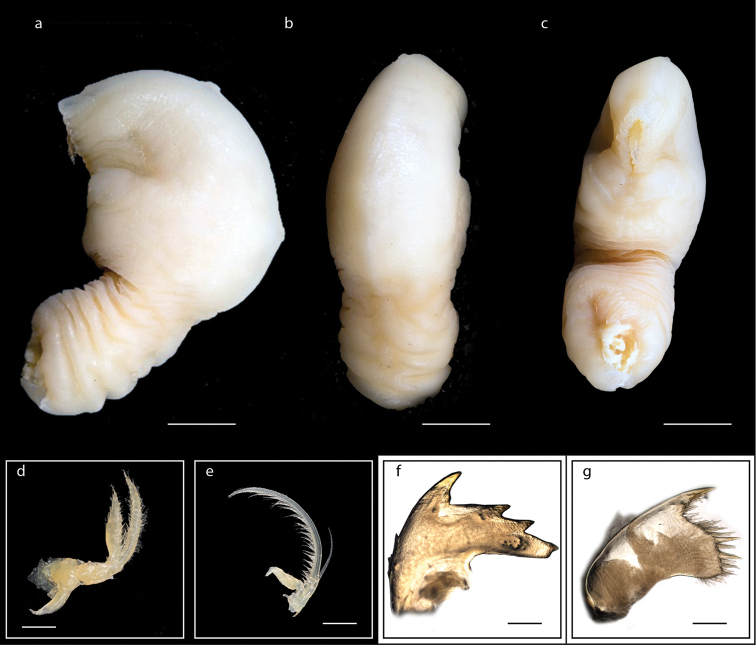
*Heteralepas japonica* (Aurivillius, 1892) (MZB Cru Cir 050- 2) **a** side view showing the capitulum and peduncle **b** upper view showing the capitulum and peduncle **c** side view showing the opening of operculum **d** cirrus I showing the filamentary appendage at the basal region **e** cirrus VI showing the caudal appendage **f** mandible **g** maxillule. Scale bars: 5 mm (**a–c**); 2 mm (**d**); 3 mm (**e**); 0.5 mm (**f–g**).

Figure 3a–g , Table 1: species no. 1

Alepas japonica Aurivillius, 1892: 125: Aurivillius 1894: 28, pl. II figs 14, 15, pl. VIII figs 3, 7, pl. IX fig. 3.
Alepas indica Gruvel, 1901: 259: Gruvel 1905a: 162, fig. 179.
Heteralepas (Heteralepas) japonica : Pilsbry 1907a: 101.
Heteralepas (Heteralepas) japonica var. alba Krüger, 1911a: 34, pl. 1 fig. 2b.
Heteralepas (Heteralepas) dubia Broch, 1922: 288, fig. 38.
Heteralepas japonica : Pilsbry 1911a: 71, fig. 4; Zevina et al. 1992: 31, fig. 19; Chan et al. 2009a: 61; Chan et al. 2009b: 88–91, figs 2A–D, 3A–D, 4, 5.

##### Material examined.

***Deep-Sea***: 32 specimens, MZB Cru Cir 050, Stn.23 Mooring Lifamatola, 250 m, coll. N. Fitriya, 9 Nov 2016; 13 specimens, MZB Cru Cir 051, Stn.39 Mooring Halmahera Seas, 250 m, coll. N. Fitriya, 13 Nov 2016; 27 specimens, MZB Cru Cir 052, Stn.56 MO2, 200 m, coll. N. Fitriya, 23 Nov 2016; 42 specimens, MZB Cru Cir 053, Stn.58 MO3, 250 m, coll. N. Fitriya, 25 Nov 2016.

##### GenBank accession numbers.

COI gene (MK995372), 18S (MK981386).

##### Diagnosis.

Capitulum rounded without hard valves and opercular plates, wall of capitulum tick with crest not more than two on the carinal region; cirrus I with filamentary appendage at the basal region; anterior rami shorter than posterior rami in cirri V–VI; caudal appendage present; maxillule strongly notched.

##### Description.

Orifice slightly protuberant, crenulated, occupying one half to one third capitular length, parallel to or at oblique angle to capitulum; integument thick, chitinous; carinal margin sometimes with warty protuberances on slight keel; peduncle naked; colour of capitulum and peduncle yellowish (Fig. 3a–c). Cirrus I with anterior rami (19-segmented) shorter than posterior rami (25-segmented) and a filamentary appendage present at the basal region (Fig. 3d); cirri II – IV long, slender, anterior rami of cirri V and VI shorter than posterior rami; cirrus VI has a caudal appendage with 20-segmented and one fourth length of anterior ramus (Fig. 3e). Mandible with four large teeth excluding inferior angle (Fig. 3f); maxillule strongly notched with two big teeth on upper angle and blade-shaped setae on cutting margin (Fig. 3g); labrum concave, teeth numerous. Ranges of height of capitulum 11.9–18.6 mm, width 8.7–15.3 mm, thickness 7.0–13.3 mm; length of peduncle 7.7–27.6 mm and width 5.1–10.4 mm (measurements for 25 specimens are presented in Suppl. material 1: Table S2).

##### Distribution.

*Heteralepas japonica* is widely distributed in Indo-west Pacific: Indian Ocean; Australia; Singapore, Malacca Str., Indonesia; Malay Archipelago; Vietnam; Condor Island; S China Sea; E China Sea; Taiwan, Philippines; S Japan; NE New Zealand; fouling hard rock substrata, crabs, gorgonians, antipatharians, deep-sea cables; 48–500 m (Jones and Hosie 2016). In this study, *Heteralepas japonica* was found attached to cable moorings in Lifamatola Sea and Halmahera Sea (a map with the occurrence of *Heteralepas japonica* in the Moluccas is shown in Suppl. material 1: Fig. S1).

##### Remarks.

The external appearance of this species is extremely variable (Nilsson-Cantell 1927). After an extensive study of *H. japonica*, Nilsson-Cantell could not distinguish *H. japonica* and *H. indica* (Gruvel, 1901) and placed the latter in synonymy with *H. japonica*, and later authors have followed this suggestion (e.g., Broch 1931, Utinomi 1958). At the same time, Nilsson-Cantell (1927) also suggested that *H. nicobarica* Annandale, 1909, *H. gigas* Annandale, 1905 and *H. cygnus* Pilsbry, 1907 could be invalid species and future revision may synonymize some or all of them. Zullo and Newman (1964) pointed out the uncertainty surrounding the status of several of the species assigned to *Heteralepas* due to a lack of zoogeographic and morphological data, since extensive collections are unavailable. Furthermore, Foster (1978) suggested that a revision of the genus was called for since the variability of *H. japonica*, as noted by Foster and tabulated by Nilsson-Cantell (1927), encompasses characters which have been used to distinguish several different species by other authors (e.g., *H. dubia* Broch, 1922, *H. cornuta* Darwin, 1852 , *H. indica* Gruvel, 1901, *H. lankestri* Gruvel, 1900).

#### Suborder Lepadomorpha Pilsbry, 1916

**Family Lepadidae Darwin, 1852**

**Genus *Dosima* Gray, 1825**

##### Dosima fascicularis

Taxon classificationAnimaliaLepadiformesLepadidae

(Ellis & Solander, 1786)

82698B85-28C2-5ADF-A7FF-0B860BD44CD0

**Figure 4.**
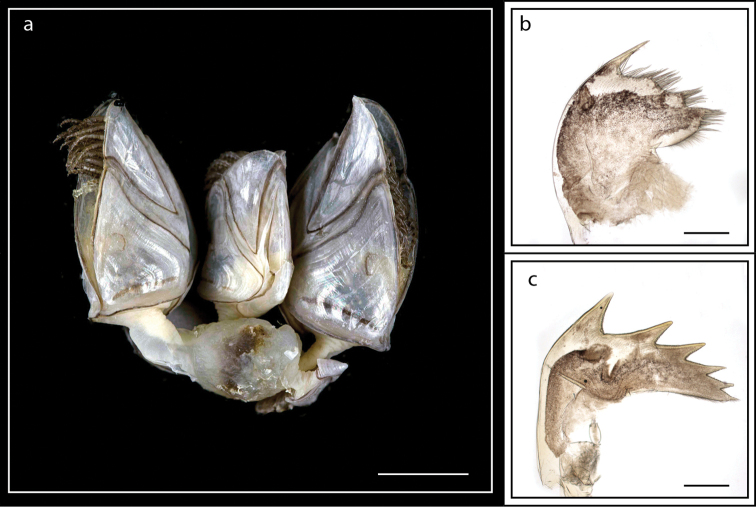
*Dosima fascicularis* (Ellis & Solander, 1786) (MZB Cru Cir 048-19) **a** side view showing the capitulum and peduncle **b** maxillule **c** mandible. Scale bars: 5 mm (**a**); 0.5 mm (**b–c**).

Figure 4a–c , Table 1: species no. 12

Lepas fascicularis Ellis & Solander, 1786: 197, tab. 15 fig. 6; Darwin 1852: 92, pl. 1 fig. 6.
Lepas fascicularis aurivillii Nilsson-Cantell, 1921: 238, fig. 40b.
Lepas cygnea Spengler, 1790: pl. 6 fig. 8.
Pentalasmis spirulicola , P. donovani Leach, 1818: 413.
Pentalasmis fascicularis : Brown 1844: pl. 51 fig. 2.
Lepas fasciculatus : Pilsbry 1907: 81, pl. IX fig.6.
Lepas (Dosima) fascicularis : Weisbord 1979: 28, pl. 2 figs 10–11; Jones et al. 1990: 8.
Dosima fascicularis : Gray 1825: 100; Zevina 1982: 21, fig. 11.

###### Material examined.

***Ambon Island***: 19 specimens, MZB Cru Cir 048, Tial, 3°38'10.2"S, 128°20'46.9"E, coll. Adin, 19 Sep 2017.

###### GenBank accession numbers.

COI gene (MK995371), 18S (MK981385).

###### Diagnosis.

The only pelagic barnacle with its own gas-filled float; plates very thin and paper-like; carina angle bent with a prominent umbo and expanded basal disk; cirri acanthopod.

###### Description.

Five capitular plates, white, thin, delicate, wide interspaces between dark purple; base of carina almost round, not imbedded in membrane, distinct angle formed at sub-central carinal umbo peduncle short, naked (Fig. 4a); five filamentary appendages located at base of cirri on each side of body; caudal appendages small, smooth, summits rounded; mandible with five teeth (Fig. 4c); penis hirsute. Ranges of diameter of capitulum base 4.0–6.8 mm; capitular height 10.5–17.1 mm; total height 11.7–19.5 mm; scutal width 5.4–7.3 mm; scutal length 8.1–11.1 mm; tergal width 2.7–4.4 mm; tergal length 6.2–11.1 mm (measurements for six specimens are presented in Suppl. material 1: Table S3).

###### Distribution.

*Dosima fascicularis* is cosmopolitan in tropical and temperate seas (Jones and Hosie 2016). While it has been reported found at New Zealand, South Africa and South America (Newman and Ross 1971). In the present study, *D. fascicularis* was found at Ambon Island at Tial (floating in water at the beach) (a map with the occurrence of *Dosima fascicularis* in the Moluccas is shown in Suppl. material 1: Fig. S2).

###### Remarks.

*Dosima fascicularis* is the only pelagic barnacle that produces its own gas-filled float enabling it to sustain itself on the sea surface (Weisbord 1979). *Dosima* can also be distinguished from members of the genus *Lepas* by the distinct angle formed at the sub-central umbo of the carina, and by very thin and brittle plates (Hinojosa et al. 2006).

##### Genus *Lepas* Linnaeus, 1758

**Subgenus Anatifa Bruguière, 1789**

###### Lepas anserifera

Taxon classificationAnimaliaLepadiformesLepadidae

Linnaeus, 1767

DFD78124-8B06-5F82-9EB5-A3166B674745

**Figure 5.**
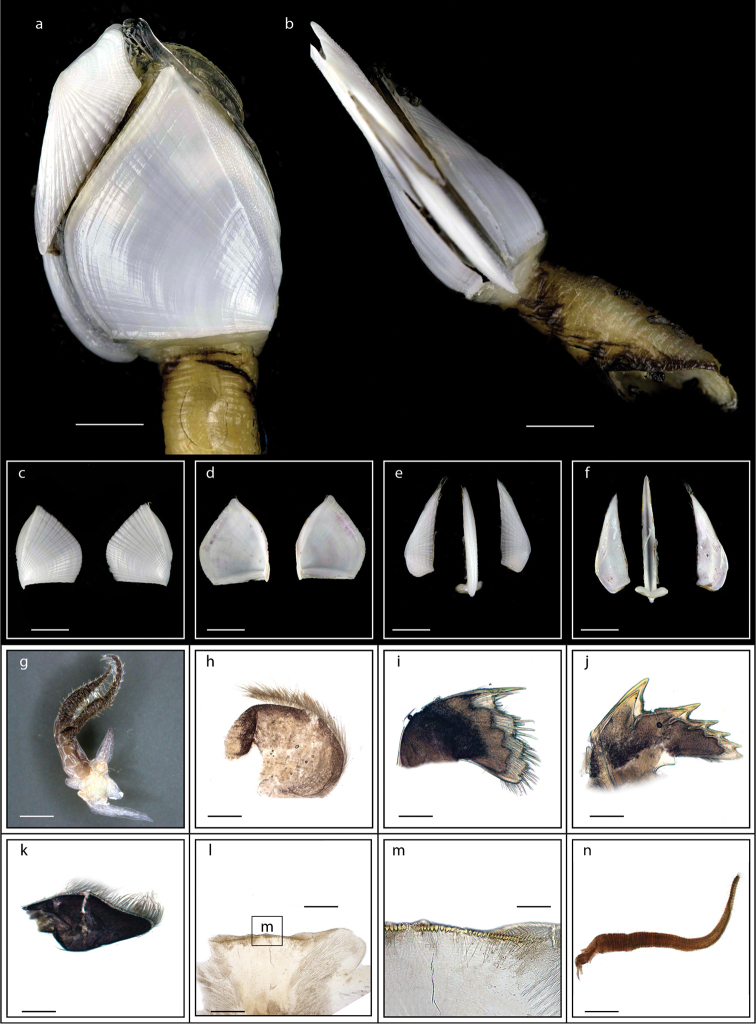
*Lepas anserifera* Linnaeus, 1767 (MZB Cru Cir 058-2) **a** side view showing the capitulum and peduncle **b** side view showing the carina **c** external view of scutum **d** internal view of scutum **e** external view of tergum and carina **f** internal view of tergum and carina **g** cirrus I **h** maxilla **i** maxillule **j** mandible **k** mandibular palp **l** labrum **m** close up view on the teeth of labrum **n** penis. Scale bars: 4 mm (**a–b**); 3 mm (**c–f**); 1 mm (**g, n**); 0.5 mm (**h–m**).

Figure 5a–n , Table 1: species no.13

Anatifa striata Bruguière, 1789: pl. 166 fig. 3.
Pentalasmis anseriferus : Brown 1844: pl. 51 fig. 1.
Lepas anserifera Linnaeus, 1767: 1109; Darwin 1852: 81, pl. 1 fig. 4; Hoek 1907: 2; Hiro 1937a: 57, fig. 48; Utinomi 1949: 20; Stubbings 1967: 237; Newman 1971: 32, fig. 1; Dong et al. 1982: 73; Zevina et al. 1992: 14, fig. 6; Igic 2007: 37, fig. 10; Chan et al. 2009a: 45, fig. 34; Keable and Reid 2015: 266.

####### Material examined.

***Ambon Island***: 5 specimens, MZB Cru Cir 056, Galala, 3°41'22.2"S, 128°10'52.6"E, coll. P. Pitriana & D. Tala, 6 Sep 2016; 8 specimens, MZB Cru Cir 057, Laha, 3°43'22.5"S, 128°05'02.5"E, coll. P. Pitriana & D. Tala, 5 Sep 2016; 74 specimens, MZB Cru Cir 058, Suli, 3°37'02.0"S, 128°16'31.6"E, coll. Adin, 19 Sep 2017; 93 specimens, MZB Cru Cir 059, Tial, 3°38'10.2"S, 128°20'46.9"E, coll. Adin, 19 Sep 2017. ***Pombo Island***: 5 specimens, MZB Cru Cir 060, Pombo, 3°31'55.5"S, 128°22'28.8"E, coll. P. Pitriana & D. Tala, 8 Sep 2016. ***Seram Island***: 13 specimens, MZB Cru Cir 061, Lepas Pantai Kawa, 2°57'32.5"S ,128°05'33.4"E, coll. P. Pitriana & D. Tala, 19 Sep 2017; 10 specimens, MZB Cru Cir 062, Desa Murnaten, 2°51'48.8"S, 128°20'32.3"E, coll. P. Pitriana & D. Tala, 20 Sep 2017; 10 specimens, MZB Cru Cir 063, Desa Kasie, 2°51'05.5"S, 128°32'54.1"E, coll. P. Pitriana & D. Tala, 20 Sep 2017; 10 specimens, MZB Cru Cir 064, Dermaga Pelita Jaya, 3°00'13.5"S, 128°07'09.2"E, coll. P. Pitriana & D. Tala, 21 Sep 2017. ***Saparua Island***: 19 specimens, MZB Cru Cir 065, Negeri Mahu, 3°31'52.9"S, 128°41'12.4"E, coll. P. Pitriana & D. Tala, 11 Apr 2016.

####### GenBank accession numbers.

COI gene (MK995373–MK995375), 18S (MK981387–MK981388).

####### Diagnosis.

Capitulum with five completely calcified plates; surfaces striated with radiating lines; scuta with conspicuous growth lines; scutal margin of terga without notch, occluding margin of scutum strongly convex and swollen; carina apex extending to tergum, base of carina forked; filamentary appendages and caudal appendage present.

####### Description.

Five capitular plates, closely approximate, white, slightly furrowed, terga sometimes strongly pectinated, occluding margin arched, protuberant (Fig. 5a, b). Scutum with well developed, strong internal umbonal tooth, left internal umbonal tooth small, or mere ridge; carina produced below base of scutum as fork, apex pointed (Fig. 5c–f); 5–6 filamentary appendages on each side, one on side of prosoma below base of pedicel of cirrus I, four others placed in pairs beneath basal segment of pedicel of cirrus I, lowest posterior filament of the four generally larges (Fig. 5g); caudal appendages small, smooth, curved, claw-like, tip pointed; mandible with five teeth excluding inferior angle, lower angle pectinate (Fig. 5j), labrum concave, toothed (Fig. 5l-m). Basal diameter of capitulum 2.0–7.2 mm; capitular height 8.1–14.8 mm; total height 14.2–31.9 mm. Scutal width 4.7–11.3 mm; scutal length 6.3–12.5 mm. Tergal width 4.7–11.3 mm; tergal length 4.3–9.6 mm (measurements for 25 specimens are presented in Suppl. material 1: Table S4).

####### Distribution.

*Lepas anserifera* is a cosmopolitan, pelagic species occurring in tropical and temperate oceans (Jones et al. 2001). In this study, *Lepas anserifera* was found on the islands of Ambon (at Suli, Tial, Galala, Laha), Pombo, Seram (at Lepas Pantai Kawa, Desa Murnaten, Desa Kasie, Dermaga Pelita Jaya), and Saparua (at Negeri Mahu). *Lepas anserifera* was found attached to mangroves, stone ship charts and ship walls, port poles, and shells of *Megabalanus zebra* (a map with the occurrence of *Lepas anserifera* in the Moluccas is shown in Suppl. material 1: Fig. S3).

####### Remarks.

*Lepas anserifera* can be easily recognized by the presence and positions of the 5–6 filamentary appendages and the curved caudal appendages (Igić 2007).

#### Order Scalpelliformes Buckeridge & Newman, 2006

**Suborder Scalpellomorpha Newman, 1987**

**Family Pollicipedidae Leach, 1817**

**Genus *Capitulum* Gray, 1825**

##### Capitulum mitella

Taxon classificationAnimaliaScalpelliformesPollicipedidae

(Linnaeus, 1758)

EE18154C-EF8C-5AFB-BC2F-D373CDC9E304

**Figure 6.**
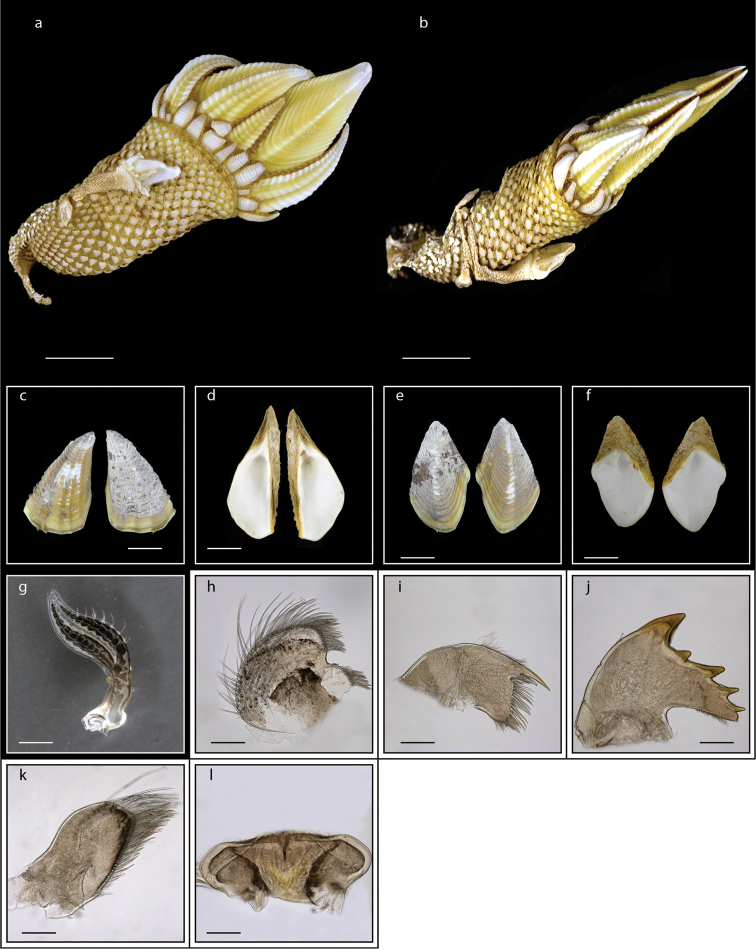
*Capitulum mitella* (Linnaeus, 1758) (MZB Cru Cir 026-4) **a** side view showing the capitulum and peduncle **b** side view showing the rostrum **c** external view of scutum **d** internal view of scutum **e** external view of tergum **f** internal view of tergum **g** cirrus I **h** maxilla **i** maxillule **j** mandible **k** mandibular palp **l** labrum. Scale bars: 6 mm (**a–b**); 2 mm (**c–f**); 1 mm (**g**); 0.5 mm (**h–l**).

Figure 6a–l , Table 1: species no.17

Lepas mitella Linnaeus, 1758: 668.
Pollicipes mitella : Sowerby 1833: fig. 2; Darwin 1852: 316, pl. VII fig. 3; Utinomi 1970: 339; Dong et al. 1982: 69; Zevina et al. 1992: 37, fig. 23.
Polylepas mitella : Blainville 1824: pl. 1 fig. 5.
Mitella mitella : Pilsbry 1907: 6; Annandale 1916: 128, pl. 12 fig. 1.
Capitulum mitella : Gray 1825: 101; Foster 1980: 209; Chan et al. 2009a: 85, fig. 70; Williamson 2014: 758, fig. 1D.

###### Material examined.

***Ambon Island***: 10 specimens, MZB Cru Cir 023, Liang, 3°30'13.3"S, 128°20'34.1"E, coll. P. Pitriana & D. Tala, 30 Aug 2016; 2 specimens, MZB Cru Cir 024, Liang, 3°30'13.3"S, 128°20'34.1"E, coll. P. Pitriana & D. Tala, 7 Sep 2016; 2 specimens, MZB Cru Cir 025, Alang, 3°45'11.0"S, 128°01'23.1"E, coll. Adin, 20 Sep 2017; 4 specimens, MZB Cru Cir 026, Asilulu, 3°40'50.4"S, 127°55'27.6"E, coll. Adin, 20 Sep 2017; 3 specimens, MZB Cru Cir 027, Dermaga Tulehu, 3°35'21.8"S, 128°20'02.8"E, coll. P. Pitriana & D. Tala, 7 Sep 2017; 15 specimens, MZB Cru Cir 028, Tawiri, 3°42'10.1"S, 128°06'13.4"E, coll. P. Pitriana & D. Tala, 29 Mar 2016; 5 specimens, MZB Cru Cir 029, Tawiri, 3°42'10.1"S, 128°06'13.4"E, coll. P. Pitriana & D. Tala, 5 Sep 2016; 2 specimens, MZB Cru Cir 030, Laha, 3°43'22.5"S, 128°05'02.5"E, coll. P. Pitriana & D. Tala, 5 Sep 2017; 6 specimens, MZB Cru Cir 031, Morella, 3°31'06.5"S, 128°13'18.0"E, coll. Adin, 20 Sep 2017; 5 specimens, MZB Cru Cir 032, Ureng, 3°40'14.0"S, 127°56'47.6"E, coll. Adin, 20 Sep 2017; 5 specimens, MZB Cru Cir 033, Wakasihu, 3°46'27.6"S, 127°56'36.6"E, coll. Adin, 20 Sep 2017. ***Saparua Island***: 10 specimens, MZB Cru Cir 034, Teluk Saparua, 3°34'25.7"S, 128°39'25.8"E, coll. P. Pitriana & D. Tala, 8 Apr 2016; 11 specimens, MZB Cru Cir 035, Benteng Durstede, 3°34'32.8"S, 128°39'34.7"E, coll. P. Pitriana & D. Tala, 8 Apr 2016.

###### Genbank accession number.

18S (MK981390).

###### Diagnosis.

Capitulum with more than 18 plates, all with apical umbones; lateral plates numerous and only one big plate under the rostrum; scales of the peduncle symmetrically arranged in close whorls.

###### Description.

Capitulum fan-shaped, with eight large plates, basal ring of 18–25 smaller plates, all plates yellowish, umbos apical; peduncle covered by numerous yellowish, fine scales (Fig. 6a, b); mandible with five teeth (Fig. 6j); labrum concave, teeth lacking (Fig. 6l). Capitular diameter base ranges 4.5–20.4 mm; total height 10.7–47.1 mm; capitular height 7.0–24.6 mm. Ranges of distance from rostrum to carina 5.8–27.3 mm and rostral height 5.5–24.2 mm (measurements for 25 are presented in Suppl. material 1: Table S5).

###### Distribution.

Darwin (1852) reported *Capitulum mitella* from the Philippine Archipelago, Ambon, East Indian Archipelago and Madagascar. Chan et al. (2009) and Jones and Hosie (2016) reported the species as widely distributed in warmer parts of the Indo-Pacific region, from Madagascar to southern Japan. In this study, *C. mitella* was found on the islands of Ambon (at Ureng, Alang, Dermaga Liang, Asilulu, Doc. Tawiri, Morella, Wakasihu, Laha, and Tulehu) and Saparua (at Benteng Duurstede and Teluk Saparua). *Capitulum mitella* attach on rocks, stone, wall of fortress, port pole and concrete wall (a map with the occurrence of *Capitulum mitella* in the Moluccas is shown in Suppl. material 1: Fig. S4).

###### Remarks.

*Capitulum mitella* is the famous Japanese goose barnacle or ‘kame-no-te’ (meaning the hand of the turtle, referring to its shape). This barnacle is edible and sold as an expensive seafood in Japan, China, Taiwan, and Korea, as well as in Portugal and Spain, where it is known as ‘percebes’.

#### Order Sessilia Lamarck, 1818

**Suborder Balanomorpha Pilsbry, 1916**

**Family Pachylasmatidae Utinomi, 1968**

**Subfamily Pachylasmatinae Utinomi, 1968**

**Genus *Pseudoctomeris* Poltarukha, 1996**

##### Pseudoctomeris sulcata

Taxon classificationAnimaliaSessiliaPachylasmatidae

(Nilsson-Cantell, 1932)

C39A0C98-3DD1-53DC-810C-F22BD5F872E4

**Figure 7.**
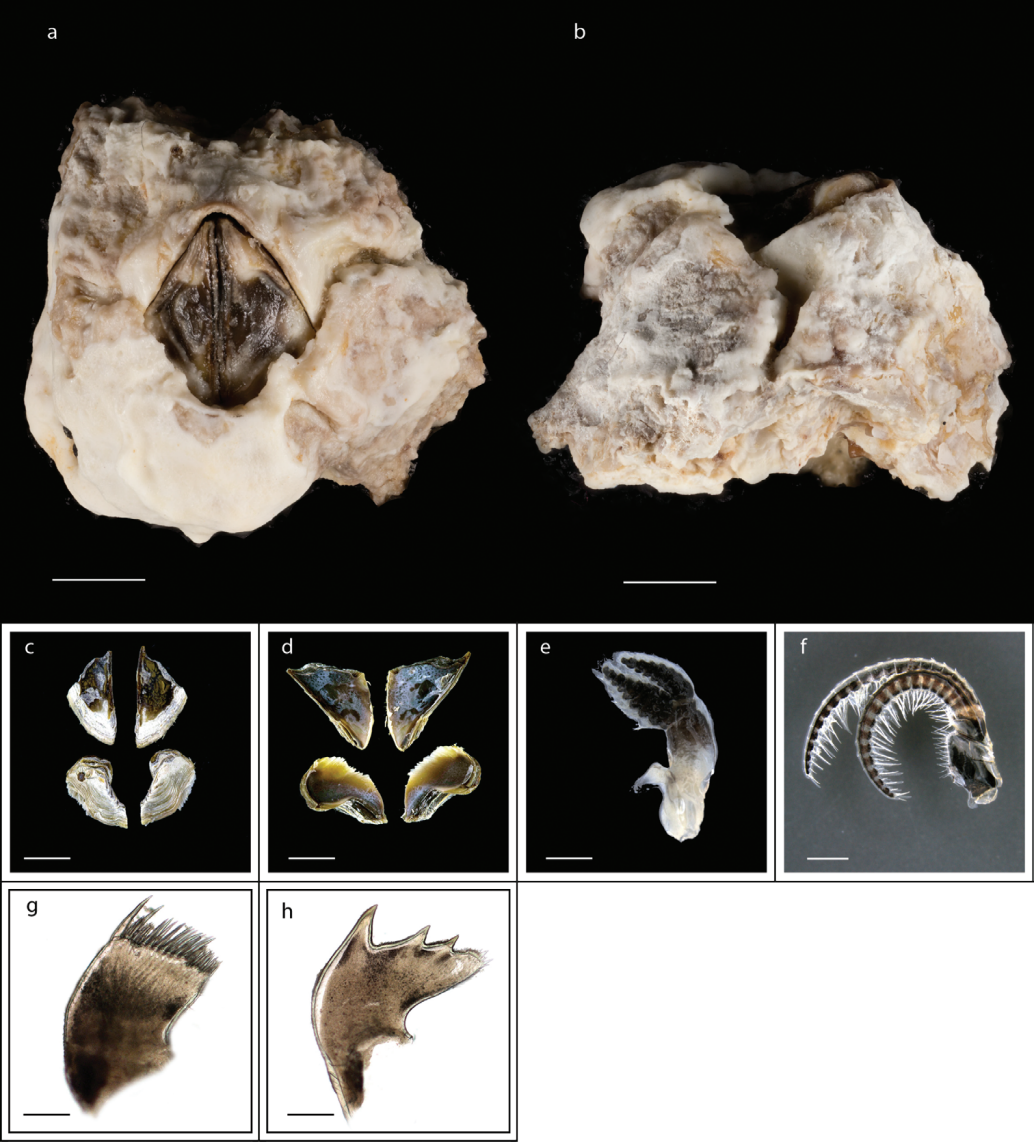
*Pseudoctomeris sulcata* (Nilsson-Cantell, 1932) (MZB Cru Cir 073-4) **a** upper view **b** side view **c** external view of scutum and tergum **d** internal view of scutum and tergum **e** cirrus I **f** cirrus III **g** maxillule **h** mandible. Scale bars: 4 mm (**a–b**); 2 mm (**c–d**); 1 mm (**e**); 2 mm (**f**), 0.5 mm (**g–h**).

Figure 7a–h , Table 1: species no. 50

Octomeris sulcata Nillson-Cantell, 1932: 8; Newman and Ross 1976: 40.
Pseudoctomeris sulcata : Poltarukha 1996: 988; Chan et al. 2009a: 156, fig. 131.

###### Material examined.

***Ambon Island***: 4 specimens, MZB Cru Cir 073, Leahari, 3°42'45.3"S, 128°16'16.5"E, coll. P. Pitriana, 14 Jan 2016; 2 specimens, MZB Cru Cir 074, Hatu, 3°43'52.7"S, 128°02'51.4"E, coll. Adin, 20 Sep 2017.

###### Diagnosis.

Shell with eight plates; compound rostrum; scutum and tergum fused; mandible tridentate; multi-jointed caudal appendage present.

###### Description.

Shell externally white, internally black; eight plated, rostrum partially fused with rostrolaterals giving external appearance of six plates (Fig. 7a–b); basis membranous, calcareous; orifice rhomboidal; scutum and tergum thick (Fig. 7c, d); maxilla triangular, maxillule not notched (Fig. 7g), mandible with three teeth (Fig. 7h), labrum concave, with blunt teeth; cirrus VI with long, multi-segmented caudal appendages. Ranges of basal length 16.2–16.7 mm, basal width 11.5–15.0 mm, height 7.4–7.7 mm. Orifice length 5.5–7.9 mm, orifice width 4.3–6.1 mm (measurements for two specimens are presented in Suppl. material 1: Table S6).

###### Distribution.

*Pseudoctomeris sulcata* was previously recorded from southern Japan, China, and Taiwan (Jones et al. 2001; Poltarukha and Zevina 2006). In this study, *P. sulcata* was found on Ambon Island at Leahari and Hatu on rocks and shells of *Tetraclita squamosa* (a map with the occurrence of *Pseudoctomeris sulcata* in the Moluccas is shown in Suppl. material 1: Fig. S2).

###### Remarks.

Externally, the fused rostrum and rostrolaterals are six-plated, but the sutures are visible internally (Poltarukha 1996). Morphologically, *Pseudoctomeris sulcata* shows features of the scutum and tergum similar to those of representatives of the family Pachylasmatida. However, the species can be distinguished by its tridentate mandible and the presence of multi-jointed caudal appendages (Poltarukha 2006). A previous molecular study showed that *P. sulcata* clustered together with members of the family Pachylasmatidae, not with members of the Chthamalidae (Chan et al. 2017). According to Chan et al. (2017), *P. sulcata* is an intertidal species of the Pachylasmatidae, previously believed to be an exclusive deep-sea taxon.

##### Superfamily Chthamaloidea Darwin, 1854

**Family Chthamalidae Darwin, 1854**

**Subfamily Notochthamalinae Foster & Newman, 1987**

**Genus *Hexechamaesipho* Poltarukha, 1996**

###### Hexechamaesipho pilsbryi

Taxon classificationAnimaliaSessiliaChthamalidae

(Hiro, 1936)

9F232037-2FD7-583B-8E64-E10757B95BFB

**Figure 8.**
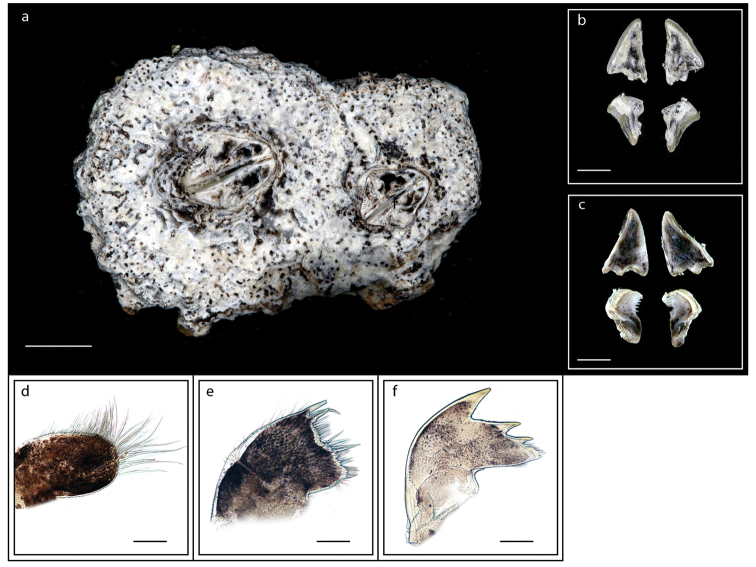
*Hexechamaesipho pilsbryi* (Hiro, 1936) (MZB Cru Cir 055) **a** upper view **b** external view of scutum and tergum **c** internal view of scutum and tergum **d** maxilla **e** maxillule **f** mandible. Scale bars: 4 mm (**a**); 2 mm (**b–c**); 0.5 mm (**d–f**).

Figure 8a–f , Table 1: species no. 51

Chthamalus pilsbryi Hiro, 1936: 227, fig. 3.
Euraphia pilsbryi : Newman & Ross, 1976: 41.
Hexechamaesipho pilsbryi : Poltarukha 1996: 989; Poltarukha 2006: 73–74; Chan et al. 2008: 320, fig. 3; Chan et al. 2009a: 149, fig. 125; Tsang et al. 2013: 188.

####### Material examined.

***Ambon Island***: 20 specimens, MZB Cru Cir 054, Hila, 3°34'57.5"S, 128°05'31.9"E, coll. Adin, 20 Sep 2017; 1 specimen, MZB Cru Cir 055, Hatu, 3°43'52.7"S, 128°02'51.4"E, coll. Adin, 20 Sep 2017.

####### Diagnosis.

Shell with six plates; surface grey with black spots scattered; scutum and tergum deeply interlock forming a sinuous line; cirri I and II with multi-cuspid setae.

####### Description.

Surface of parietes grey or light brown in colour and spotted with black; orifice rhomboidal (Fig. 8a); basis calcareous; scutum and tergum strongly articulated, forming sinuous line; scutum elongated, triangular, tergal margin strongly articulated tergum narrow, basi-scutal angle almost 90° (Fig. 8b–c); cirrus II with multi-cuspid setae; mandible with three large teeth (Fig. 8f), labrum with row of large teeth. Basal length 8.9–17.0 mm, basal width 10.3–16.4 mm, height 1.0–3.7 mm. Orifice length 4.2–6.9 mm, orifice width 3.6–5.6 mm (measurements for ten specimens are presented in Suppl. material 1: Table S7).

####### Distribution.

Previously, *Hexechamaesipho pilsbryi* was reported from Japan (Honshu, Shimoda, Wakayama, Okinawa); Taiwan (Turtle Island, Da Xiang Lang, Shi Ti Ping, Kenting); Philippines (Puerto Galera, Tiwi-Bicol, Boracay); Malaysia (Nexus Beach, Kota Kinnabalu, Sabah) (Tsang et al. 2013). In the present study, *H. pilsbryi* was collected from Hatu and Hila on Ambon Island (a map with the occurrence of *Hexechamaesipho pilsbryi* in the Moluccas is shown in Suppl. material 1: Fig. S2). A previous study of *H. pilsbryi* indicated that the distribution of this species bridges the junction of the Japan region and the Indo-Polynesian province of Briggs (Briggs 1974). Molecular results of *H. pilsbryi* analysed by Tsang et al. (2013) suggested that this species can be divided into two highly diverged lineages: (1) a northern lineage, predominantly distributed in Japan and Okinawa, and (2) a southern lineage, primarily distributed in Taiwan and Southeast Asia. Assuming that we have molecular data of *H. pilsbryi* in our samples, there is a probability that our samples from the Moluccas include members of the southern lineage.

####### Remarks.

*Hexechamaesipho pilsbryi* was first identified from Japan as *Chthamalus pilsbryi* Hiro, 1936. However, due to the presence of three large teeth on the mandible, a characteristic of the subfamily Euraphiinae, the species was placed in the genus *Euraphia* (Nilsson-Cantell 1921). Later, due to the presence of multicuspidate setae on cirrus II, Poltarukha (1996) moved *E. pilsbryi* to the sub-family Notochthamalinae and determined a new genus, *Hexechamaesipho*, which had six parietes and a deeply interlocking scutum and tergum. Currently, *H. pilsbryi* is the only species in the genus.

###### Genus *Nesochthamalus* Foster & Newman, 1987

####### Nesochthamalus intertextus

Taxon classificationAnimaliaSessiliaChthamalidae

(Darwin, 1854)

842F5560-29DB-57A9-A42A-3A97A667814C

**Figure 9.**
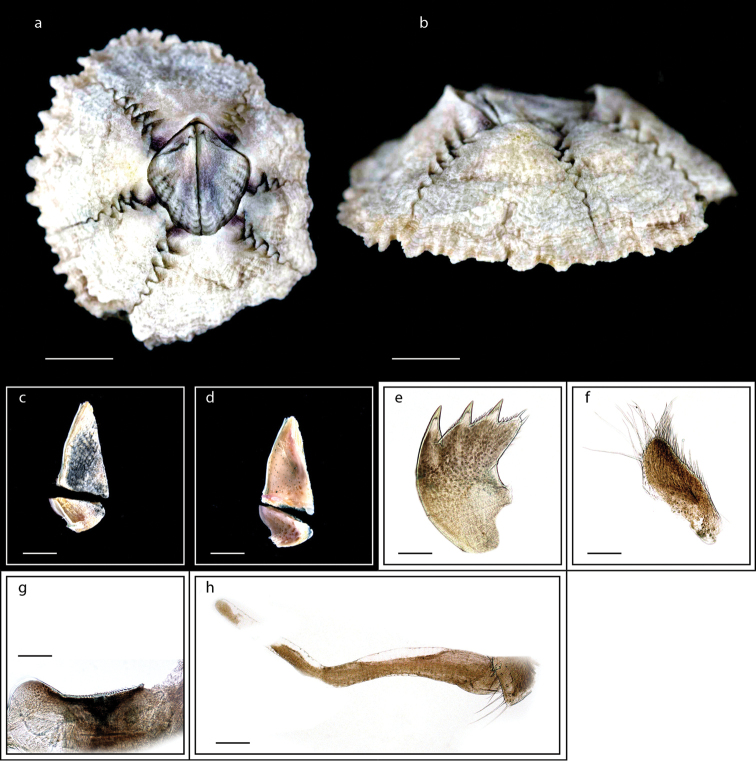
*Nesochthamalus intertextus* (Darwin, 1854) (MZB Cru Cir 070-5) **a** upper view **b** side view **c** external view of scutum and tergum **d** internal view of scutum and tergum **e** mandible **f** mandibular palp **g** labrum **h** penis. Scale bars: 3 mm (**a**); 0.75 mm (**c–d**); 0.25 mm (**e–g**); 1 mm (**h**).

Figure 9a–h , Table 1: species no. 52

Chthamalus intertextus Darwin, 1854: 467, pl. 19 figs 1a, b; Dong et al. 1982: 82; Pope 1965: 29, pl. I figs 1f, 3a–d.
Euraphia intertextus : Newman & Ross, 1976: 41; Zevina et al. 1992: 79, fig. 53.
Nesochthamalus intertextus : Foster & Newman, 1987: 326, fig. 3; Southward et al. 1998: 120, fig. 1D, 1H; Chan et al. 2009a: 147, fig. 124.

######## Material examined.

***Ambon Island***: 5 specimens, MZB Cru Cir 070, Laha, 3°43'22.5"S, 128°05'02.5"E, coll. P. Pitriana & D. Tala, 7 Sep 2016; 5 specimens, MZB Cru Cir 071, Hila, 3°34'57.5"S, 128°05'31.9"E, coll. Adin, 20 Sep 2017.

######## GenBank accession numbers.

COI gene (MK995376), 18S (MK981389).

######## Diagnosis.

Shell depressed with large diamond-shaped orifice; scutum and tergum fused; external radii consist of oblique laminae arising on both sides of the sutures, standing nearly parallel to the parietes, interfolding with each other; cirri II and III with multi-cuspid setae.

######## Description.

Shell with six plates, oval, flattened, colour of external shell white to pale grey, interior of shell violet; orifice rhomboidal; parietal sutures with conspicuous interlocking pattern (Fig. 9a, b); basis membranous with partial secondary calcification with age; opercular plates fused but separable (Fig. 9c, d); cirrus I with rami unequal; mandible with three large teeth (Fig. 9e), mandibular palp with long setae on exterior basal margin (Fig. 9f); labrum strongly dentate (Fig. 9g). Basal length 8.9–12.1 mm, basal width 6.9–10.3 mm, height 1.3–3.1 mm. Orifice length 3.0–4.9 mm, orifice width 2.6–3.9 mm (measurements for ten specimens are presented in Suppl. material 1: Table S8).

######## Distribution.

*Nesochthamalus intertextus* is known from islands in the West and Central Pacific Ocean – Indonesia, New Guinea, Malaysia to Vietnam; China; Taiwan; Philippines; Japan; Hawaii; Pitcairn I (Pope 1965; Newman and Ross 1976; Chan et al. 2009; Jones and Hosie 2016). In this study, *N. intertextus* was found on Ambon Island at Laha and Hila on stone (a map with the occurrence of *Nesochthamalus intertextus* in the Moluccas is shown in Suppl. material 1: Fig. S2).

######## Remarks.

*Nesochthamalus intertextus* can be distinguished by the conspicuous interlocking pattern exhibited by the parietal sutures and features of the basis, which is membranous in young specimens but becomes secondarily calcified with age, leaving a membranous centre only (Poltarukha 2008; Pope 1965).

###### Family Chthamalidae Darwin, 1854

**Subfamily Euraphiinae Newman & Ross, 1976**

**Genus *Euraphia* Conrad, 1837**

####### Euraphia hembeli

Taxon classificationAnimaliaSessiliaChthamalidae

Conrad, 1837

DCA203B8-0B88-5BAC-B47F-CB81239D7EE3

**Figure 10.**
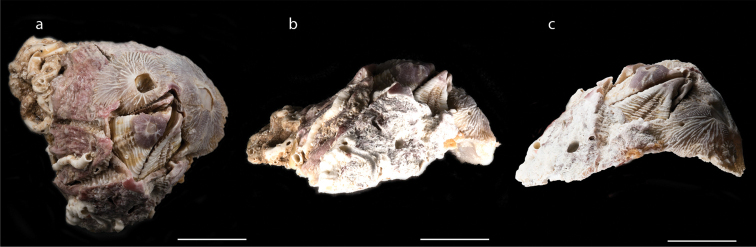
*Euraphia hembeli* Conrad, 1837 (MZB Cru Cir 049) **a** upper view of *E. hembeli* which is overgrown with other species of barnacles in its operculum **b** right side view **c** left side view. Scale bar: 16 mm.

Figure 10a–c , Table 1: species no. 53

Chthamalus hembeli Darwin, 1854: 465, fig. 5a–5d; Pilsbry 1916: 324.
Euraphia hembeli Conrad, 1837: 261, pl.20 fig.6; Newman and Ross 1976: 41; Foster and Newman 1987: 330; Southward et al. 1998: 120, fig. 1E; Paulay and Ross 2003: 307; Jones 2012: 372; Pochai et al. 2017: 17.

######## Material examined.

***Ambon Island***: 1 specimen, MZB Cru Cir 049, Asilulu, 3°40'50.4"S, 127°55'27.6"E, coll. Adin, 20 Sep 2017.

######## Diagnosis.

Shell with interlocking teeth between plates; base with a true calcareous and complete secondary calcification; scutum higher than wide and interlocked but not concrescent with tergum.

######## Description.

Shell with six plates, parietes symmetrical, calcareous, solid, separable, due to coarsely serrate sutures with interlocking toothed structure (Fig. 10a–c); colour yellowish or brownish grey, inner surface of parietes smooth, white with dark brown and pale violet horizontal striations around aperture; external surface of shell irregularly ribbed around basal margin, basis calcareous; orifice rhomboidal; tergum and scutum separable; scutum triangular, occluding margin with strong teeth; tergum strongly marked with 10–12 lateral depressor crests, scutal margin strongly articulated. Measurements for one specimen are presented in Suppl. material 1: Table S9.

######## Distribution.

*Euraphia hembeli* has been recorded from the Mediterranean, West Africa, Indian Ocean: Ceylon; Andaman Sea, Cocos-Keeling Islands; Malay Archipelago (Sunda Islands); Pacific Ocean (Japan; Caroline Islands; Hawaiian Islands, California (Newman and Ross 1976; Jones 2012; Barrett and Freeman 2016; Pochai et al. 2017). In this study, *Euraphia hembeli* was found on Ambon Island at Asilulu on rocks (a map with the occurrence of *Euraphia hembeli* in the Moluccas is shown in Suppl. material 1: Fig. S2).

######## Remarks.

*Euraphia hembeli* has a true calcareous basis and complete secondary calcification on its parietal wall and basis (Southward et al. 1998). It can be also distinguished from other species of the genus *Euraphia* by its size (up to 30 mm) and the presence of strong marked lateral depressor crests (between 10–12 in number) (Pochai et al. 2017).

####### Genus *Microeuraphia* Poltarukha, 1997

######## Microeuraphia

Taxon classificationAnimaliaSessiliaChthamalidae

sp.

7FC79EFB-74E2-5A00-BD0A-2AB76106FBCB

**Figure 11.**
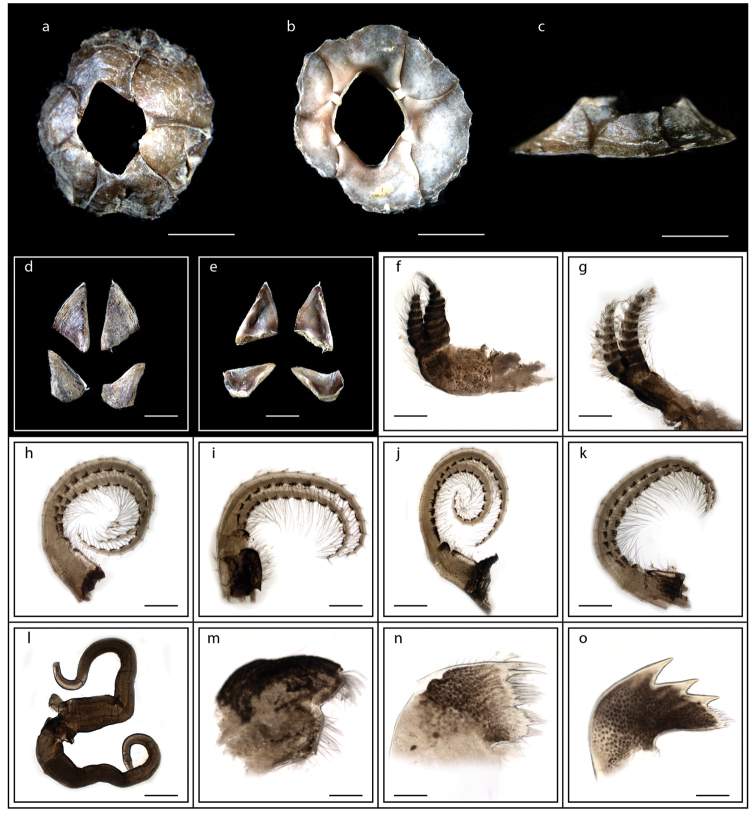
*Microeuraphia* sp. (MZB Cru Cir 136-1) **a** upper view **b** lower view **c** side view **d** external view of scutum and tergum **e** internal view of scutum and tergum **f** cirrus I **g** cirrus II **h** cirrus III **i** cirrus IV **j** cirrus V **k** cirrus VI **l** penis **m** maxilla **n** maxillule **o** mandible. Scale bars: 3 mm (**a–c**); 0.5 mm (**d–l**); 0.25 mm (**m–o**).

Figure 11a–o , Table 1: species no. 55

######### Material examined.

***Seram Island***: 2 specimens, MZB Cru Cir 138, Pantai Waimeteng-Piru, 3°04'15.3"S, 128°11'45.8"E, coll. P. Pitriana & D. Tala, 21 Sep 2017.

######### GenBank accession numbers.

COI gene (MK995389, MK995390), 18S (MK981401, MK981402).

######### Diagnosis.

Shell small with six thin plates; basis membranous; scutum and tergum remain articulated, scutum higher than wide; mandible tridentate; caudal appendage absent; one individual with two penises.

######### Description.

Shell brownish (Fig. 11a, b), depressed (Fig. 11c); orifice diamond shaped (Fig. 11a, b); overlap of ‘rostrolateral’ forming T junction (Fig. 11b); scutum and tergum triangular, tergal margins straight (Fig. 11d, e); cirrus I with anterior ramus longer than posterior (Fig. 11f); mandible with smooth tridentate teeth (11o). Ranges of basal length 3.6–9.9 mm, basal width 3.0–9.1 mm, height 1.2–2.2 mm. Orifice of diamond shape with orifice length 1.5–4.5 mm, orifice width 0.7–3.6 mm (measurements for two specimens are presented in Suppl. material 1: Table S10).

######### Distribution.

In this study, *Microeuraphia* sp. was found on Seram Island (at Pantai Waimeteng, Piru) (a map with the occurrence of *Microeuraphia* sp. in the Moluccas is shown in Suppl. material 1: Fig. S5).

######### Remarks.

*Microeuraphia* sp. clustered as a unit, forming a well-supported clade in the COI tree (Fig. 29). Morphologically, one individual of this species exhibited two penises.

###### Family Chthamalidae Darwin, 1854

**Subfamily Chthamalinae Darwin, 1854**

**Genus *Chthamalus* Ranzani, 1817**

####### Chthamalus moro

Taxon classificationAnimaliaSessiliaChthamalidae

Pilsbry, 1916

F0439AC8-BAAB-5BF8-A967-C6D534CCE38F

**Figure 12.**
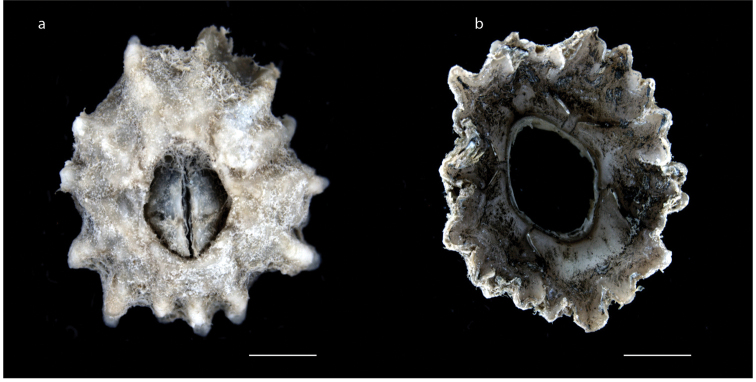
*Chthamalus moro* Pilsbry, 1916 (MZB Cru Cir 042-1) **a** upper view **b** lower view. Scale bar: 1 mm.

Figure 12a, b , Table 1: species no. 57

Chthamalus malayensis : Utinomi 1954: 18–21 (part.); Karande and Palekar 1963 (part.); Pope 1965 (part.); Newman and Ross 1976 (part.).
Chthamalus moro Pilsbry, 1916: 311; Nilsson-Cantell 1921: 277; Broch 1922: 307 (part.); Hiro 1937b: 49; Rosell 1972: 178; Dong et al. 1980: 125; Ren 1984: 153; Southward and Newman 2003: 798, fig. 2B; Chan et al. 2009a: 165, fig.141.
non Chthamalus moro Broch, 1922: 307 (part.); Broch 1931: 56 (includes a euraphiid).
non Chthamalus moro Nilsson-Cantell, 1934: 50 (a euraphiid).
non Chthamalus moro Poltarukha, 2001b: 160 (= C. malayensis).

######## Material examined.

***Ambon Island***: 2 specimens, MZB Cru Cir 036, Alang, 3°45'11.0"S, 128°01'23.1"E, coll. Adin, 20 Sep 2017; 2 specimens, MZB Cru Cir 037, Asilulu, 3°40'50.4"S, 127°55'27.6"E, coll. Adin, 20 Sep 2017; 10 specimens, MZB Cru Cir 038, Hila, 3°34'57.5"S, 128°05'31.9"E, coll. Adin, 20 Sep 2017; 7 specimens, MZB Cru Cir 039, Hatu, 3°43'52.7"S, 128°02'51.4"E, coll. Adin, 20 Sep 2017; 44 specimens, MZB Cru Cir 040, Mamala, 3°33'20.5"S, 128°11'32.8"E, coll. Adin, 20 Sep 2017; 38 specimens, MZB Cru Cir 041, Morella, 3°31'06.5"S, 128°13'18.0"E, coll. Adin, 20 Sep 2017; 25 specimens, MZB Cru Cir 042, Wakasihu, 3°46'27.6"S, 127°56'36.6"E, coll. Adin, 20 Sep 2017. ***Pombo Island***: 4 specimens, MZB Cru Cir 043, Pombo, 3°31'55.5"S, 128°22'28.8"E, coll. P. Pitriana & D. Tala, 8 Sep 2016. ***Saparua Island***: 32 specimens, MZB Cru Cir 044, Dermaga Ihamahu, 3°31'13.0"S, 128°41'14.9"E, coll. P. Pitriana & D. Tala, 11 Apr 2016; 31 specimens, MZB Cru Cir 045, Kulur, 3°29'48.5"S, 128°36'10.7"E, coll. P. Pitriana & D. Tala, 20 Sep 2016; 40 specimens, MZB Cru Cir 046, Waisisil, 3°34'48.6"S, 128°39'04.8"E, coll. P. Pitriana & D. Tala, 8 Apr 2016. ***Seram Island***: 15 specimens, MZB Cru Cir 047, Desa Murnaten, 2°51'48.8"S, 128°20'32.3"E, coll. P. Pitriana & D. Tala, 19 Sep 2017.

######## GenBank accession numbers.

COI gene (MK995377–MK995388), 18S (MK981391–MK981400).

######## Diagnosis.

Shell with six plates; rostrum and carina with radii; rostral lateral lacking radii; carinal lateral absent; base membranous; conical spines on cirrus I absent; basal guard on apex setae of cirrus II absent.

######## Description.

Shell white to grey, surface with strong, radiating lines, orifice elliptical (Fig. 12a); parietes solid (Fig. 12b); scutum triangular, tergal margin straight; tergum triangular, scutal margin curved; conical spines on dorsal side of cirrus I absent, cirrus II with multi-cuspidate setae without basal guard. Basal length 2.4–5.1 mm, basal width 1.4–4.1 mm and height 0.8–1.7 mm. Orifice length 1.0–3.4 mm and orifice width 0.7–1.7 mm (measurements for 25 specimens are presented in Suppl. material 1: Table S11).

######## Distribution.

*Chthamalus moro* is widely distributed in the Indo-Pacific-Indonesia, Philippines, Taiwan, Xisha Islands, Ryukyu Islands, Palau, Mariana Islands, Caroline Islands, Fiji, and Samoa (Southward and Newman 2003). In this study, *C. moro* was found on the islands of Ambon (at Hatu, Mamala, Alang, Asilulu, Hila, Morella, Wakasihu), Pombo, Seram (at Murnaten), and Saparua (at Ihamahu, Kulur, and Waisisil) on mangroves, stone, port pole, mollusd shell, shells of *Tetraclita squamosa*, *Tesseropora rosea* and *Capitulum mitella* (a map with the occurrence of *Chthamalus moro* in the Moluccas is shown in Suppl. material 1: Fig. S3).

######## Remarks.

Species of the genus *Chthamalus* are very difficult to distinguish in the field. *Chthamalus moro* has a stellate appearance and is smaller than *C. malayensis* (Southward and Newman 2003). In addition, conical spines on the dorsal side of cirrus I are absent and setae on cirrus II are without basal guards in *C. moro*.

#### Superfamily Tetraclitoidea Gruvel, 1905

**Family Tetraclitidae Gruvel, 1903**

**Subfamily Tetraclitellinae Newman & Ross, 1976**

**Genus *Tetraclitella* Hiro, 1939**

**Subgenus Tetraclitella Hiro, 1939**

##### Tetraclitella divisa

Taxon classificationAnimaliaSessiliaTetraclitidae

(Nilsson-Cantell, 1921)

EB86B7C7-4367-5088-8708-6AE3469323CF

**Figure 13.**
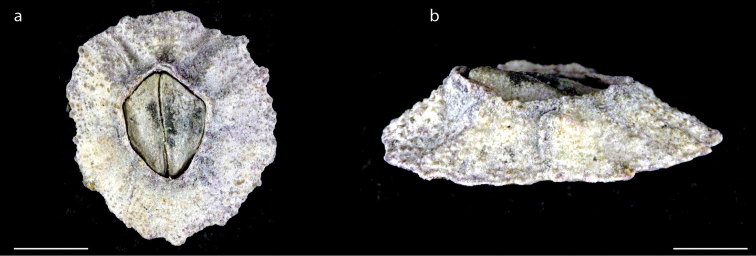
*Tetraclitella divisa* (Nilsson-Cantell, 1921) (MZB Cru Cir 120098) **a** upper view **b** side view. Scale bar: 4 mm.

Figure 13a, b , Table 1: species no. 59

Tetraclita divisa Nilsson-Cantell, 1921: 362, fig. 83, pl. 3 fig. 11.
Tetraclitella (Tetraclitella) divisa : Ross and Perreault 1999: 6.
Tetraclitella divisa : Ross 1968: 13; Dong et al. 1982: 111; Foster 1974: 45, figs 6E–F, 7E- F; Bacon et al. 1984: 86; Paulay and Ross 2003: 308; Chan et al. 2009a: 208, fig. 178.

###### Material examined.

***Ambon Island***: 1 specimen, MZB Cru Cir 120, Laha, 3°43'22.5"S, 128°05'02.5"E, coll. P. Pitriana & D. Tala, 7 Sep 2016.

###### Diagnosis.

Shell with four plates, flattened, not strongly articulated; radii tubiferous; summit of radii horizontal; tergal spur well separated from scutal margin.

###### Description.

Shell depressed, covered by furry chitinous integument; shell plates with prominent radiating ribs; radii wide, porose, tubes running parallel to base of shell; colour of shell pale purplish; orifice diamond shaped (Fig. 13a, b); scutum triangular, tergal margin straight; tergum higher than wide, scutal margin straight, spur short; mandible with four teeth, second and third teeth bidentate; labrum with smooth cutting edge (measurements for one specimen are presented in Suppl. material 1: Table S12).

###### Distribution.

*Tetraclitella divisa* was previously recorded from Western Africa, Java, Malaysia, Sumatra, Northern Australia, Singapore, South China Sea, China, Taiwan, Japan, the Pacific Ocean to Hawaii and Pitcairn (Jones and Hosie 2016). In this study, *T. divisa* was found on Ambon Island at Laha on a concrete wall at the port (a map with the occurrence of *Tetraclitella divisa* in the Moluccas is shown in Suppl. material 1: Fig. S2).

###### Remarks.

*Tetraclita divisa* exhibits a brooded phase to the cypris larval stage in the mantle cavity, whereas most other species release the first stage nauplius (Nilsson-Cantell 1921; Hiro 1939).

##### Tetraclitella karandei

Taxon classificationAnimaliaSessiliaTetraclitidae

Ross, 1971

51B1AA09-ED43-5542-8563-23BA625AEF6E

**Figure 14.**
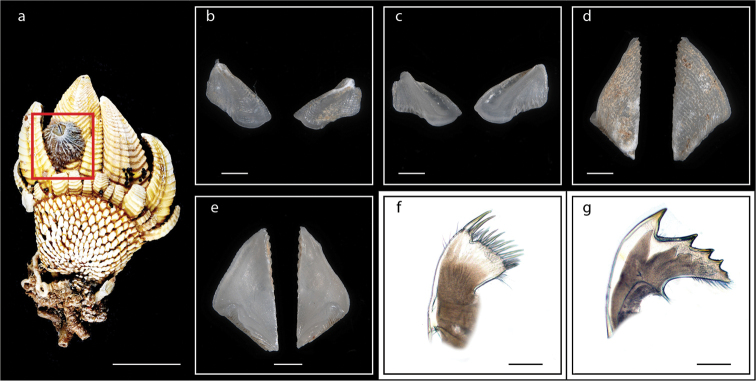
*Tetraclitella karandei* Ross, 1971 (MZB Cru Cir 122-2) on tergum of *Capitulum mitella***a** upper view of *Tetraclitella karandei* on *Capitulum mitella***b** external view of scutum **c** internal view of scutum **d** external view of tergum **e** internal view of tergum **f** maxillule **g** mandible. Scale bars: 15 mm (**a**); 1 mm (**b–e**); 0.25 mm (**f–g**).

Figure 14a–g , Table 1: species no. 60

Tetraclitella (Tetraclitella) karandei : Ross & Perreault, 1999: 6.
Tetraclitella karandei Ross, 1971: 217, figs 2–3, 4A–J; Newmann and Ross 1979: 47; Chan et al. 2009a: 214, fig.184.

###### Material examined.

***Ambon Island***: 10 specimens, MZB Cru Cir 121, Waitatiri, 3°37'04.0"S, 128°16'20.3"E, coll. P. Pitriana & D. Tala, 21 Sep 2017; 2 specimens, MZB Cru Cir 122, Asilulu, 3°40'50.4"S, 127°55'27.6"E, coll. Adin, 20 Sep 2017.

###### Diagnosis.

Shell with four plates, tubiferous, not strongly articulated; radii tubiferous; summit of radii horizontal and elevated above the surface of the parietes; parietes with longitudinal ribs; scutum with nodose ornamentation.

###### Description.

Shell with orifice diamond shaped, colour greyish (Fig. 14a); surface of parietes with chitinous coating and fine hairs, parietes with longitudinal ribs intercalated with lower secondary and tertiary ribs; radii broad, horizontally ridged from base to apex; scutum triangular, occluding and basal margins almost perpendicular, tergal margin straight, surface ornamentation nodose; tergum higher than wide, scutal margin straight, spur small; mandible with four teeth (Fig. 14g), labrum slightly notched, two small teeth on each cutting edge. Basal length 10.1–17.4 mm, basal width 8.2–18.2 mm, height 0.4–0.7 mm. Orifice length 3.8–5.6 mm, orifice width 2.7–5.2 mm (measurements for three specimens are presented in Suppl. material 1: Table S13).

###### Distribution.

*Tetraclitella karandei* was previously recorded from India, Taiwan, the Philippine (Chan et al. 2009a). In this study, *T. karandei* was found on Ambon Island at Waitairi and Asilulu on stone, on the shells of *Capitulum mitella* and *Euraphia hembeli* (a map with the occurrence of *Tetraclitella karandei* in the Moluccas is shown in Suppl. material 1: Fig. S2).

###### Remarks.

*Tetraclitella karandei* can be distinguished by its radii, which are broad and have extended out and over the adjoining plates. The scutum is also unique because it has nodose ornamentation (Ross 1971).

##### Subfamily Tetraclitinae Gruvel, 1903

**Genus *Tesseropora* Pilsbry, 1916**

###### Tesseropora rosea

Taxon classificationAnimaliaSessiliaTetraclitidae

(Krauss, 1848)

6F23A4F5-3949-5F6F-9A1F-21DB64BAA92F

**Figure 15.**
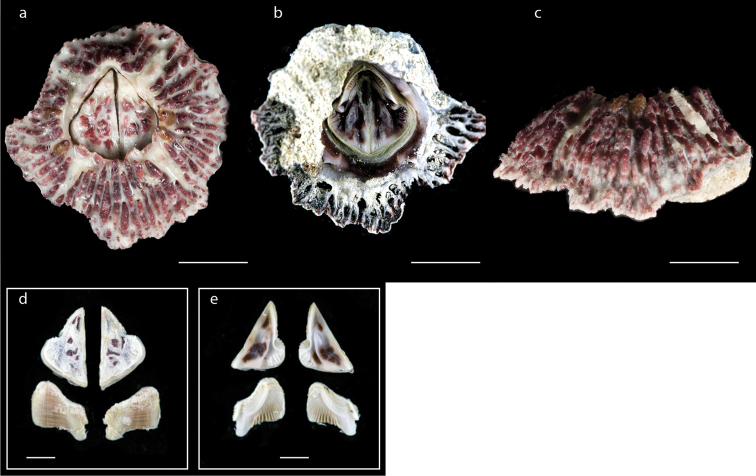
*Tesseropora rosea* (Krauss, 1848) (MZB Cru Cir 077-1) **a** upper view **b** lower view **c** side view **d** external view of scutum and tergum **e** internal view of scutum and tergum. Scale bars: 6 mm (**a–c**); 2 mm (**d, e**).

Figure 15a–e , Table 1: species no. 61

Conia rosea Krauss, 1848: 136.
Tetraclita rosea Darwin, 1854: 335, pl.10 fig. 3a–3d; Pilsbry 1916: 260, pl. 58 fig. 4.
Tesseropora rosea Newman & Ross, 1976: 47; Anderson and Anderson 1985: 89, figs 1–10; Jones and Anderson 1990: 13.

####### Material examined.

***Ambon Island***: 6 specimens, MZB Cru Cir 075, Rutong, 3°42'23.7"S, 128°16'08.9"E, coll. P. Pitriana, 14 Jan 2016; 1 specimen, MZB Cru Cir 076, Leahari, 3°42'45.3"S, 128°16'16.5"E, coll. P. Pitriana, 14 Jan 2016; 25 specimens, MZB Cru Cir 077, Liang, 3°30'13.3"S, 128°20'34.1"E, coll. P. Pitriana & D. Tala, 7 Sept 2016. ***Saparua Island***: 25 specimens, MZB Cru Cir 078, Dermaga Ihamahu, 3°31'13.0"S, 128°41'14.9"E, coll. P. Pitriana & D. Tala, 11 Apr 2016; 4 specimens, MZB Cru Cir 079, Kulur, 3°29'48.5"S, 128°36'10.7"E, coll. P. Pitriana & D. Tala, 20 Sep 2016; 10 specimens, MZB Cru Cir 080, Porto, 3°34'58.2"S, 128°36'58.2"E, coll. P. Pitriana & D. Tala, 20 Sep 2016.

####### GenBank accession number.

COI gene (MK995370).

####### Diagnosis.

Shell with four plates; wall of the parietes with a single row of parietal pore; orifice with traces of pink in colour; oral cone relatively broad; mouthparts relatively large.

####### Description.

Shell steeply conical, whitish tinged pink, with longitudinal purple pinkish striations (Fig. 15a); four parietal plates with single row of large, square tubes, often eroded in upper areas giving pillared appearance (Fig. 15b, c); radii solid, well developed; orifice pentagonal in uneroded specimens, triangular in eroded specimens; basis mostly calcareous; scutum thick, articular furrow short, deep, articular ridge long, adductor ridge prominent, crests for lateral depressor faint; tergum with short, broad spur set close to basiscutal angle, wide articular furrow, carinal depressor crests prominent (Fig. 15d, e); maxillule with two large setae at the lateral angle; mandible with four teeth, labrum shallowly concave in shape, teeth on each side. Basal length 9.7–25.6 mm, basal width 9.7–24.5 mm, height 4.4–13.0 mm. Orifice length 2.9–7.8 mm, orifice width 2.3–6.9 mm (measurements for 15 specimens are presented in Suppl. material 1: Table S14).

####### Distribution.

*Tesseropora rosea* was originally described from a specimen collected at Algoa Bay, South Africa (Krauss 1848; Darwin 1854) and has since been recorded from Australia (SW and SE); Lord Howe Island and the Kermadec Islands (Jones 1990). In this study, *T. rosea* was found on Ambon Island (at Rutong, Leahari, and Liang) and Saparua Island (at Ihamahu, Kulur, and Porto) on stone and mollusc shells (a map with the occurrence of *Tesseroppora rosea* in the Moluccas is shown in Suppl. material 1: Fig. S4).

####### Remarks.

According to Anderson and Anderson (1985), *T. rosea* feeds in different ways, extending the cirral fan only in response to the fast water currents. Thus, *T. rosea* cannot survive in areas with a low current velocity. *Tesseropora rosea* exhibits a wide distribution although the species is represented by relatively few specimens.

###### Genus *Tetraclita* Schumacher, 1817

####### Tetraclita kuroshioensis

Taxon classificationAnimaliaSessiliaTetraclitidae

Chan, Tsang & Chu, 2007

BC22D2A7-2FCB-5130-BF9B-8284060BEFC1

**Figure 16.**
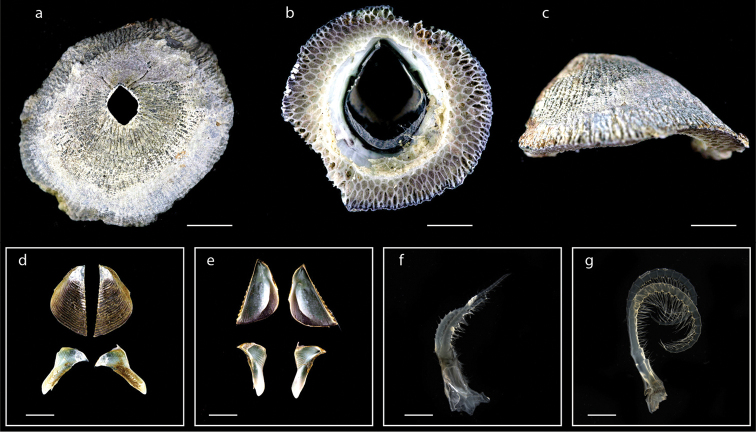
*Tetraclita kuroshioensis* Chan, Tsang & Chu, 2007 (MZB Cru Cir 097) **a** upper view **b** lower view **c** side view **d** external view of scutum and tergum **e** internal view of scutum and tergum **f** cirrus I **g** cirrus VI. Scale bars: 7 mm (**a–c**); 2 mm (**d–e**); 1 mm (**f, g**).

Figure 16a–g , Table 1: species no. 62

Tetraclita squamosa viridis : Hiro 1936b: 635.
Tetraclita squamosa squamosa : Utinomi 1968a: 178.
Tetraclita pacifica Chan et al., 2007a: 88, figs 4–6.
Tetraclita kuroshioensis Chan et al., 2007: 56; Chan et al. 2009a: 192, fig. 164; Pochai et al. 2017: 21, fig. 6.

######## Material examined.

***Ambon Island***: 1 specimen, MZB Cru Cir 097, Hatu, 3°43'52.7"S, 128°02'51.4"E, coll. Adin, 20 Sep 2017; 6 specimens, MZB Cru Cir 098, Ureng, 3°40'14.0"S, 127°56'47.6"E, coll. Adin, 20 Sep 2017. ***Saparua Island***: 1 specimen, MZB Cru Cir 100, Dermaga Ihamahu, 3°31'13.0"S, 128°41'14.9"E, coll. P. Pitriana & D. Tala, 11 Apr 2016.

######## GenBank accession numbers.

COI gene (MK995363, MK995364, MK995367), 18S (MK981375, MK9876, MK981379).

######## Diagnosis.

Shell conical with four plates, tubiferous; radii solid; tergum broad, apex not beaked.

######## Description.

Shell with four inseparable, multi-tubiferous plates, greyish black to purplish-grey or deep green to green, surfaces with mosaic scales pattern radiating randomly from base to apex, internal surface of parietes smooth, white with dark grey striations around aperture; radii solid (Fig. 16a–c); basis membranous; scutum larger than tergum, triangular, external surface with horizontal striations, occluding margin with fine teeth; tergum broad, higher than wide, apex not produced as beak, spur sharp, basi-scutal angle smaller than that of *Tetraclita squamosa* (Fig. 16d, e); external surface of operculum grey and yellowish-light brown, internal surface greyish-dusky green; mandible with four large teeth; maxillule not notched with eleven setae; labrum with five small teeth on each side; cirrus I possessing serrulate setae. Basal length 12.1–21.6 mm, basal width 18.1–21.8 mm, height 7.3–10.4 mm. Orifice length 3.2–5.3 mm, orifice width 2.4–4.2 mm (measurements for five specimens are presented in Suppl. material 1: Table S15).

######## Distribution.

*Tetraclita kuroshioensis* was previously recorded from Japan, Taiwan, Palau, and Thailand (Chan et al. 2009a; Pochai et al. 2017). In this study, *T. kuroshioensis* was found on Ambon Island (at Hatu and Ureng) and Saparua Island (at Dermaga Ihamahu) on rocks and concrete wall of a port (a map with the occurrence of *Tetraclita kuroshioensis* in the Moluccas is shown in Suppl. material 1: Fig. S4).

######## Remarks.

*Tetraclita kuroshioensis* and *T. squamosa* share great morphological similarity. However, DNA sequences separate the two species (Chan et al. 2007), which was confirmed in this study (Fig. 29). Morphologically, the shape of the tergum is definitive; that of *T. kuroshioensis* is broader and the apex blunter compared to *T. squamosa* (Chan et al. 2007).

####### Tetraclita squamosa

Taxon classificationAnimaliaSessiliaTetraclitidae

(Bruguiére, 1789)

41906792-EE3B-5FDB-AC4D-906321DE67C5

**Figure 17.**
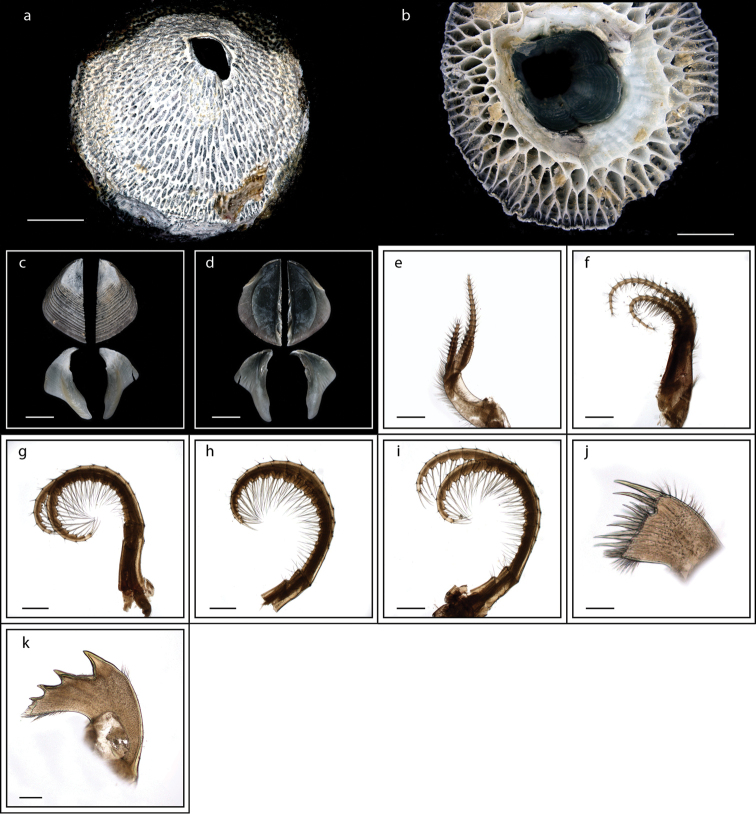
*Tetraclita squamosa* (Bruguiére, 1789) (MZB Cru Cir 081-3) **a** upper view **b** lower view **c** external view of scutum and tergum **d** internal view of scutum and tergum **e** cirrus I **f** cirrus II **g** cirrus III **h** cirrus IV **i** cirrus VI **k** maxillule **l** mandible. Scale bars: 8 mm (**a, b**); 2 mm (**c, d**); 1 mm (**e, f**); 2 mm (**g–i**); 0.5 mm (**j, k**).

Figure 17a–k , Table 1: species no. 63

Balanus squamosus Bruguière, 1789: 170, pl. 165 figs 9, 10.
Lepas fungites Spengler, 1790: 189.
Lepas porosa Gmelin, 1791: 3212; Wood 1815: pl. 9 fig. 4.
Tetraclita squamulosa Schumacher, 1817: 91.
Asemus porosus : Ranzani 1820: pl. 3 figs 32–35.
Conia porosa : Sowerby 1823: pl. 1.
Tetraclita porosa var. (3) viridis Darwin, 1854a: 329.
Tetraclita porosa viridis : Nilsson-Cantell 1921: 364.
Tetraclita squamosa squamosa : Pilsbry 1916: 251; Dong et al. 1982: 110, fig.
Tetraclita squamosa forma viridis : Broch 1922: 337.
Tetraclita squamosa viridis : Hiro 1936b: 635.
Tetraclita porosa perfecta Nilsson-Cantell, 1931a: 133, pl. II fig. 8a–e.
Tetraclita squamosa : Stebbing 1910: 570; Ren and Liu 1979: 339, pl. 1 figs 1–11; Yamaguchi 1987: 344; Zevina et al. 1992: 45, fig. 30; Chan 2001: 625, fig. 8; Chan et al. 2007a: 82, fig. 4; Chan et al. 2009a: 195, fig. 167; Pochai et al. 2017: 25, fig.8.

######## Material examined.

***Ambon Island***: 17 specimens, MZB Cru Cir 081, Alang, 3°45'11.0"S, 128°01'23.1"E, coll. Adin, 20 Sep 2017; 15 specimens, MZB Cru Cir 082, Dermaga Liang, 3°30'13.3"S, 128°20'34.1"E, coll. P. Pitriana & D. Tala, 30 Aug 2016; 2 specimens, MZB Cru Cir 083, Dermaga Tulehu, 3°35'21.8"S, 128°20'02.8"E, coll. P. Pitriana & D. Tala, 7 Sep 2016; 15 specimens, MZB Cru Cir 084, Doc Tawiri, 3°42'10.1"S, 128°06'13.4"E, coll. P. Pitriana & D. Tala, 29 Mar 2016; 2 specimens, MZB Cru Cir 085, Gudang Arang, 3°42'07.2"S, 128°09'43.7"E, coll. P. Pitriana & D. Tala, 5 Sep 2016; 5 specimens, MZB Cru Cir 086, Hila, 3°34'57.5"S, 128°05'31.9"E, coll. Adin, 20 Sep 2017; 17 specimens, MZB Cru Cir 087, Hutumuri, 3°41'47.6"S, 128°17'44.1"E, coll. P. Pitriana, 14 Jan 2016; 5 specimens, MZB Cru Cir 088, Leahari, 3°42'45.3"S, 128°16'16.5"E, coll. P. Pitriana, 14 Jan 2016; 6 specimens, MZB Cru Cir 089, Tawiri, 3°42'10.1"S, 128°06'13.4"E, coll. P. Pitriana & D. Tala, 5 Sep 2016; 3 specimens, MZB Cru Cir 090, Tulehu, 3°35'21.8"S, 128°20'02.8"E, coll. P. Pitriana & D. Tala, 7 Sep 2016; 7 specimens, MZB Cru Cir 091, Waai, 3°33'23.5"S, 128°19'33.9"E, coll. P. Pitriana & D. Tala, 7 Sep 2016; 2 specimens, MZB Cru Cir 092, Waai, 3°33'23.5"S, 128°19'33.9"E, coll. P. Pitriana & D. Tala, 31 Mar 2017; 45 specimens, MZB Cru Cir 096, Asilulu, 3°40'50.4"S, 127°55'27.6"E, coll. Adin, 20 Sep 2017; 40 specimens, MZB Cru Cir 099, Wakasihu, 3°46'27.6"S, 127°56'36.6"E, coll. Adin, 20 Sep 2017. ***Saparua Island***: 5 specimens, MZB Cru Cir 093, Benteng Durstede, 3°34'32.8"S, 128°39'34.7"E, coll. P. Pitriana & D. Tala, 8 Apr 2016; 6 specimens, MZB Cru Cir 094, Teluk Saparua, 3°34'25.7"S, 128°39'25.8"E, coll. P. Pitriana & D. Tala, 8 Apr 2016; 5 specimens, MZB Cru Cir 095, Teluk Saparua, 3°34'25.7"S, 128°39'25.8"E, coll. P. Pitriana & D. Tala, 22 Sept 2016.

######## GenBank accession numbers.

COI gene (MK995360–MK995362), 18S (MK981368–MK981373).

######## Diagnosis.

Shell conical with four plates, tubiferous; radii solid; tergum narrow, concaved, apex beaked.

######## Description.

Shell consisting of four fused, inseparable plates (Fig. 17a), parietes with eight rows of multi-tubiferous parietal tubes (Fig. 17b), external surface with longitudinal lines from base to apex, internal surface smooth, white with purplish grey striations close to aperture; orifice rhomboidal; basis membranous; shell greenish with brownish grey (Fig. 17a), external surface of operculum brownish grey, internal surface purplish grey; scutum triangular, larger than tergum, external surface with horizontal striations, occluding margin with very shallow teeth; tergum narrow, apex beaked, spur long, sharp (Fig. 17c, d); mandible with four large teeth, first tooth with three small spines, lower margin pectinate (Fig. 17k); maxillule notched with two large setae above notch, elevan small setae below notch (Fig. 17j); labrum with four large teeth on each side of notch; cirrus I with bidentate serrulate setae (Fig. 17e). Basal length 20.1–30.2 mm, basal width 19.3–28.3 mm, height 11.9–14.2 mm. Orifice length 4.4–7.9 mm, orifice width 3.4–7.2 mm (measurements for five specimens are presented in Suppl. material 1: Table S16).

######## Distribution.

*Tetraclita squamosa* is widespread in the Indo-Pacific region, Australia, South China coast, and Taiwan (Newman 1978; Jones et al. 2001; Chan et al. 2009a). In this study, *T. squamosa* was found on Ambon Island (at Alang, Dermaga Liang, Dermaga Tulehu, Doc Tawiri, Gudang Arang, Hila, Hutumuri, Leahari, Tawiri, Tulehu, Waai, Asilulu, Wakasihu) and Saparua Island (at Benteng Duurstede and Teluk Saparua) on stone, rocks, shipyards, concrete bridges and walls of the port (a map with the occurrence of *Tetraclita squamosa* in the Moluccas is shown in Suppl. material 1: Fig. S4).

######## Remarks.

*Tetraclita squamosa* has characteristic green parietes (Yamaguchi 1987) and a wide distribution throughout the Indo-Pacific (Newman and Ross 1976). However, the taxonomy of *Tetraclita squamosa* has been confusing due to a high degree of morphological variation, and it is now considered a species complex. *Tetraclita squamosa* and *Tetraclita japonica* can be separated using characters such as the shape of the parietes, scutum geometry, and mandible structures (Darwin 1854; Pilsbry 1916). In addition, a key character for *T. squamosa* is the tergum with a beak on its apex (Chan et al. 2009a).

##### Subfamily Newmanellinae Ross & Perreault, 1999

**Genus *Yamaguchiella* Ross & Perreault, 1999**

###### Yamaguchiella coerulescens

Taxon classificationAnimaliaSessiliaTetraclitidae

(Spengler, 1790)

FD824242-7D46-5EA8-AFCC-B14B575B5D7B

**Figure 18.**
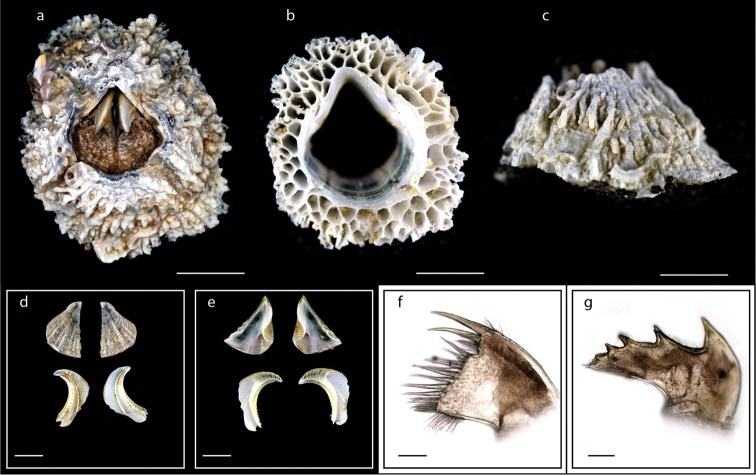
*Yamaguchiella coerulescens* (Spengler, 1790) (MZB Cru Cir 123-2) **a** Upper view **b** lower view **c** side view **d** external view of scutum and tergum **e** internal view of scutum and tergum **f** maxillule **g** mandible. Scale bars: 7 mm (**a–c**); 1 mm (**d, e**); 0.5 mm (**f, g**).

Figure 18a–g , Table 1: species no. 64

Lepas coerulescens Spengler, 1790: 191.
Tetraclita coerulescens : Darwin 1854: 342, pl. 11 figs 4a–d; Hoek 1883: 161, pl. 13 fig. 34; Pilsbry 1916: 259; Nilsson-Cantell 1938: 77; Newman and Ross 1976: 47; Dong et al. 1982: 111; Zevina et al. 1992: 48, fig. 31.
Yamaguchiella (Yamaguchiella) coerulescens : Ross and Perreault 1999: 5; Jones and Hosie 2016: 271; Chan et al. 2009a: 202, fig. 173.

####### Material examined.

***Ambon Island***: 13 specimens, MZB Cru Cir 123, Gudang Arang, 3°42'07.2"S, 128°09'43.7"E, coll. P. Pitriana & D. Tala, 5 Sep 2016; 4 specimens, MZB Cru Cir 124, Dermaga Tulehu, 3°35'05.4"S, 128°19'43.3"E, coll. P. Pitriana & D. Tala, 7 Sep 2016; 5 specimens, MZB Cru Cir 125, Tulehu, 3°35'21.8"S, 128°20'02.8"E, coll. Adin, 19 Sep 2017; 14 specimens, MZB Cru Cir 126, Doc Tawiri, 3°42'10.1"S, 128°06'13.4"E, coll. P. Pitriana & D. Tala, 29 Mar 2016; 8 specimens, MZB Cru Cir 127, Tawiri, 3°42'10.1"S, 128°06'13.4"E, coll. P. Pitriana & D. Tala, 5 Sep 2016; 2 specimens, MZB Cru Cir 128, Galala, 3°41'22.2"S, 128°10'52.6"E, coll. P. Pitriana & D. Tala, 6 Sep 2016; 11 specimens, MZB Cru Cir 129, Waai, 3°33'23.5"S,128°19'33.9"E, coll. P. Pitriana & D. Tala, 7 Sep 2016; 4 specimens, MZB Cru Cir 130, Pelabuhan Yos Sudarso, 3°41'36.5"S, 128°10'35.6"E, coll. P. Pitriana & D. Tala, 6 Sep 2016. ***Saparua Island***: 1 specimen, MZB Cru Cir 131, Kulur, 3°29'48.5"S, 128°36'10.7"E, coll. P. Pitriana & D. Tala, 20 Sep 2016.

####### GenBank accession number.

18S (MK981381).

####### Diagnosis.

Shell with the upper part tinged greenish-blue, longitudinally ribbed; radii moderately wide, with their summits oblique; scutum with a small adductor and extremely prominent articular ridge, united together and forms a small sub-cylindrical cavity; tergum with the spur not joined to the basi-scutal angle.

####### Description.

Shell low conical to cylindro-conic (Fig. 18c) with four multi-tubiferous plates (Fig. 18b), parieties greenish or greyish with radiating lines (Fig. 18a); radii wide, summits oblique; basis calcareous, radii wide, tubiferous, summits oblique; orifice occluded wholly by scutum (Fig. 18a); scutum and tergum strongly articulated (Figs 18d, e); mandible with four teeth, the third teeth is tridentate (Fig. 18g); labrum with three large teeth on each side of cutting edge; penis with basidorsal point. Basal length 8.3–29.0 mm, basal width 8.5–27.8 mm, height 5.7–17.7 mm. Orifice length 4.2–11.5 mm, orifice width 3.2–11.8 mm (measurements for 25 specimens are presented in Suppl. material 1: Table S17).

####### Distribution.

*Yamaguchiella coerulescens* was previously recorded from the Indo-west Pacific: the Indian Ocean, Bay of Bengal, Mergui Archipelago, Kei Islands, Banda Island, Malay Archipelago, Sulu Archipelago, Vietnam, China, Philippines, Goram Island, Palao Island, and Taiwan (Jones and Hosie 2016). In this study, *Y. coerulescens* was found on Ambon Island (at Tulehu, Dermaga Gudang Arang, Waai, Doc. Tawiri, Liang, Galala, Dermaga Yos Sudarso) and Saparua Island (at Kulur) on stone (a map with the occurrence of *Yamaguchiella coerulescens* in the Moluccas is shown in Suppl. material 1: Fig. S6).

####### Remarks.

The subgenus Yamaguchiella was proposed by Ross and Perreault (1999) in honour of Toshiyuki Yamaguchi (Chiba University Japan), in appreciation of his contributions to the knowledge of recent and fossil barnacles.

###### Genus *Yamaguchiella* Ross & Perreault, 1999

**Subgenus Neonrosella Jones, 2010**

####### Neonrosella vitiata

Taxon classificationAnimaliaSessiliaTetraclitidae

(Darwin, 1854)

E02299A5-B940-56B8-A21E-5248F000CA9A

**Figure 19.**
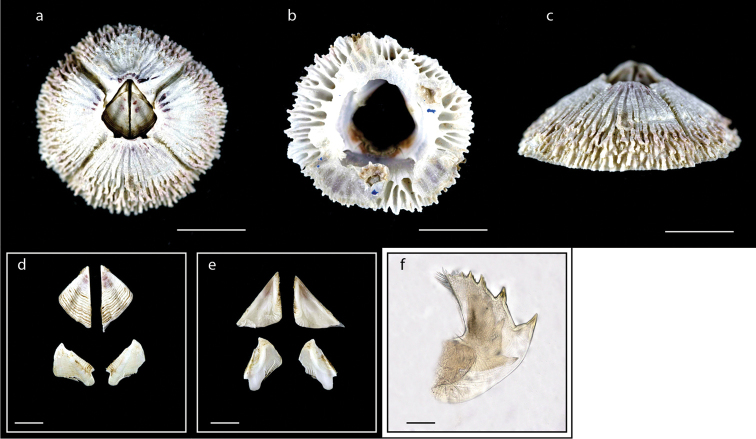
*Neonrosella vitiata* (Darwin, 1854) (MZB Cru Cir 132-3) **a** upper view **b** lower view **c** side view **d** external view of scutum and tergum **e** internal view of scutum and tergum **f** mandible. Scale bars: 8 mm (**a–c**); 1 mm (**d, e**); 0.5 mm (**f**).

Figure 19a–f , Table 1: species no. 65

Tetraclita vitiata Darwin, 1854: 340, pl. 11 fig. 3a–e.
Tetraclita (Tetraclita) vitiata : Rosell 1972: 214.
Newmanella vitiata : Ikeya and Yamaguchi 1993: 93; Jones et al. 1990: 14.
Yamaguchiella (Rosella) vitiata : Ross & Perreault, 1999: 5.
Yamaguchiella (Neonrosella) vitiata : Jones 2010: 214.
Neonrosella vitiata : Sukparangsi et al. 2019:4, figs 1–4.

######## Material examined.

***Ambon Island***: 3 specimens, MZB Cru Cir 132, Liang, 3°30'13.3"S, 128°20'34.1"E, coll. P. Pitriana & D. Tala, 7 Sep 2016. ***Banda Neira Island***: 1 specimen, MZB Cru Cir 133, Banda Neira, 4°31'22.8"S, 129°53'52.5"E, coll. P. Pitriana, 25 May 2016. ***Saparua Island***: 4 specimens, MZB Cru Cir 134, Tuhaha, 3°32'38.1"S, 128°40'58.0"E, coll. P. Pitriana & D. Tala, 21 Sep 2016.

######## GenBank accession number.

18S (MK981384).

######## Diagnosis.

Parietes low with wall spreading; peritreme slightly toothed; base calcareous with two rows of irregular shape and size of parietal tubes; tergum with broad spur; lateral scutal depressor crests numerous and deep; five toothed mandibles; segments of posterior cirri with four pairs spines.

######## Synoptic description.

Shell four plated, conical, whitish with spots of purple in upper part (Fig. 19a); parietal tubes irregular shape, size unequal (Fig. 19b); radii moderately wide, summits oblique, interior of irregularly branching ridges with solid interspaces (Fig. 19c); orifice trigonal; scutum and tergum coalesced, strongly articulated (Fig. 19d, e); tergum with broad spur, lateral tergal depressor crests on basal margin long, with numerous, deep crests, lateral scutal depressor crests numerous, deep; mandible with five teeth (Fig. 19f). Basal length 15.4–22.8 mm, basal width 13.8–22.8 mm, height 5.3–6.9 mm. Orifice length 5.2–6.9 mm, orifice width 4.6–5.4 mm (measurements for four specimens are presented in Suppl. material 1: Table S18).

######## Distribution.

*Neonrosella vitiata* was previously recorded from the Indo-west Pacific, Indian Ocean, Nicobar Island to Australia, Indonesia, Malay Archipelago, Sulu Archipelago, Philippines, and the Pacific Ocean (Jones and Hosie 2016). Recently, *Neonrosella vitiata* also was discovered in the Andaman Sea of Thailand (Sukparangsi et al. 2019). In this study, *N. vitiata* was found on Ambon Island (at Liang), Banda Island and Saparua Island (at Tuhaha) on port poles, reef and stones (a map with the occurrence of *Neonrosella vitiata* in the Moluccas is shown in Suppl. material 1: Fig. S7).

######## Remarks.

*Neonrosella vitiata* can be distinguished by its irregular parietal tubes, the shape of the terga, the five toothed mandibles and four pairs of spines on the segments of the posterior cirri (Darwin 1854).

###### Genus *Newmanella* Ross, 1969

####### Newmanella spinosus

Taxon classificationAnimaliaSessiliaTetraclitidae

Chan & Cheang, 2016

ED1AB140-28C2-5557-B8F2-0A795EABE7E7

**Figure 20.**
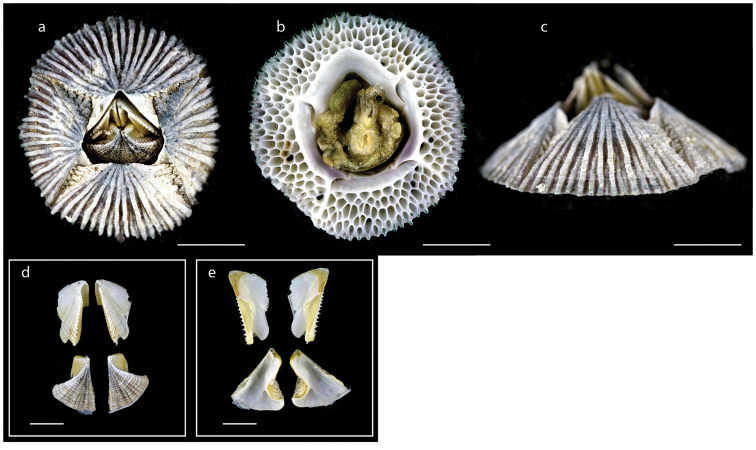
*Newmanella spinosus* Chan & Cheang, 2016 (MZB Cru Cir 072-1) **a** upper view **b** lower view **c** side view **d** external view of scutum and tergum **e** internal view of scutum and tergum. Scale bars: 7 mm (**a–c**); 2 mm (**d, e**).

Figure 20a–e , Table 1: species no. 66

Newmanella radiata : Chan et al. 2009: 199, fig. 170.
Newmanella sp. Tsang et al., 2015: 325, fig. 1A; 327 fig. 2.
Newmanella spinosus Chan & Cheang, 2016: 212, figs 9–15; Pochai et al. 2017: 20, fig. 5; Sukparangsi et al. 2019: 10, figs 5–8.

######## Material examined.

***Ambon Island***: 5 specimens, MZB Cru Cir 072, Rutong, 3°42'23.7"S ,128°16'08.9"E, coll. P. Pitriana, 14 Jan 2016.

######## Diagnosis.

Shell low conical to cylindro-conical; parietes discrete; base calcareous; radii broad; scutum with very deep depressor muscle crests; cirrus II and cirrus IV having numerous triangular spines; fourth and fifth teeth of mandible separated; cutting edge of maxillule below notch protruding; intromittent organ of penis lacking basi-dorsal point.

######## Description.

Shell low conical, four plates externally greyish in colour, parietes with deep longitudinal, radiating lines from base to apex, internally with multiple rows of irregular parietal tubes (Fig. 20a, b); radii wide with horizontal striations, summits oblique (Fig. 20c); scutum triangular, external surface with horizontal striations, adductor ridge conspicuous; tergum high, narrow, basal margin with well-developed depressor muscle crests projecting beyond border; orifice pentagonal (Fig. 20d, e); basis calcareous, tubiferous, tubes in single layer; mandible with five teeth, the first tooth is the largest and separated from the rest, while the fifth tooth is the smallest and located at the middle of lower margin; labrum with V-shaped notch, two large teeth on the right side, five teeth on the left side of cutting margin; penis without basidorsal point, with few bundles of setae distally. Basal length 17.4–20.9 mm, basal width 15.9–20.5 mm, height 6.8–8.9 mm. Orifice length 5.3–7.3 mm, orifice width 5.0–6.7 mm (measurements for five specimens are presented in Suppl. material 1: Table S19).

######## Distribution.

*Newmanella spinosus* was previously recorded from Japan, Taiwan, Philippines, and Thailand (Chan and Cheang 2016; Pochai et al. 2017). In the current study, this range is extended to Rutong (on stones and reef surface), Ambon Island (a map with the occurrence of *Newmanella spinosus* in the Moluccas is shown in Suppl. material 1: Fig. S2).

######## Remarks.

*Newmanella spinosus* is morphologically close to *Newmanella radiata* but it can be distinguished by the morphology of the scutum, tergum, cirrus II, mandible and maxillule. *N. spinosus* also has numerous spines on its cirri, especially on cirrus II, which is different from *N. radiata* (Chan and Cheang 2016).

##### Family Balanidae Leach, 1817

**Subfamily Amphibalaninae Pitombo, 2004**

**Genus *Amphibalanus* Pitombo, 2004**

###### Amphibalanus amphitrite

Taxon classificationAnimaliaSessiliaBalanidae

(Darwin, 1854)

B10A3AF7-B31F-59B6-8FC4-49E4BA2F8E83

**Figure 21.**
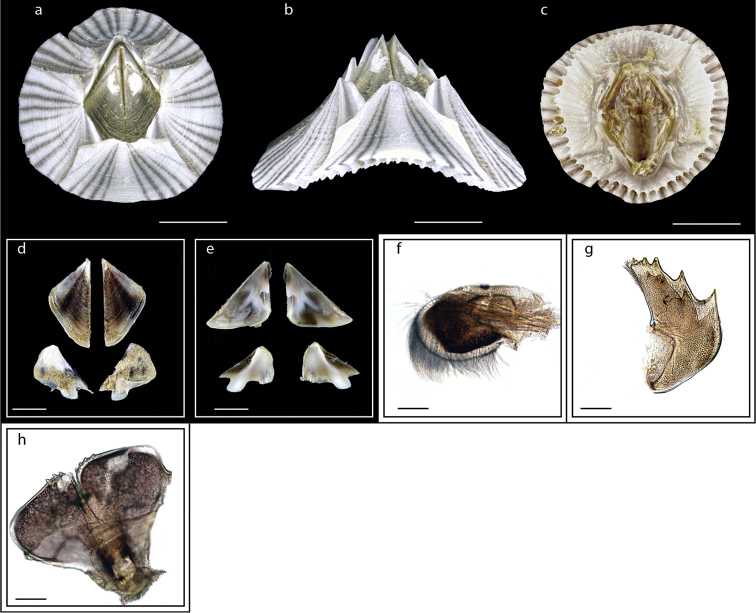
*Amphibalanus amphitrite* (Darwin, 1854) (MZB Cru Cir 005-4) **a** upper view **b** side view **c** lower view **d** external view of scutum and tergum **e** internal view of scutum and tergum **f** maxilla **g** mandible **h** labrum. Scale bars: 4 mm (**a–c**); 1 mm (**d, e**); 0.5 mm (**f–h**).

Figure 21a–h , Table 1: species no. 87

Balanus amphitrite Darwin, 1854: 240 (part.), pl. 5. figs 2a–d, i–k, m–o; Weltner 1897:264; Hoek 1913: 167; Pilsbry 1916: 89; Zevina et al. 1992: 89, fig. 61; Puspasari et al. 2001b: 7.
Balanus amphitrite var. (1) communis Darwin, 1854: 240, pl. 5 fig. 2e, h, l.
Balanus amphitrite communis : Nilsson-Cantell 1921: 311, fig. 64.
Balanus amphitrite forma hawaiiensis Broch, 1922: 314, fig. 56 (part.).
Balanus amphitrite forma denticulata Broch, 1927b: 133, fig. 14 (part.).
Balanus amphitrite hawaiiensis : Hiro 1937c: 432, figs 20, 21.
Balanus amphitrite cochinensis Nilsson-Cantell, 1938b: 43, fig. 11a–e.
Balanus amphitrite var. fluminensis Oliveira, 1941: 21, pl. 4 fig. 4, pl. 5 figs 1, 2, pl. 8 figs 1–5.
Balanus amphitrite var. aeratus Oliveira, 1941: 22, pl. 4 fig. 5, pl. 9 figs 1–4.
Balanus amphitrite herzi Rogers, 1949: 8, pl. 1 figs 6, 12–15.
Balanus amphitrite franciscanus Rogers, 1949: 9, pl. 1 figs 5, 7, 16–19.
Balanus amphitrite var. columnarius Tarasov & Zevina, 1957: 179, 184, fig. 68 a–e.
Balanus amphitrite denticulata Henry, 1959: 192, pl. 1 fig. 5, pl. 3 fig. 7, upper row right.
Balanus amphitrite amphitrite : Harding 1962: 274, pl. 1a–g, pl. 2a–k; Dong et al. 1982: 90, fig. A–E; Rosell 1981: 302.
Balanus amphitrite var. hawaiiensis : Stubbings 1963b: 15.
Amphibalanus amphitrite : Pitombo 2004: 263, 274, figs 2A, B, 7A, B, 8C; Chan et al. 2009a: 241; Chen et al. 2014: 1071; Shahdadi et al. 2014: 213; Pochai et al. 2017: 27, fig. 9; Xu 2017: 48.

####### Material examined.

***Ambon Island***: 4 specimens, MZB Cru Cir 005, Galala, 3°41'22.2"S ,128°10'52.6"E, coll. P. Pitriana & D. Tala, 6 Sep 2016. ***Saparua Island***: 4 specimens, MZB Cru Cir 007, Desa Mahu, 3°32'19.6"S, 128°41'17.3"E, coll. P. Pitriana & D. Tala, 11 Apr 2016; 5 specimens, MZB Cru Cir 008, Negeri Mahu, 3°31'52.9"S, 128°41'12.4"E, coll. P. Pitriana & D. Tala, 11 Apr 2016; 2 specimens, MZB Cru Cir 009, Tuhaha, 3°32'38.1"S, 128°40'58.0"E, coll. P. Pitriana & D. Tala, 21 Sep 2016.

####### Diagnosis.

Primary parietal tubes with transverse septa; exterior of shell with longitudinal purple striations, horizontal striations absent; tergum short with wide spur; cirri III–VI with erect teeth below posterior angles of distal; cirrus III without complex setae.

####### Description.

Shell six plated, conical, round;, externally smooth, white with groups of well-spaced, dark purple vertical stripes, horizontal striations on shell surface absent (Fig. 21a, b), interior of parietes with single row of tubes (Fig. 21c); radii solid, wide; alae with summits moderately oblique (Fig. 21a, b); basis porous, calcareous; scutum externally striped, internally with prominent articular ridge 3/5 length of tergal margin, well separated from straight adductor ridge, occluding margin toothed, lateral depressor muscle pit small (Fig. 21d, e); tergum with spur wider than long, less than its own width from basi-scutal angle (Fig. 21d, e); mandible with four teeth (Fig. 21g); labrum multi-denticulate (Fig. 21h). Basal length 3.1–17.8 mm; basal width 2.8–17.6 mm; height 2.1–10.8 mm; orifice length 1.5–8.1 mm; orifice width 1.5–5.4 mm (measurements for 15 specimens are presented in Suppl. material 1: Table S20).

####### Distribution.

*Amphibalanus amphitrite* is commonly found on beaches and in estuaries, lives attached to harsh natural substrate, such as bedrock, rocks, shells of molluscs, as well as the roots and trunks of mangrove trees. Many specimens also stick to artificial substrates, such as ship hulls and the walls and pillars of docks. *Amphibalanus amphitrite* is spread globally tropical and subtropical waters (Zullo et al. 1972; Henry and McLaughlin 1975; Chen et al. 2014). In this study, *A. amphitrite* was found on the islands of Ambon (at Galala) and Saparua (at Desa Mahu, Negeri Mahu and Tuhaha) on stone, mollusc shells and the capitulum of *Lepas anserifera* (a map with the occurrence of *Amphibalanus amphitrite* in the Moluccas is shown in Suppl. material 1: Fig. S4).

####### Remarks.

*Amphibalanus amphitrite* is difficult to distinguish from two other members of the subgenus Balanus, i.e., *Balanus crenatus* Bruguière, 1789 and *Balanus trigonus* Darwin, 1854. However, *A. amphitrite* can usually be distinguished from the other species by the multi-denticulated labrum and also by the colour pattern of the parietes and sheath (Henry and McLaughlin 1975).

###### Amphibalanus reticulatus

Taxon classificationAnimaliaSessiliaBalanidae

(Utinomi, 1967)

5E2CFDB6-EF75-5D2E-9793-B592E2A770DC

**Figure 22.**
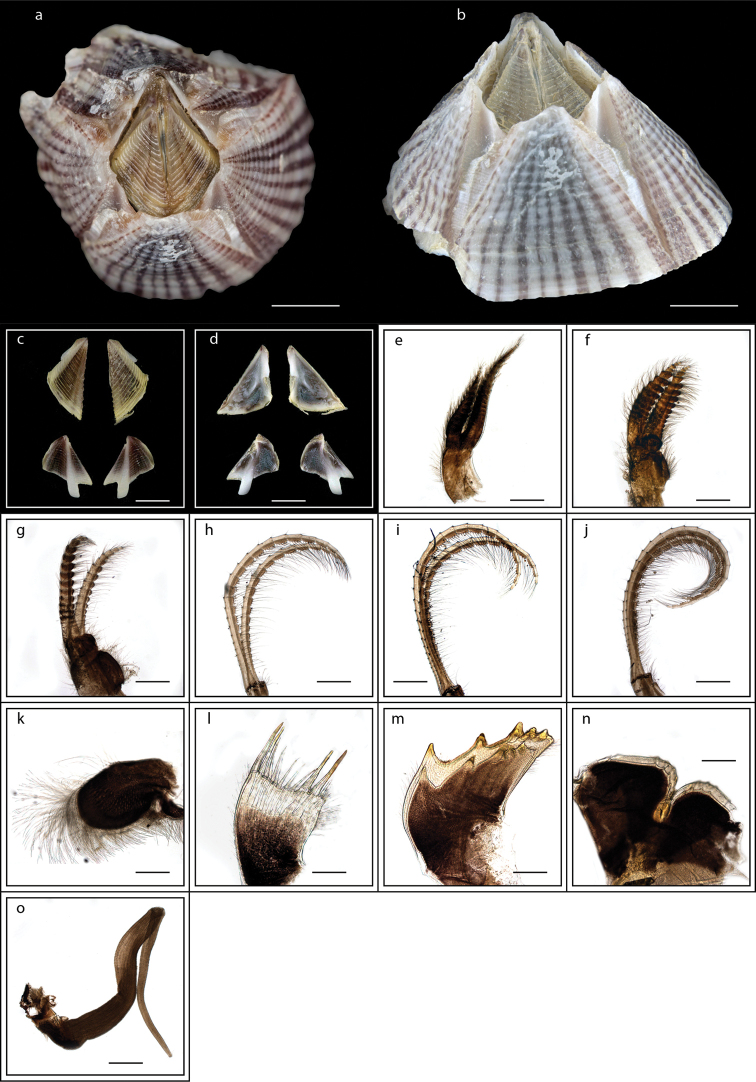
*Amphibalanus reticulatus* (Utinomi, 1967) (MZB Cru Cir 012-1) **a** upper view **b** side view **c** external view of scutum and tergum **d** internal view of scutum and tergum **e** cirrus I **f** cirrus II **g** cirrus III **h** cirrus IV **i** cirrus V **j** cirrus VI **k** maxilla **l** maxillule **m** mandible **n** labrum **o** penis. Scale bars: 4 mm (**a, b**); 1 mm (**c–g**); 2 mm (**h–j**); 2 mm (**o**); 0.5 mm (**k–n**).

Figure 22a–o , Table 1: species no. 88

Balanus amphitrite var. (1) communis Darwin, 1854: 240, pl. 5 fig. 2e, h, l (part.).
Balanus amphitrite forma communis : Broch 1922: 314 (part.).
Balanus amphitrite forma hawaiiensis Broch, 1922: 314 (part.).
Balanus amphitrite communis : Hiro 1938a: 301, fig. 1a, b.
Balanus amphitrite cirratus : Zevina and Tarasov 1963: 89, fig. 10a–e.
Balanus amphitrite var. variegatus : Stubbings 1963a: 329, fig. 2a–e.
Balanus amphitrite variety: Southward and Crisp 1963: 43, fig. 23.
Balanus amphitrite tesselatus Utinomi, 1964: 52, pl. 26 fig. 11.
Balanus amphitrite var. denticulata : Karande & Palekar, 1966: 145, fig. 7, pl.1 fig. 7, pl. 4 row 5 (part.).
Balanus variegatus tesselatus Utinomi & Kikuchi, 1966: 5.
Balanus amphitrite amphitrite : Stubbings 1967: 271, fig. 14d–f (part.).
Balanus reticulatus : Utinomi 1967: 216, figs 9a, b, 10a, b, 11a–e, pl. 6 figs 7, 8 (part.); Dong et al. 1982: 91, fig. A–C; Zevina et al. 1992: 92, fig. 63; Puspasari et al. 2001b.
Amphibalanus reticulatus : Pitombo 2004: 274; Chan, et al. 2009a: 234, fig. 200; Pochai et al. 2017: 26, fig. 10; Xu 2017: 43, figs 10, 39.

####### Material examined.

***Ambon Island***: 5 specimens, MZB Cru Cir 012, Yos Sudarso, 3°41'36.5"S, 128°10'35.6"E, coll. P. Pitriana & D. Tala, 6 Sep 2016.

####### Diagnosis.

Primary parietal tubes with transverse septa; exterior of shell with longitudinal and horizontal striations; anterior margin of cirrus III with conical denticles, erect hooks below posterior angles of distal articles of rami present.

####### Description.

Shell conic or cylindric; six parietal plates, externally smooth, white with groups of well-spaced purple, or purple-pink vertical stripes intersecting with transverse striations (Fig. 22a, b); parietes with single row of internal tubes; alae with summits moderately oblique; radii narrow, summits oblique; orifice toothed; basis calcareous, porous; scutum triangular, externally flat, internally adductor ridge conspicuous, short, low, well separated from prominent articular ridge; tergum flat, spur narrow, short, basiscutal angle acute, scutal margin straight; scutum triangular, occluding margin toothed; tergum flat, basiscutal angle acute, scutal margin straight (Fig. 22c, d); cirrus II with simple spinules or conic teeth on outer face near anterior margin (Fig. 22f); maxilla bilobed, dense setae on margin (Fig. 22k); maxillule not notched, setae on upper and lower margins (Fig. 22l); mandible with four teeth (Fig. 22m); mandibular palp with setulae on lower margin, pinnate setae on upper margin; labrum simple with four teeth and setulae on crest on each side of deep notch (Fig. 22n). Basal length 7.7–16.3 mm; basal width 2.9–15.5 mm; height 3.2–10.5 mm. Orifice length 3.5–8.9 mm; orifice width 2.5–7.1 mm (measurements for five specimens are presented in Suppl. material 1: Table S21).

####### Distribution.

*Amphibalanus reticulatus* is native to the Indo-Pacific region and has been introduced by shipping to tropical-subtropical waters of the Eastern Pacific (Coles et al. 1999; Carlton et al. 2011). *A. reticulatus* can be found from Japan to the Malay Archipelago, east Asia from the Yellow Sea to Gulf of Siam, from Malaysia to southeast Africa, in the Mediterranean Sea, West Africa, the Southeast United States to the West Indies (Henry and McLaughlin 1975).

In this study, *A. reticulatus* was found on the islands of Ambon (at the port of Yos Sudarso) on stone and concrete wall of the port (a map with the occurrence of *Amphibalanus reticulatus* in the Moluccas is shown in Suppl. material 1: Fig. S2).

####### Remarks.

*Amphibalanus reticulatus* can be confused with *A. amphitrite*. However, the shell of *A. reticulatus* exhibits clear vertical and horizontal striations, whilst *A. amphitrite* shows only vertical purple striations on all shell plates (Henry and McLaughlin 1975).

###### Amphibalanus variegatus

Taxon classificationAnimaliaSessiliaBalanidae

(Darwin, 1854)

178A5399-B369-5D3E-8563-F03BC9FD6D3F

**Figure 23.**
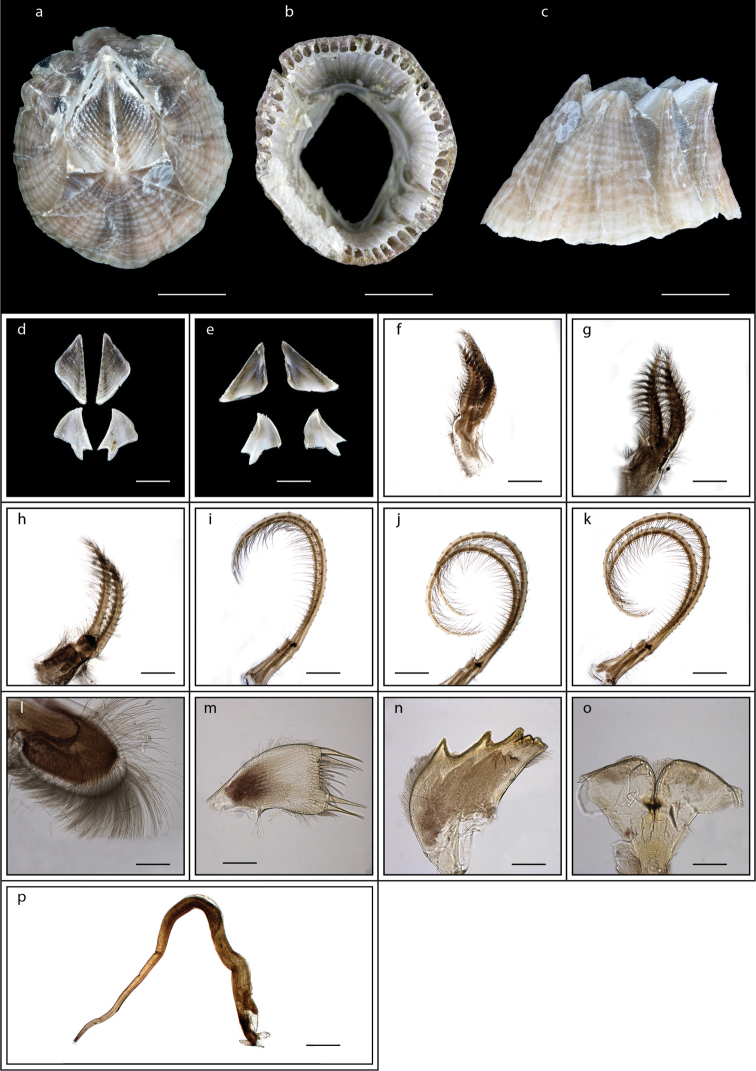
*Amphibalanus variegatus* (Darwin, 1854) (MZB Cru Cir 014-1) **a** upper view **b** lower view **c** side view **d** external view of scutum and tergum **e** internal view of scutum and tergum **f** cirrus I **g** cirrus II **h** cirrus III **i** cirrus IV **j** cirrus V **k** cirrus VI **l** maxilla **m** maxillule **n** mandible **o** labrum **p** penis. Scale bars: 3 mm (**a–c**); 1 mm (**d–h**); 2 mm (**i–k**); 2 mm (**p**); 0.5 mm (**l–o**).

Figure 23a–p , Table 1: species no. 89

Balanus amphitrite var. (8) variegatus Darwin, 1854: 241.
Balanus amphitrite var. stutsburi Krüger, 1914: 437.
Balanus concavus sinensis Broch, 1931: 63, fig. 23.
Balanus amphitrite rafflesia Nilsson-Cantell, 1934a: 64.
Balanus amphitrite var. cirratus : Pope 1945: 362, pl. 28 fig. 6, pl. 29 fig. 6, pl. 30 figs 13, 14.
Balanus amphitrite cirratus : Skerman 1960: 610, figs 1, 3 (**non**Balanus amphitrite cirratus Darwin, 1854).
Balanus variegatus : Harding 1962: 291, pl. 10 figs a–k; Zevina et al. 1992: 92, fig. 64.
Balanus variegatus var. cirratus : Pope 1966: 179.
Balanus amphitrite : Foster 1967: 83 (part.).287.
Balanus kondakovi : Henry & McLaughlin 1975: 78 (part., New Zealand specimens; **non**B. kondakovi Tarasov & Zevina, 1957).
Balanus variegatus variegatus : Foster 1979: 111, fig. 67, pl. 14b.
Balanus cirratus : Ren & Liu, 1978: 145, figs 14, 15 (1–13), pl. 4 figs 15–20, pl. 5 figs 1–6.non Balanus amphitrite variegatus: Nilsson-Cantell 1934a: 60.
non Balanus variegatus: Henry & McLaughlin 1975: 78, fig. 17, pls. 6, 7; Utinomi 1968b: 171 (= B. cirratus).
Amphibalanus variegatus : Pitombo 2004: 274; Horikoshi and Okamoto 2005: 49, fig.3.

####### Material examined.

***Ambon Island***: 15 specimens, MZB Cru Cir 014, Waitatiri, 3°37'04.0"S ,128°16'20.3"E, coll. P. Pitriana & D. Tala, 19 Sep 2017. ***Saparua Island***: 10 specimens, MZB Cru Cir 015, Teluk Saparua, 3°34'25.7"S, 128°39'25.8"E, coll. P. Pitriana & D. Tala, 22 Sep 2016.

####### GenBank accession numbers.

COI gene (MK995342–MK995345), 18S (MK981355).

####### Diagnosis.

Primary parietal tubes with transverse septa; exterior of shell with longitudinal and horizontal striations; anterior margin of cirrus III without conical denticles, erect hooks below posterior angles of distal articles of rami absent.

####### Description.

Shell steeply conical, tubular in crowded populations; six parietal plates, smooth, thin, brownish purple externally with longitudinal stripes crosshatched by transverse bands, single row of internal tubes (Fig. 23b); carina forming a spout-like projection; radii wide, summits oblique, pink-purple; alae with summits oblique; orifice toothed (Fig. 23a–c); sheath purple with white bands, vesicular; basis calcareous, porous; scutum externally with growth lines prominent, internally with articular ridge high, adductor ridge moderately long; tergum with spur furrow, externally purple, margins white, spur pointed, basal margin deeply excavated on either side of spur, depressor muscle crests prominent(Fig. 23d, e); cirrus III without conical denticles on anterior margin (Fig. 23h); maxilla without notch (Fig. 23l), mandible with four teeth (Fig. 23n), labrum notched, denticulate (Fig. 23o); penis with basidorsal point, with two apical setae (Fig. 23p). Basal length (8.3–11.8 mm, basal width 6.9–10.4 mm, height 4.3–8.4 mm; orifice length 4.8–11.8 mm; orifice width 3.4–5.3 mm (measurements for ten specimens are presented in Suppl. material 1: Table S22).

####### Distribution.

*Amphibalanus variegatus* has been reported from the Indo-west Pacific: Bay of Bengal; Sumatra; New Zealand, Australia; Indonesia; Singapore; Vietnam; Gulf of Siam; Hong Kong; W Kyushu; Vladivostok; and is a common fouling species (Henry and McLaughlin 1975; Jones and Hosie 2016). In this study, *A. variegatus* was found on the islands of Ambon (at Waitatiri) and Saparua (at Teluk Saparua) on stones and a plastic bag (a map with the occurrence of *Amphibalanus variegatus* in the Moluccas is shown in Suppl. material 1: Fig. S4).

####### Remarks.

*Amphibalanus variegatus* is a member of the *Balanus amphitrite* complex, whose members can be difficult to distinguish morphologically. *Amphibalanus variegatus* can be differentiated by its vesicular sheath, and from *A. reticulatus* by features of the tergum, armature of cirrus II and the lack of erect teeth below the posterior distal angles of cirri III–VI (Henry and McLaughlin 1975).

###### Amphibalanus zhujiangensis

Taxon classificationAnimaliaSessiliaBalanidae

(Ren, 1989)

B8CF5AC2-526E-5233-BD83-DD06BB9D9EDF

**Figure 24.**
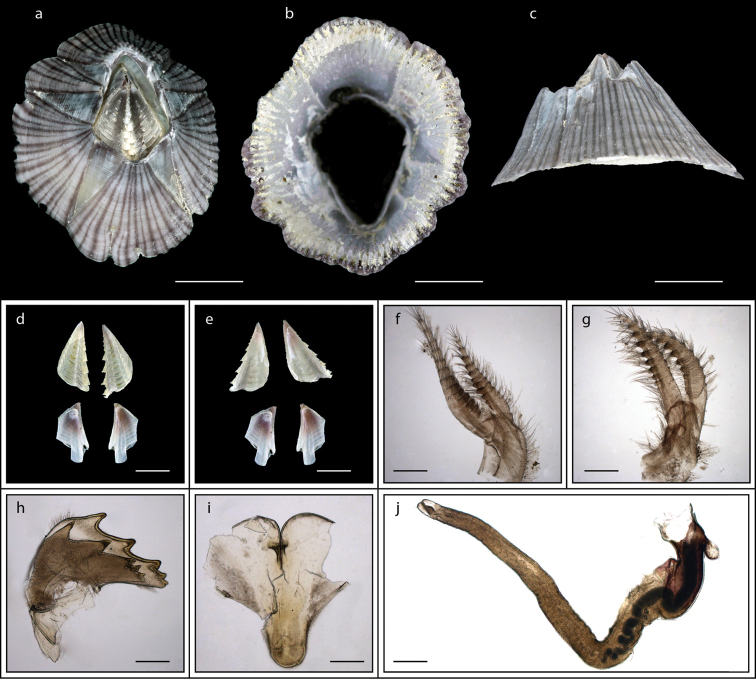
*Amphibalanus zhujiangensis* (Ren, 1989) (MZB Cru Cir 018-5) **a** upper view **b** lower view **c** side view **d** external view of scutum and tergum **e** internal view of scutum and tergum **f** cirrus I **g** cirrus II **h** mandible **i** labrum **j** penis. Scale bars: 6 mm (**a–c**); 1 mm (**d–g**); 0.5 mm (**h, i**); 2 mm (**j**).

Figure 24a–j , Table 1: species no. 90

Balanus zhujiangensis Ren, 1989a: 467, fig. 2 (1–14).
Amphibalanus zhujiangensis : Pitombo 2004: 274; Puspasari et al. 2002: 235, figs 1A–G, 2A–H; Liu and Ren 2007: 501; Chan et al. 2009a: 238, fig. 204.

####### Material examined.

***Ambon Island***: 10 specimens, MZB Cru Cir 016, Galala, 3°41'22.2"S, 128°10'52.6"E, coll. P. Pitriana & D. Tala, 6 Sep 2016; 3 specimens, MZB Cru Cir 017, Laha, 3°43'22.5"S, 128°05'02.5"E, coll. P. Pitriana & D. Tala, 5 Sep 2016; 6 specimens, MZB Cru Cir 018, Talake, 3°41'59.4"S, 128°10'19.2"E, coll. P. Pitriana & D. Tala, 5 Sep 2016. ***Saparua Island***: 10 specimens, MZB Cru Cir 019, Desa Pia, 3°30'20.4"S, 128°36'55.0"E, coll. P. Pitriana & D. Tala, 21 Sep 2016; 7 specimens, MZB Cru Cir 020, Negeri Mahu, 3°31'52.9"S, 128°41'12.4"E, coll. P. Pitriana & D. Tala, 11 Apr 2016; 1 specimen, MZB Cru Cir 021, Desa Mahu, 3°32'19.6"S, 128°41'17.3"E, coll. P. Pitriana & D. Tala, 11 Apr 2016. ***Seram Island***: 5 specimens, MZB Cru Cir 022, Desa Kasie, 2°51'05.5"S, 128°32'54.1"E, coll. P. Pitriana & D. Tala, 20 Sep 2017; 3 specimens, MZB Cru Cir 010, Lepas Pantai Kawa, 2°57'32.5"S, 128°05'33.4"E, coll. P. Pitriana & D. Tala, 19 Sep 2017.

####### GenBank accession numbers.

COI gene (MK995334, MK995336, MK995337, MK995339), 18S (MK981347, MK981349, MK981350, MK981352).

####### Diagnosis.

Primary parietal tubes without transverse septa; exterior of shell with longitudinal striations; scutum without adductor ridge, external surface scutum with row of pits; anterior margin of cirri III with conical denticles, erect hooks below posterior angles of distal articles of rami present; cirrus IV with erect hooks on posterodistal angles of articles.

####### Description.

Shell six-plated, conic, purplish-white with longitudinal stripes of purple, not cross-hatched by transverse striations; parietes externally smooth, parietal tubes lacking transverse septa and subsidiary tubes; radii wide with slightly oblique summits; orifice rhomboidal, toothed (Fig. 24a–c); scutum trigonal, exterior of scutum with single row of pits extending down centre of valve, occluding margin toothed, inner surface smooth, adductor ridge lacking; tergum with carinal margin convex, spur furrow open, basal margin straight on both sides of spur (Fig. 24d, e); cirrus III without complex setae; cirri III–VI with erect hooks around posterior angle; first maxilla without notch, mandible with five teeth (Fig. 24h). Basal length 5.8–21.6 mm; basal width 4.8–19.2 mm; height 2.1–16.5 mm; orifice length 3.0–10.0 mm; orifice width 2.6–7.6 mm (measurements for eleven specimens are presented in Suppl. material 1: Table S23).

####### Distribution.

*Amphibalanus zhujiangensis* was first recorded from the estuary of the Zhujiang River, South China Sea (Puspasari et al. 2002). Afterwards found on Okinawa Island, Japan and Taiwan (Chan et al. 2009a). In this study, *A. zhujiangensis* was found on the islands of Ambon Island (at Galala, Laha, and Talake), Saparua Island (at Dusun Pia, Negeri Mahu, Desa Mahu), and Seram Island (at Desa Kasie, Lepas Pantai Kawa) on stone and capitulum of *Lepas anserifera* (a map with the occurrence of *Amphibalanus zhujiangensis* in the Moluccas is shown in Suppl. material 1: Fig. S8).

####### Remarks.

The presence of a row of pits on the external surface of the scutum and the absence of an adductor ridge on the scutum are diagnostic for *A. zhujiangensis*. The species can be distinguished from *A. variegatus* by characters of the shell, cirri III and cirri IV; on *A. reticulatus* by characters of the shell and first maxilla. *Amphibalanus zhujiangensis* is distinct from *A. thailandicus* in lacking transverse septa in the longitudinal tubes and a notch on the first maxilla (Puspasari et al. 2002).

###### Amphibalanus

Taxon classificationAnimaliaSessiliaBalanidae

sp.

B2807A72-7207-5E32-918B-ED0FB1F4F7AA

**Figure 25.**
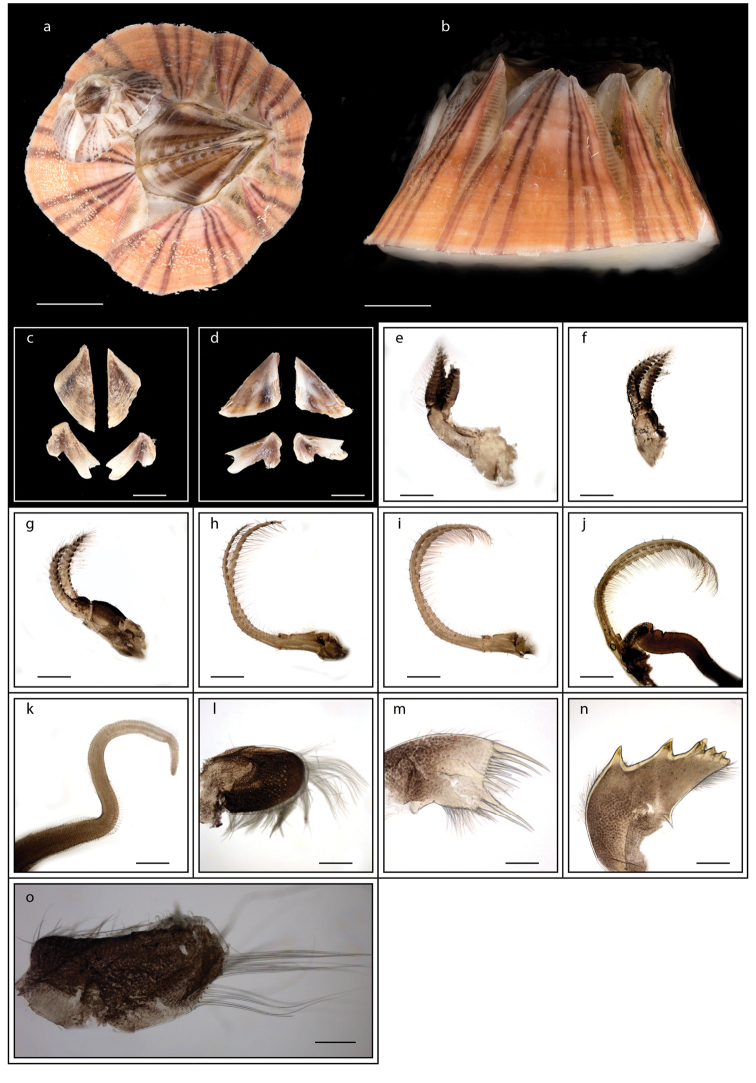
*Amphibalanus* sp. (MZB Cru Cir 135) **a** upper view **b** side view **c** external view of scutum and tergum **d** internal view of scutum and tergum **e** cirrus I **f** cirrus II **g** cirrus III **h** cirrus IV **i** cirrus V **j** cirrus VI **k** penis **l** maxilla **m** maxillule **n** mandible **o** mandibular palp. Scale bars: 3 mm (**a, b**); 1 mm (**c–g**); 2 mm (**h–k**); 0.5 mm (**l–o**).

Figure 25a–o , Table 1: species no. 91

####### Material examined.

***Ambon Island***: 1 specimen, MZB Cru Cir 135, Talake, 3°41'59.4"S, 128°10'19.2"E, coll. P. Pitriana & D. Tala, 5 Sep 2016; 1 specimen, MZB Cru Cir 136, Waitatiri, 3°37'04.0"S, 128°16'20.3"E, coll. Adin, 19 Sep 2017. ***Seram Island***: 2 specimens, MZB Cru Cir 137, Dermaga Pelita Jaya, 3°00'13.5"S, 128°07'09.2"E, coll. P. Pitriana & D. Tala, 21 Sep 2017.

####### GenBank accession numbers.

COI gene (MK995349–MK995351, MK995353), 18S (MK981356–MK981358, MK981360).

####### Diagnosis.

Primary parietal tubes with transverse septa, exterior of shell with longitudinal striations; orifice toothed; scutum without adductor ridge; anterior margin of cirri III with conical denticles, erect hooks below posterior angles of distal articles of rami present; cirrus IV with erect hooks on posterodistal angles of articles; basidorsal of penis absent.

####### Description.

Shell six-plated, conical, whitish with dark purple transverse stripes (Fig. 25a, b); orifice slightly toothed; scutum trigonal, occluding margin toothed; tergum with closed spur furrow (Fig. 25c, d); mandible with five teeth (Fig. 25n); maxilla bilobed with dense setae only on lower margin (Fig. 25l). Basal length 7.4–12.2 mm, basal width 6.3–11.8 mm, height 5.5–9.4 mm; orifice length 3.6–8.3 mm; orifice width 2.6–5.9 mm (measurements for four specimens are presented in Suppl. material 1: Table S24).

####### Distribution.

In this study, *Amphibalanus* sp. was found on Ambon (at Talake and Waitatiri) and Seram islands (at Dermaga Pelita Jaya) (a map with the occurrence of *Amphibalanus* sp. in the Moluccas is shown in Suppl. material 1: Fig. S9).

####### Remarks.

In the molecular phylogeny, *Amphibalanus* sp. forms a well-supported clade in both, the COI and the 18S tree (Figs 28, 29). This species also has different maxilla than those of *A. amphitrite*, *A. reticulatus* and *A. variegatus*, which have setae on its upper and lower margins.

##### Subfamily Megabalaninae Newman, 1979

**Genus *Megabalanus* Hoek, 1913**

###### Megabalanus tintinnabulum

Taxon classificationAnimaliaSessiliaBalanidae

(Linnaeus, 1758)

9B02B720-4166-57BF-BE9B-EC6C4AED7BDC

**Figure 26.**
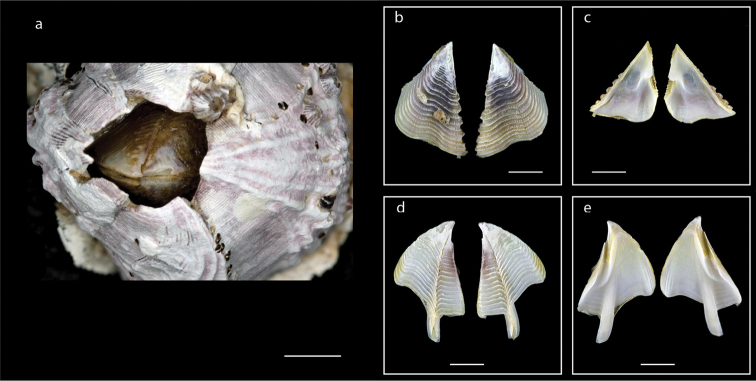
*Megabalanus tintinnabulum* (Linnaeus, 1758) (MZB Cru Cir 066-3) **a** upper view **b** external view of scutum **c** internal view of scutum **d** external view of tergum **e** internal view of tergum. Scale bars: 8 mm (**a**); 5 mm (**b–e**).

Figure 26a–e , Table 1: species no. 97

Balani Rhumphius, 1705: 121, pl. 41 figs A, C, D.
Balanus tintinnabuliformis laevis Lang, 1772: 4.
Balanus cylindraceus unicum thalamum efformans, *magnis ventricosus* Gaultierus, 1742: un-numbered page, pl. 106, fig. H. *Glands de mer de la grande espèce* Dezallier d’Argenville, 1742: 364, pl. 30 fig. A; 1757: 364, pl. 26 fig. A.
Lepas Tintinnabulum Linnaeus, 1758: 668; Chemnitz 1785 (part.): pl. 97 figs 830, 831 (non figs 828, 829).
Lepas calyciformis orientalis Ellis, 1758: 845, pl. 34 figs 8, 9.
Balanus tintinnabulum : Bruguière 1789 (part.): 165; Holthuis and Heerebout 1972: 24, pl. 1.
Lepas tintinnabulum : Wood 1815: 38, pl. 6 figs 1, 2.
Lepas spinosa Wood, 1815 (part.): pl. 7 fig. 4 (large shell only; small shells = M. spinosus).
Balanus tintinnabulum var. (1) communis Darwin, 1854: 195, pl. 1 figs a, b, f supra, pl. 2 figs 1 a, 1 c–e, 1 i, 1 k.
Balanus tintinnabulum var. communis : Gruvel 1905a: 21.
Balanus tintinnabulum tintinnabulum : Pilsbry 1916: 55, fig. 9, pl. 10 figs 1a–e; Dong et al. 1982: 86.
Balanus tintinnabulum antillensis Pilsbry, 1916: 63, pl. 13 figs 1, 2 e.
Balanus (Megabalanus) tintinnabulum forma communis Broch, 1931: 56.
Balanus tintinnabulum var. tintinnabulum ; Oliveira 1941: 11, fig. 1, pl. 2 figs 1, 2, pl. 4 fig. 1, pl. 5 fig. 3, pl. 8 fig. 6.
Megabalanus antillensis Newman & Ross, 1976: 67.
Balanus (Megabalanus) tintinnabulum tintinnabulum : Ren & Liu, 1978: 121, fig. 1, pl. 1 figs 1–5.
non Lepas tintinnabulum: Spengler 1790: 180 [= Megabalanus occator (Darwin, 1854)]
non Lepas tintinnabulum var. a: Spengler 1790: 181 (*incertae sedis*).
non Lepas tintinnabulum var. b: Spengler 1790: 182 [= Striatobalanus amaryllis (Darwin, 1854)]
non Lepas tintinnabulum: Chemnitz 1785: pl. 97, figs 828, 829 [= Austromegabalanus nigrescens (Lamarck, 1818)].
non Balanus tintinnabulum: Chenu 1843: pl. 2 fig. 8, pl. 3 fig. 5, pl. 2 fig. 8 [= Megabalanus ajax (Darwin, 1854)]; pl. 3 fig. 5 [= Megabalanus tulipiformis (Darwin, 1854)].
non Balanus tintinnabulum var. communis: Krüger 1911a: 46, pl. 3 figs 31 a1–31 b2 [= Megabalanus volcano Pilsbry, 1916)].
non Balanus (Megabalanus) tintinnabulum: Withers 1924: pl.6 figs 4–7 [= Megabalanus linzei (Foster, 1979)].
non Balanus tintinnabulum antillensis Pilsbry, 1927: 38, fig. 3 a–c [= Megabalanus stultus (Darwin, 1854)]
non Balanus tintinnabulum tintinnabulum: Linzey 1942: 279 [= Megabalanus linzei (Foster, 1979)].
non Balanus tintinnabulum: Foster 1967: 81, fig. 2a, b [= Megabalanus linzei (Foster, 1979)].
Megabalanus tintinnabulum : Newman & Ross, 1976: 68; Henry & McLaughlin 1986: 17, figs 1e, 2a, g, h, 3a–c, 5 a–l; Zevina et al. 1992: 99, fig. 67; Pitombo 2004: 175; Chan et.al 2009a: 259, fig. 224; Pochai et.al 2017: 28, fig.11.

####### Material examined.

***Ambon Island***: 3 specimens, MZB Cru Cir 066, Laha, 3°43'22.5"S, 128°05'02.5"E, coll. P. Pitriana & D. Tala, 5 Sep 2016. ***Saparua Island***: 1 specimen, MZB Cru Cir 067, Desa Pia, 3°30'20.4"S, 128°36'55.0"E, coll. P. Pitriana & D. Tala, 21 Sep 2016.

####### Diagnosis.

Shell relatively large, lightly ribbed; radii wide; surface smooth without spines; tergum wider than scutum with spur narrow and long, crests for depressor muscle weakly to moderately well developed.

####### Description.

Shell cylindrical to conical, parietes purplish, smooth, with longitudinal purple striations, tubiferous (Fig. 26a); radii wide, usually horizontally striated, summits horizontal, sutural edges with regular denticles; summits of alae oblique; orifice rhomboidal, moderately small to large, one-third to two-thirds basal diameter, subcircular to subtriangular; scutum triangular, external surface with horizontal striations, inner surface with conspicuous articular ridge; tergum triangular, frequently wider than scutum, external surface with horizontal striations, spur long, narrow, external surface with median furrow, scutal margin denticulate (Fig. 26b–e); mandible with five teeth, labrum with deep cleft, three teeth on each side. Basal length 26.0–49.2 mm, basal width 29.0–43.1 mm, height 20.1–49.4 mm. Orifice length 13.7–16.3 mm, orifice width 10.4–15.9 mm (measurements for four specimens are presented in Suppl. material 1: Table S25).

####### Distribution.

*Megabalanus tintinnabulum* is a cosmopolitan species and widely distributed worldwide (Pochai et al. 2017). In this study, *M. tintinnabulum* was found on Ambon Island (at Laha) and in Saparua Island (at Desa Pia) on concrete bridge at the port, stones and reef surface (a map with the occurrence of *Megabalanus tintinnabulum* in the Moluccas is shown in Suppl. material 1: Fig. S4).

####### Remarks.

The name *Megabalanus* was given by Hoek (1913), referring to the largest form of existing Balani. With the exception of *Balanus amphitrite*, Darwin (1854) considered *Balanus tintinnabulum* as the most difficult and variable species in the genus *Balanus* (Henry and McLaughlin 1986). *Megabalanus tintinnabulum* can be distinguished by its large shell plates and purple surface with irregular, unclear longitudinal stripes (Pochai et al. 2017).

###### Megabalanus zebra

Taxon classificationAnimaliaSessiliaBalanidae

(Darwin, 1854)

B6A7D498-CDE5-59FE-8B07-9B0B3C750426

**Figure 27.**
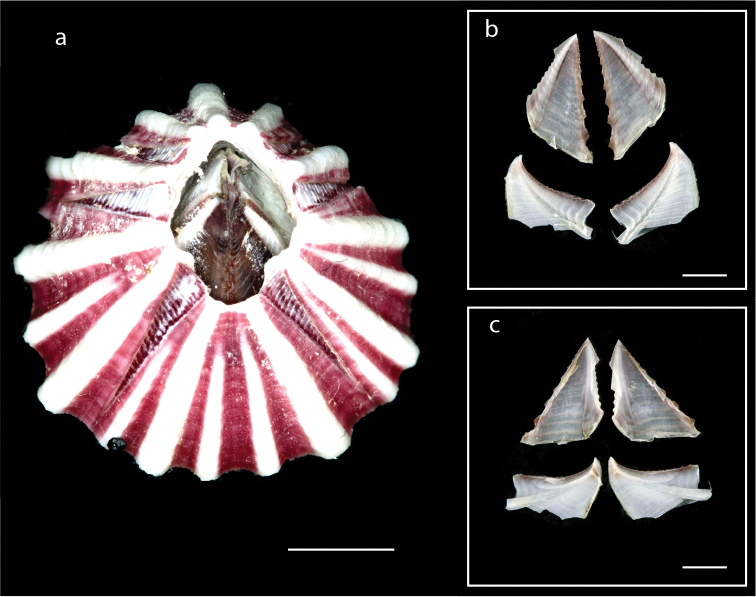
*Megabalanus zebra* (Darwin, 1854) (MZB Cru Cir 068-3) **a** upper view **b** external view of scutum and tergum **c** internal view of scutum and tergum. Scale bars: 4 mm (**a**); 2 mm (**b–c**).

Figure 27a–c , Table 1: species no. 98

Balanus tintinnabulum var. (4) zebra Darwin, 1854: 195. pl. 1 fig. g.
Balanus tintinnabulum zebra : Pilsbry 1916: 57, pl. 10 figs 2, 3; Stubbings 1967: 264; Dong et al. 1982: 86, fig. A–C.
Balanus tintinnabulum var. zebra Karande & Palekar, 1966: 143, pl. I, fig. 2.
Megabalanus zebra : Newman & Ross, 1976: 69; Henry and McLaughlin 1986: 47, figs 2f, 4j–k, 12e–l; Pitombo 2004: 275; Chan et al. 2009a: 265, fig. 232; Pitombo et al. 2017: 135, figs 2, 4, 5, 6, 7, 8.

####### Material examined.

***Ambon Island***: 4 specimens, MZB Cru Cir 068, Galala, 3°41'22.2"S, 128°10'52.6"E, coll. P. Pitriana & D. Tala, 6 Sep 2016; 4 specimens, MZB Cru Cir 069, Laha, 3°43'22.5"S, 128°05'02.5"E, coll. P. Pitriana & D. Tala, 5 Sep 2016.

####### Diagnosis.

Parietes reddish purple with strong longitudinal white ribs; radii and sheath dark purple to reddish brown; scutum with narrow tergal segment slightly inflected; tergum approximately as wide as scutum, crest for depressor muscle prominent.

####### Synoptic Description.

Shell conic, six-plated; parietes smooth, purple with well-developed white ribs and dark purple interspaces; radii wide, summits horizontal, white with dark purple spots on proximal side (Fig. 27a); orifice rhomboidal; scutum triangular, external surface withe intersecting horizontal and longitudinal striations; tergum triangular, white with purple spots in some areas, scutal margin slightly curved, basal margin inclined, spur short (Fig. 27b, c) ; mandible with five teeth, labrum with deep cleft, three teeth on each side. Basal length 5.4–19.0 mm, basal width 5.0–20.0 mm, height 4.4–13.1 mm. Orifice length 3.1–7.6 mm, orifice width 1.7–6.2 mm (measurements for eight specimens are presented in Suppl. material 1: Table S26).

####### Distribution.

*Megabalanus zebra* is a well-known fouling species of ship hulls, floating structures, moveable oil platforms, etc., and has been widely recorded from the Atlantic Ocean; W Africa; Indo-west Pacific: Indian Ocean; Australia; Thailand; China; Philippines; Taiwan (Pilsbry 1896, 1916; Stubbings 1961; Karande and Pakelar 1966; Foster and Willan 1979; Chan et al. 2009a; Jones and Hosie 2016; Pitombo et al. 2017). In this study, *Megabalanus zebra* was found on Ambon Island (at Galala and Laha) on stones and the capitulum of *Lepas anserifera* (a map with the occurrence of *Megabalanus zebra* in the Moluccas is shown in Suppl. material 1: Fig. S1).

####### Remarks.

*Megabalanus zebra* can be distinguished from other species in the *Megabalanus* group by, for example, the inflection of the tergal segment of the scutum and the position of the spur (Henry and McLaughlin 1986). *M. zebra* can also be differentiated by an intermediate shape of the tergum and scutum compared to *M. tintinnabulum* and *M. coccopoma* (Pitombo et al. 2017).

### Molecular results

**Figure 28.**
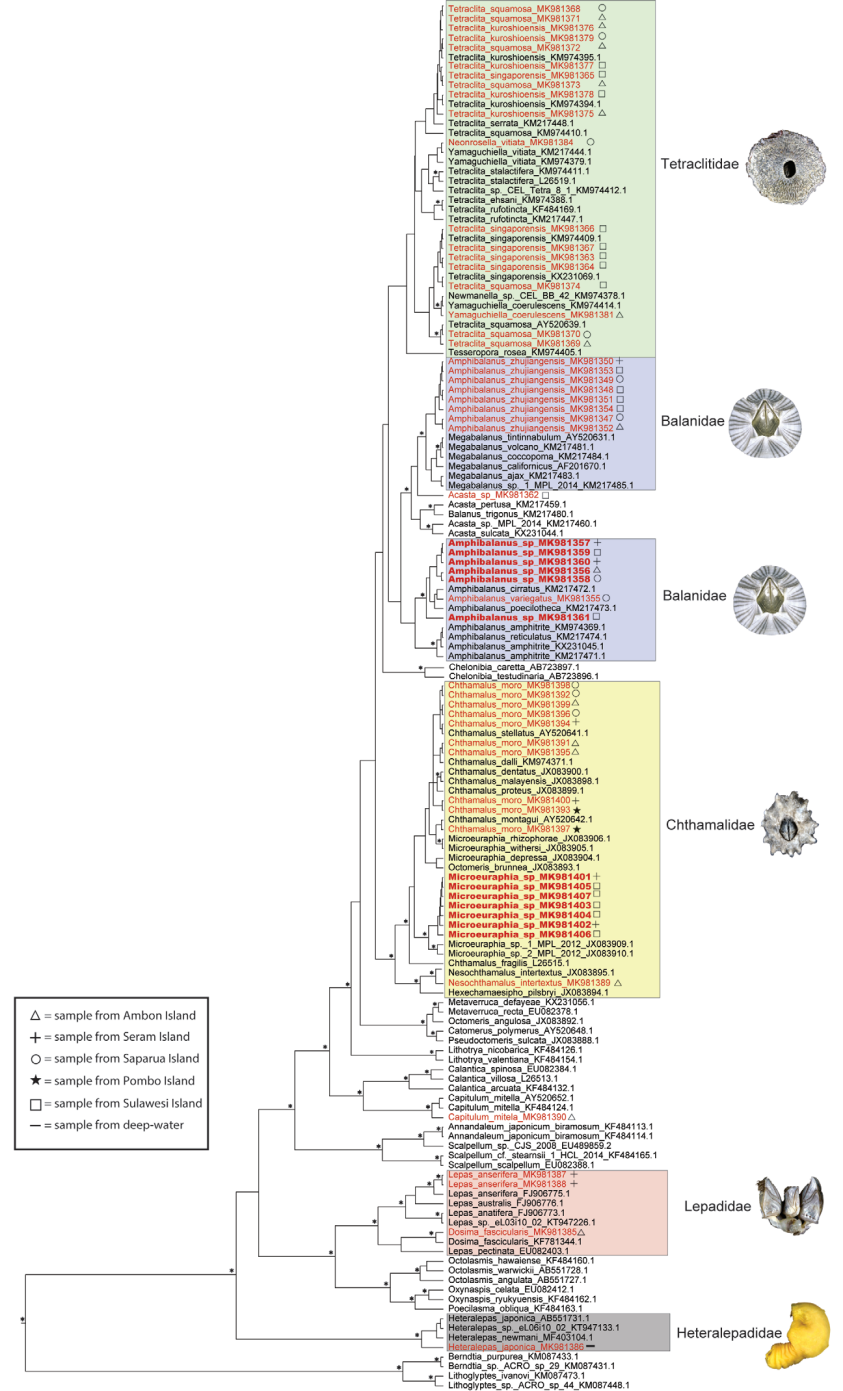
Bayesian phylogeny of 18S gene sequences. High Bayesian posterior probabilities (≥ 0.85) are indicated by an asterisk at the respective node. Families with relevance for this study are highlighted by coloured rectangles. Sample labels in red indicate sequences newly generated for this study. Species names in bold indicate potential new species.

**Figure 29.**
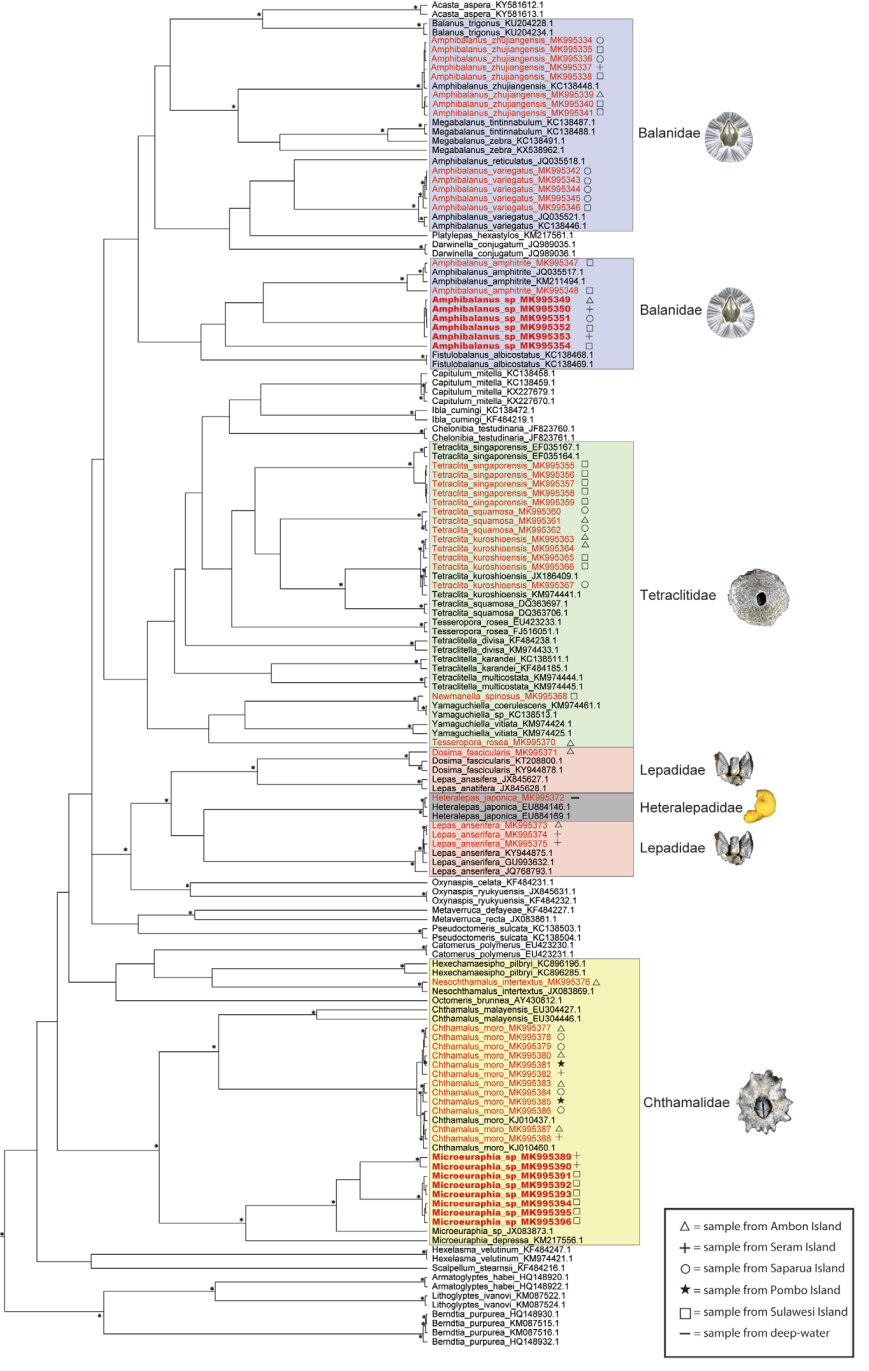
Bayesian phylogeny of COI gene sequences. High Bayesian posterior probabilities (≥ 0.85) are indicated by an asterisk at the respective node. Families with relevance for this study are highlighted by coloured rectangles. Sample labels in red indicate sequences newly generated for this study. Species names in bold indicate potential new species.

In total, we produced 120 new sequences for this study (COI = 62 sequences, 18S = 58 sequences; Suppl. material 1: Table S1; Figs 28, 29). We downloaded a total of 172 sequences from GenBank (COI = 84 sequences, 18S = 88 sequences). The final COI alignment used for phylogenetic analyses was 641 bp long, and included 156 sequences from 50 species (Fig. 29). The 18S alignment was 1918 bp long and included 154 sequences from 83 species (Fig. 28).

In general, support values (bootstrap and posterior probability) were low for both markers, with the majority of internal nodes receiving support values below 50% bootstrap or 0.5 posterior probability. However, there are several highly supported nodes throughout (> 70% bootstrap; > 0.85 posterior probability), which allow us to gain insights into the evolutionary history of the group. In general, and as expected, COI (Fig. 29) provided higher resolution at terminal nodes but low resolution at deeper nodes (rapidly evolving marker), whereas 18S (Fig. 28) provided higher resolution than COI at basal nodes (slowly evolving marker). The trees resulting from the BEAST and RaxML analyses were fully congruent, with no highly supported relationships being favoured in one analysis but not the other. The main purpose of our phylogenetic analyses was to find out where the new accessions from the Moluccas sequenced for this study are retrieved on the barnacle tree, and to see whether putative species are retrieved as monophyletic. We therefore show multiple accessions per species on the trees. The trees resulting from RaxML analyses and the concatenated analyses are given in the Suppl. material (Suppl. material 1: Figs S10–S13).

### Molecular study of Moluccan barnacles

The vast majority of new samples from the Moluccas produced in this study matched sequences from the same species that are available on GenBank. For example, DNA sequence of our *Heteralepas japonica* matched the sequence of *H. japonica*EU884146.1 and EU884169.1 from Chan, et al. (2009c); and our *Nesochathamalus intertextus* matched the sequence of *N. intertexus*JX083869.1 from Perez-Losada, et al. (2012). This applies to all species for which we have new sequences. The only exception is *Chthamalus moro*, for which one of our samples in the 18S tree does not match the GenBank samples of that species. However, for this particular case, the support values of that clade in the tree are very low, therefore the odd positioning is not strongly supported (that clade is essentially a polytomy).

Two taxa for which we sequenced multiple accessions, but for which we could not assign a species name, were retrieved in positions on the tree that lead us to propose these may constitute new unidentified species. The first one is *Amphibalanus* sp., clustering as a unit in both COI and 18S trees (Figs 28, 29; Suppl. material 1: Figs S10–S13). The other was *Microeuraphia* sp., which formed well supported and separated clades in the COI tree, and was clustered in the same unresolved clade in 18S (Figs 28, 29; Suppl. material 1: Figs S10–S13).

The K2P distances within *Microeuraphia* sp. were 1.74%±0.51% for the COI sequences. The K2P distances between *Microeuraphia* sp. and other species ranged from 10.90% to 22.70%; and overall averaged distances between the species and other species were 13.82% (Suppl. material 1: Table S27). Whilst for *Amphibalanus* sp. the K2P distances within the species were 0.22%±0.13% for the COI sequences. The K2P distances between *Amphibalanus* sp. and other species ranged from 13.34% to 18.33%; and overall averaged distances between the species and other species were 14.37% (Suppl. material 1: Table S28).

## Discussion

This checklist lists 97 species, including 23 new records the Moluccas and two of which still await their species descriptions. The past record on barnacles from these islands dates back to the *Challenger* (1872–1876) and *Siboga* (1899–1900) expeditions (Hoek 1913). Later, Kolosváry (1950) only mentioned some balanids living in corals collected during *Snellius* Expedition (1929–1930), which contrasts reports from other groups numerously collected during the same expedition, such as in decapod crustaceans and Foraminifera.

Hoek (1913) listed a total of 210 species from the Malay Archipelago that were collected during the *Challenger* and *Siboga* expeditions. Among these, 45 species were found in the Moluccas. However, the majority of the Moluccan species listed by Hoek (32 species) were deep-water barnacles found at depths of 204–2,798 m, while 10 species barnacles were found at depths of 9–90 m, and only three inshore species were recorded: *Temnaspis fissum* (Darwin, 1851) from Ternate; *Yamaguchiella coerulescent* (Spengler, 1790) and *Tetraclitella costata* (Darwin, 1854) from Banda Island. In contrast, sampling for this study focused on inshore habitats with only two deep-sea locations. In consequence to the different sampling approaches, we found 24 inshore species and only one deep-sea species among the new samples.

A comparison of the number of species previously recorded from Ambon, Seram, and Banda by Hoek (1913) and Jones (2001, 2016) with those recorded in this study indicates that species diversity for each island has been heavily underestimated. On Ambon, for example, seven species were previously known compared to the 24 species listed here. For other smaller islands such as Saparua and Pombo, no barnacle species was previously recorded. Given the size of the Moluccan Archipelago, with ca. 1,000 islands, many of which have never been sampled despite including relatively large islands such as Haruku, Buru, Yamdena or Wetar, a much higher number of species can be expected in the Moluccas.

The molecular results also indicate that the barnacle fauna of the region is understudied. In addition to evidence for two potentially new species (see above), the generic assignment of some described species is also challenged. For example, *Amphibalanus zhujiangensis* was found to be more closely related to *Megabalanus* than to other *Amphibalanus* species, suggesting the need to conduct in-depth research on this species to clarify its taxonomy. However, we must caution against over interpretation of our phylogenetic trees, because the markers we used revealed low node support overall.

The molecular phylogeny failed to reveal any biogeographic pattern of barnacles from the Moluccas, which is not surprising given the limited scope of sampling. These points all underline again the necessity of a more comprehensive approach to sampling in the region as well as the need to explore more molecular markers for a truly integrative taxonomy of barnacles, not just in the Moluccas.

## Supplementary Material

XML Treatment for Heteralepas japonica

XML Treatment for Dosima fascicularis

XML Treatment for Lepas anserifera

XML Treatment for Capitulum mitella

XML Treatment for Pseudoctomeris sulcata

XML Treatment for Hexechamaesipho pilsbryi

XML Treatment for Nesochthamalus intertextus

XML Treatment for Euraphia hembeli

XML Treatment for Microeuraphia

XML Treatment for Chthamalus moro

XML Treatment for Tetraclitella divisa

XML Treatment for Tetraclitella karandei

XML Treatment for Tesseropora rosea

XML Treatment for Tetraclita kuroshioensis

XML Treatment for Tetraclita squamosa

XML Treatment for Yamaguchiella coerulescens

XML Treatment for Neonrosella vitiata

XML Treatment for Newmanella spinosus

XML Treatment for Amphibalanus amphitrite

XML Treatment for Amphibalanus reticulatus

XML Treatment for Amphibalanus variegatus

XML Treatment for Amphibalanus zhujiangensis

XML Treatment for Amphibalanus

XML Treatment for Megabalanus tintinnabulum

XML Treatment for Megabalanus zebra

## Supplementary materials

Supplementary material 1 Tables S1–S28, Figs S1–S13 Data type species data Explanation note **Fig. S1** The occurrence of *Heteralepas japonica* in the Moluccas (red dots); **Fig. S2** The occurrence of *Amphibalanus reticulatus*, *Dosima fascicularis*, *Euraphia hembeli*, *Hexechamaesipho pilsbryi*, *Megabalanus zebra*, *Nesochthamalus intertextus*, *Newmanella spinosus*, *Pseudoctomeris sulcata*, *Tetraclitella divisa* and *Tetraclitella karandei* in the Moluccas (the red dot indicates that the species occurs on the coastline of the island); **Fig. S3** The occurrence of *Chthamalus moro* and *Lepas anserifera* in the Moluccas (the red dots indicate that the species occurs on the coastline of the islands); **Fig. S4** The occurrence of *Amphibalanus amphitrite*, *Amphibalanus variegatus*, *Capitulum mitella*, *Megabalanus tintinnabulum*, *Tesseropora rosea*, *Tetraclita kuroshioensis* and *Tetraclita squamosa* in the Moluccas (the red dots indicate that the species occurs on the coastline of the islands); **Fig. S5** The occurrence of *Microeuraphia* sp. in the Moluccas (the red dot indicates that the species occurs on the coastline of the island); **Fig. S6** The occurrence of *Yamaguchiella coerulescens* in the Moluccas (the red dots indicate that the species occurs on the coastline of the islands); **Fig. S7** The occurrence of *Neonrosella vitiata* in the Moluccas (the red dots indicate that the species occurs on the coastline of the islands); **Fig. S8** The occurrence of *Amphibalanus zhujiangensis* in the Moluccas (the red dots indicate that the species occurs on the coastline of the islands); **Fig. S9** The occurrence of *Amphibalanus* sp. in the Moluccas (the red dots indicate that the species occurs on the coastline of the islands); **Fig. S10** Bayesian phylogeny of concatenated COI and 18S gene sequences. Values next to nodes are support values; **Fig. S11**ML phylogeny of concatinated COI and 18S gene sequences. Values next to nodes are support values; **Fig. S12**ML phylogeny of 18S gene sequences. Tips coloured in red are the new samples sequenced for this manuscript. Values next to nodes are support values; **Fig. S13**ML phylogeny of COI gene sequences. Tips coloured in red are the new samples sequenced for this manuscript. Values next to nodes are support values; **Table S1.** Table of sample information for DNA samples sequenced for this study; **Table S2.** Measurements for *Heteralepas japonica* (N = 25); **Table S3.** Measurements for *Dosima fascicularis* (N = 6); **Table S4.** Measurements for *Lepas anserifera* (N = 25); **Table S5.** Measurements for *Capitulum mitella* (N = 25); **Table S6.** Measurements for *Pseudoctomeris sulcata* (N = 2); **Table S7.** Measurements for *Hexechamaesipho pilsbryi* (N = 10); **Table S8.** Measurements for *Nesochthamalus intertextus* (N = 10), **Table S9.** Measurements for *Euraphia hembeli* (N = 1); **Table S10.** Measurements for *Microeuraphia* sp. (N = 2); **Table S11.** Measurements for *Chthamalus moro* (N = 25); **Table S12.** Measurements for *Tetraclitella divisa* (N = 1); **Table S13.** Measurements for *Tetraclitella karandei* (N = 3); **Table S14.** Measurements for *Tesseropora rosea* (N = 15); **Table S15.** Measurements for *Tetraclita kuroshioensis* (N = 5); **Table S16.** Measurements for *Tetraclita squamosa* (N = 5); **Table S17.** Measurements for *Yamaguchiella coerulescens* (N = 25); **Table S18.** Measurements for *Neonrosella vitiata* (N = 4); **Table S19.** Measurements for *Newmanella spinosus* (N = 5); **Table S20.** Measurements for *Amphibalanus amphitrite* (N = 15); **Table S21.** Measurements for *Amphibalanus reticulatus* (N = 5); **Table S22.** Measurements for *Amphibalanus variegatus* (N = 10); **Table S23.** Measurements for *Amphibalanus zhujiangensis* (N = 11); **Table S24.** Measurements for *Amphibalanus* sp. (N = 4); **Table S25.** Measurements for *Megabalanus tintinnabulum* (N = 4); **Talbe S26.** Measurements for *Megabalanus zebra* (N = 8); **Table S27.** Kimura 2-parameter (K2P) distances of COI sequences between species for *Microeuraphia* sp.; **Table S28.** Kimura 2-parameter (K2P) distances of COI sequences between species for *Amphibalanus* sp. https://binary.pensoft.net/file/428616This dataset is made available under the Open Database License (http://opendatacommons.org/licenses/odbl/1.0/). The Open Database License (ODbL) is a license agreement intended to allow users to freely share, modify, and use this Dataset while maintaining this same freedom for others, provided that the original source and author(s) are credited.Pipit Pitriana, Luis Valente, Thomas von Rintelen, Diana S. Jones, Romanus E. Prabowo, Kristina von Rintelen
