# Supplementary material for: An annotated checklist and integrative biodiversity discovery of barnacles (Crustacea, Cirripedia) from the Moluccas, East Indonesia
Source: Zookeys. 2020 Jul 3;945:17–83. doi: 10.3897/zookeys.945.39044 (PMC7351798; doi:10.3897/zookeys.945.39044)
Supplement: Supplementary material 1 — Tables S1–S28, Figs S1–S13 [file zookeys-945-017-s001.pdf]

Supplementary material for

An annotated checklist and integrative biodiversity discovery of barnacles (Crustacea: Cirripedia) from the Moluccas, East Indonesia

Pipit Pitriana, Luis Valente, Thomas von Rintelen, Diana S. Jones, Romanus E. Prabowo & Kristina von Rintelen

Supplementary Tables 1-28, Supplementary Figs 1-13

Supplementary Table 1. Table of sample information for DNA samples sequenced for this study.

No.	Species name	Island	Internal code	Specimen voucher	Genbank number	
					COI	18S RNA
1	*Amphibalanus zhujiangensis*	Saparua	Bcl1716PM775	MZB Cru Cir 019-1	MK995334	MK981347
2	*A. zhujiangensis*	Sulawesi	Bcl4517PM1404	Bcl4517PM1404	MK995335	MK981348
3	*A. zhujiangensis*	Saparua	Bcl9016PM1222	MZB Cru Cir 021	MK995336	MK981349
4	*A. zhujiangensis*	Seram	Bcl3317PM1307	MZB Cru Cir 010-3	MK995337	MK981350
5	*A. zhujiangensis*	Sulawesi	Bcl4517PM1406	Bcl4517PM1406	MK995338	MK981351
6	*A. zhujiangensis*	Ambon	Bcl2316PM803	MZB Cru Cir 016-1	MK995339	MK981352
7	*A. zhujiangensis*	Sulawesi	Bcl 4517PM1405	Bcl 4517PM1405	MK995340	MK981353
8	*A. zhujiangensis*	Sulawesi	Bcl4517PM1407	Bcl4517PM1407	MK995341	MK981354
9	*Amphibalanus variegatus*	Saparua	Bcl5316PM1004	MZB Cru Cir 013-1	MK995342	-
10	*A. variegatus*	Saparua	X3PM922	MZB Cru Cir 015-2	MK995343	-
11	*A. variegatus*	Saparua	X2PM732	MZB Cru Cir 015-1	MK995344	-
12	*A. variegatus*	Saparua	Bcl4416PM953	MZB Cru Cir 013-2	MK995345	MK981355
13	*A. variegatus*	Sulawesi	Bcl7717PM1874	Bcl7717PM1874	MK995346	-
14	*Amphibalanus amphitrite*	Sulawesi	Bcl6317PM1730	Bcl6317PM1730	MK995347	-
15	*A. amphitrite*	Sulawesi	Bcl6317PM1780	Bcl6317PM1780	MK995348	-
16	*Amphibalanus* sp.	Ambon	Bcl1117PM125	MZB Cru Cir 136	MK995349	MK981356
17	*A.* sp.	Seram	Bcl4117PM1379	MZB Cru Cir 137-2	MK995350	MK981357
18	*A.* sp.	Ambon	Bcl4216PM937	MZB Cru Cir 135	MK995351	MK981358
19	*A.* sp.	Sulawesi	Bcl5217PM1550	Bcl5217PM1550	MK995352	MK981359
20	*A.* sp.	Seram	Bcl4117PM1378	MZB Cru Cir 137-1	MK995353	MK981360
21	*A.* sp.	Sulawesi	Bcl7517PM1857	Bcl7517PM1857	MK995354	MK981361
22	*Acasta* sp.	Sulawesi	Y0117PM1750	Y0117PM1750	-	MK981362
23	*Tetraclita singaporensis*	Sulawesi	Bcl5417PM1640	Bcl5417PM1640	MK995355	MK981363
24	*T. singaporensis*	Sulawesi	Bcl5717PM1663	Bcl5717PM1663	MK995356	MK981364
25	*T. singaporensis*	Sulawesi	Bcl5917PM1699	Bcl5917PM1699	MK995357	MK981365
26	*T. singaporensis*	Sulawesi	Bcl7617PM1867	Bcl7617PM1867	MK995358	MK981366
27	*T. singaporensis*	Sulawesi	Bcl7417PM1847	Bcl7417PM1847	MK995359	MK981367
28	*Tetraclita squamosa*	Saparua	Bcl8416PM1199	MZB Cru Cir 093-1	MK995360	MK981368
29	*T. squamosa*	Ambon	Bcl1517PM291	MZB Cru Cir 081-1	MK995361	MK981369
30	*T. squamosa*	Saparua	Bcl4916PM986	MZB Cru Cir 095-1	MK995362	MK981370
31	*T. squamosa*	Ambon	Bcl1717PM347	MZB Cru Cir 096-1	-	MK981371
32	*T. squamosa*	Ambon	Bcl0917PM120	MZB Cru Cir 092-1	-	MK981372
33	*T. squamosa*	Ambon	Bcl3017PM720	MZB Cru Cir 099-1	-	MK981373
34	*T. squamosa*	Sulawesi	Bcl6517PM1769	Bcl6517PM1769	-	MK981374
35	*Tetraclita kuroshioensis*	Ambon	Bcl0317PM13	MZB Cru Cir 097	MK995363	MK981375

36	*T. kuroshioensis*	Ambon	Bcl0717PM39	MZB Cru Cir 098-1	MK995364	MK981376
37	*T. kuroshioensis*	Sulawesi	Bcl6417PM1740	Bcl6417PM1740	MK995365	MK981377
38	*T. kuroshioensis*	Sulawesi	Bcl4417PM1403	Bcl4417PM1403	MK995366	MK981378
39	*T. kuroshioensis*	Saparua	Bcl7916PM1180	MZB Cru Cir 100	MK995367	MK981379
40	*Newmanella spinosus*	Sulawesi	Bcl4817PM1441	Bcl4817PM1441	MK995368	-
41	*Yamaguchiella coerulescens*	Ambon	Bcl1216PM494	MZB Cru Cir 126-1	-	MK981381
42	*Neonrosella vitiata*	Ambon	X1PM723	MZB Cru Cir 132-1	-	MK981384
43	*Tesseropora rosea*	Ambon	Bcl0216PM27	MZB Cru Cir 075-1	MK995370	-
44	*Dosima fascicularis*	Ambon	Bcl2417PM500	MZB Cru Cir 048-1	MK995371	MK981385
45	*Heteralepas japonica*	Deep-sea	Bcl9416PM1911	MZB Cru Cir 050-1	MK995372	MK981386
46	*Lepas anserifera*	Ambon	Bcl2317PM495	MZB Cru Cir 059-1	MK995373	-
47	*L. anserifera*	Seram	Bcl3417PM1322	MZB Cru Cir 061-1	MK995374	MK981387
48	*L. anserifera*	Seram	Bcl3517PM1323	MZB Cru Cir 062-1	MK995375	MK981388
49	*Nesochthamalus intertextus*	Ambon	Bcl2217PM429	MZB Cru Cir 070-1	MK995376	MK981389
50	*Capitulum mitella*	Ambon	Bcl0617PM34	MZB Cru Cir 032-1	-	MK981390
51	*Chthamalus moro*	Ambon	Bcl0417PM14	MZB Cru Cir 039-1	MK995377	MK981391
52	*C. moro*	Saparua	Bcl4116PM902	MZB Cru Cir 046-1	MK995378	-
53	*C. moro*	Saparua	Bcl3316PM872	MZB Cru Cir 045-1	MK995379	MK981392
54	*C. moro*	Ambon	Bcl1417PM289	MZB Cru Cir 036-1	MK995380	-
55	*C. moro*	Pombo	Bcl8816PM1217	MZB Cru Cir 043-1	MK995381	MK981393
56	*C. moro*	Seram	Bcl4317PM1388	MZB Cru Cir 047-1	MK995382	MK981394
57	*C. moro*	Ambon	Bcl1217PM243	MZB Cru Cir 040-1	MK995383	MK981395
58	*C. moro*	Saparua	Bcl5916PM1052	MZB Cru Cir 044-2	MK995384	MK981396
59	*C. moro*	Pombo	Bcl8816PM1218	MZB Cru Cir 043-2	MK995385	MK981397
60	*C. moro*	Saparua	Bcl2116PM787	MZB Cru Cir 044-1	MK995386	MK981398
61	*C. moro*	Ambon	Bcl2717PM613	MZB Cru Cir 041-1	MK995387	MK981399
62	*C. moro*	Seram	Bcl4317PM1389	MZB Cru Cir 047-2	MK995388	MK981400
63	*Microeuraphia* sp.	Seram	Bcl3917PM1358	MZB Cru Cir 138-1	MK995389	MK981401
64	*M.* sp.	Seram	Bcl3917PM1359	MZB Cru Cir 138-2	MK995390	MK981402
65	*M.* sp.	Sulawesi	Bcl4917PM1510	Bcl4917PM1510	MK995391	MK981403
66	*M.* sp.	Sulawesi	Bcl5517PM1641	Bcl5517PM1641	MK995392	MK981404
67	*M.* sp.	Sulawesi	X4PM1494	X4PM1494	MK995393	MK981405
68	*M.* sp.	Sulawesi	Bcl5317PM1625	Bcl5317PM1625	MK995394	-
69	*M.* sp.	Sulawesi	Bcl6217PM1725	Bcl6217PM1725	MK995395	MK981406
70	*M.* sp.	Sulawesi	X5PM1581	X5PM1581	MK995396	MK981407

Supplementary Table 2. Measurements for *Heteralepas japonica* (n=25).

**Parameter***	**Range (mm)**	**Mean ± SD (mm)**	**Median (mm)**
A	11.9-18.6	15.1 ± 0.9	14.5
B	8.7-15.3	12.2 ± 2.5	12.2
C	7.7-27.6	16.3 ± 2.7	11.1
D	3.2-9.4	6.5 ± 0.8	4.4
E	2.0-3.0	2.5 ± 0.0	2.0
F	7.0-13.3	9.1 ± 0.2	7.6
G	5.1-10.4	7.3 ± 1.0	7.2
A/B	1.1-1.5	1.2 ± 0.2	1.2
A/C	0.6-1.7	1.0 ± 0.4	1.4
A/D	1.6-3.9	2.5 ± 0.8	3.3
A/E	4.1-9.2	6.3 ± 0.5	7.2
A/F	1.4-2.1	1.7 ± 0.1	1.9
A/G	1.6-2.5	2.1 ± 0.4	2.0
B/C	0.5-1.5	0.7 ± 0.5	1.2
B/D	1.4-3.6	2.0 ± 1.1	2.8
B/E	2.9-6.9	5.1 ± 1.2	6.1
B/F	1.1-1.8	1.4 ± 0.3	1.6
B/G	1.3-2.1	1.7 ± 0.6	1.7
C/D	1.3-4.8	2.7 ± 0.2	2.5
C/E	3.8-12.5	6.5 ± 1.3	5.5
C/F	0.9-3.1	1.8 ± 0.4	1.5
C/G	1.3-3.9	2.2 ± 0.2	1.5
D/E	1.7-4.1	2.7 ± 0.4	2.2
D/F	0.4-1.1	0.7 ± 0.1	0.6
D/G	0.6-1.3	0.9 ± 0.0	0.6
E/F	0.2-0.4	0.3 ± 0.0	0.3
E/G	0.2-0.5	0.4 ± 0.0	0.3
F/G	0.9-1.8	1.3 ± 0.2	1.1

*A=Capitulum height; B=Capitulum width; C=Peduncle length; D=Orifice height; E=Number of crests; F=Capitulum thickness; G=Peduncle width.

Supplementary Table 3. Measurements for *Dosima fascicularis* (n=6).

Parameter*	Range (mm)	Mean ± SD (mm)	Median (mm)
TH	11.7-19.5	16.0 ± 0.8	19.0
CH	10.5-17.1	13.7 ± 2.5	15.3
DBC	4.0-6.8	5.5 ± 0.2	6.6
CS	6.5-10.2	8.3 ± 1.2	9.4
LS	8.1-11.1	9.6 ± 0.8	10.6
WS	5.4-7.3	6.9 ± 1.0	8.0
LT	6.2-11.1	8.6 ± 1.6	10.0
WT	2.7-4.4	3.6 ± 0.8	3.8
TH/CH	1.1-1.2	1.2 ± 0.2	1.3
TH/DBC	2.6-3.2	2.9 ± 0.0	2.9
TH/CS	1.8-2.2	1.9 ± 0.2	2.0
TH/LS	1.4-1.9	1.7 ± 0.1	1.8
TH/WS	2.2-2.6	2.3 ± 0.2	2.9
TH/LT	1.7-2.1	1.9 ± 0.2	1.9
TH/WT	2.9-5.7	4.5 ± 0.9	5.1
CH/DBC	2.1-2.7	2.5 ± 0.3	2.3
CH/CS	1.6-1.7	1.6 ± 0.2	1.6
CH/LS	1.3-1.5	1.4 ± 0.1	1.5
CH/WS	1.9-2.1	2.0 ± 0.1	1.9
CH/LT	1.5-1.7	1.6 ± 0.0	1.5
CH/WT	2.6-4.2	3.8 ± 0.2	4.1
DBC/CS	0.6-0.8	0.7 ± 0.1	0.7
DBC/LS	0.5-0.7	0.6 ± 0.0	0.6
DBC/WS	0.7-0.9	0.8 ± 0.1	0.8
DBC/LT	0.6-0.7	0.6 ± 0.1	0.7
DBC/WT	1.0-2.0	1.5 ± 0.3	1.8
CS/LS	0.8-0.9	0.9 ± 0.0	0.9
CS/WS	1.2-1.2	1.2 ± 0.0	1.2
CS/LT	0.9-1.1	1.0 ± 0.0	0.9
CS/WT	1.6-2.7	2.3 ± 0.2	2.5
LS/WS	1.3-1.5	1.4 ± 0.1	1.3
LS/LT	1.0-1.3	1.1 ± 0.1	1.1
LS/WT	2.1-3.1	2.7 ± 0.4	2.8
WS/LT	0.7-0.9	0.8 ± 0.0	0.8
WS/WT	1.3-2.3	1.9 ± 0.2	2.1
LT/WT	1.5-2.8	2.4 ± 0.2	2.6

*TH=Total height; CH=Capitulum height; DBC=Diameter of the base of capitulum; LS=Scutum length; WS=Scutum width; LT=Tergum length; WT=Tergum width.

Supplementary Table 4. Measurements for *Lepas anserifera* (n=25).

Parameter*	Range (mm)	Mean ± SD (mm)	Median (mm)
TH	14.2-31.9	21.1 ± 4.0	22.2
CH	8.1-14.8	12.0 ± 1.3	13.0
DBC	2.0-7.2	4.0 ± 0.6	4.0
CS	5.2-12.4	8.1 ± 1.5	8.5
LS	6.3-12.3	9.6 ± 1.3	9.6
WS	4.7-11.3	7.3 ± 1.2	7.7
LT	4.3-9.6	7.7 ± 0.6	7.9
WT	1.6-3.2	2.5 ± 0.2	2.7
TH/CH	1.3-2.5	1.8 ± 0.1	1.7
TH/DBC	2.7-7.2	5.5 ± 0.2	5.6
TH/CS	1.3-3.8	2.6 ± 0.0	2.6
TH/LS	1.3-3.1	2.2 ± 0.1	2.3
TH/WS	1.5-4.1	2.9 ± 0.0	2.9
TH/LT	2.1-5.5	2.8 ± 0.3	2.8
TH/WT	5.8-13.8	8.5 ± 1.0	8.1
CH/DBC	1.7-4.7	3.2 ± 0.1	3.3
CH/CS	1.0-1.7	1.5 ± 0.1	1.5
CH/LS	1.0-1.4	1.3 ± 0.1	1.4
CH/WS	1.1-2.0	1.7 ± 0.1	1.7
CH/LT	1.3-2.2	1.6 ± 0.0	1.7
CH/WT	3.9-5.9	4.8 ± 0.2	4.7
DBC/CS	0.3-0.8	0.5 ± 0.0	0.5
DBC/LS	0.3-0.7	0.4 ± 0.0	0.4
DBC/WS	0.4-0.9	0.6 ± 0.0	0.5
DBC/LT	0.3-0.9	0.5 ± 0.0	0.5
DBC/WT	0.9-3.3	1.6 ± 0.1	1.4
CS/LS	0.7-1.0	0.9 ± 0.0	0.9
CS/WS	1.0-1.2	1.1 ± 0.0	1.1
CS/LT	0.9-1.8	1.1 ± 0.1	1.1
CS/WT	2.4-5.5	3.3 ± 0.4	3.1
LS/WS	1.1-1.8	1.3 ± 0.0	1.3
LS/LT	1.1-2.0	1.3 ± 0.1	1.2
LS/WT	3.6-5.6	3.9 ± 0.3	3.5
WS/LT	0.8-1.6	1.0 ± 0.1	1.0
WS/WT	2.1-5.1	2.9 ± 0.3	2.8
LT/WT	2.4-4.3	3.1 ± 0.1	2.9

*TH=Total height; CH=Capitulum height; DBC=Diameter of the base of capitulum; LS=Scutum length; WS=Scutum width; LT=Tergum length; WT=Tergum width.

Supplementary Table 5. Measurements for *Capitulum mitella* (n=25).

**Parameter***	**Range (mm)**	**Mean ± SD (mm)**	**Median (mm)**
TH	10.7-7.1	24.7 ± 10.7	26.7
CH	7.0- 4.6	14.9 ± 5.4	16.6
RC	5.8-27.3	15.6 ± 6.3	17.1
RT	5.5-24.2	13.7 ± 5.7	15.2
DBC	4.5-20.4	11.0 ± 10.8	10.6
TH/CH	1.2-2.2	1.6 ± 1.6	1.6
TH/RC	1.0-2.2	1.6 ± 0.3	1.6
TH/RT	1.3-2.5	1.8 ± 0.3	1.8
TH/DBC	1.5-2.9	2.3 ± 0.4	2.3
CH/RC	0.7-1.2	1.0 ± 0.1	1.0
CH/RT	0.8-1.4	1.1 ± 0.1	1.1
CH/DBC	0.9-1.9	1.4 ± 1.4	1.4
RC/RT	0.9-1.3	1.2 ± 1.2	1.2
RC/DBC	1.3-1.7	1.4 ± 0.1	1.4
RT/DBC	1.0-1.5	1.3 ± 0.1	1.2

*TH=Total height; CH=Capitulum height; RC=Distance from rostrum to carina; RT=Rostrum height; DBC=Capitulum diameter.

Supplementary Table 6. Measurements for *Pseudoctomeris sulcata* (n=2).

**Parameter***	**Range (mm)**	**Mean ± SD (mm)**	**Median (mm)**
LB	16.2-16.7	16.4 ± 0.3	16.4
LO	5.5-7.9	6.7 ± 1.8	6.7
H	7.4-7.7	7.6 ± 0.2	7.6
WO	4.3-6.1	5.2 ± 1.3	5.2
WB	11.5-15.0	13.3 ± 2.5	13.3
LB/LO	2.0-3.1	2.5 ± 0.7	2.6
LB/H	2.1-2.2	2.2 ± 0.1	2.2
LB/WO	2.7-3.9	3.3 ± 0.9	3.3
LB/WB	1.1-1.5	1.3 ± 0.3	1.3
LO/H	0.7-1.0	0.9 ± 0.2	0.9
LO/WO	1.3-1.3	1.3 ± 0.0	1.3
LO/WB	0.5-0.5	0.5 ± 0.0	0.5
WO/H	0.6-0.8	0.7 ± 0.2	0.7
WB/H	1.5-1.9	1.8 ± 0.3	1.7
WB/WO	2.5-2.7	2.6 ± 0.2	2.6

*LB=Basal lenght; LO=Orifice lenght; H=Carinal heigth; WO=Orifice width; WB=Basal width.

Supplementary Table 7. Measurements for *Hexechamaesipho pilsbryi* (n=10).

**Parameter***	**Range (mm)**	**Mean ± SD (mm)**	**Median (mm)**
LB	8.9-17.0	14.1 ± 3.4	14.6
LO	4.2-6.9	5.7 ± 0.5	5.4
H	1.0-3.7	1.7 ± 0.1	1.7
WO	3.6-5.6	4.3 ± 0.0	3.7
WB	10.3-16.4	13.4 ± 0.3	12.6
LB/LO	1.7-4.5	2.3 ± 0.4	2.7
LB/H	0.8-1.3	8.4 ± 2.6	8.7
LB/WO	1.4-6.6	3.3 ± 0.9	3.9
LB/WB	0.8-1.4	1.1 ± 0.3	1.2
LO/H	1.4-6.6	3.4 ± 0.5	3.2
LO/WO	1.0-1.7	1.3 ± 0.1	1.5
LO/WB	0.3-0.5	0.4 ± 0.1	0.4
WO/H	1.4-3.9	2.6 ± 0.2	2.2
WB/H	3.1-16.6	8.0 ± 0.3	7.4
WB/WO	2.2-4.3	3.1 ± 0.1	3.4

*LB=Basal lenght; LO=Orifice lenght; H=Carinal heigth; WO=Orifice width; WB=Basal width.

Supplementary Table 8. Measurements for *Nesochthamalus intertextus* (n=10).

**Parameter***	**Range (mm)**	**Mean ± SD (mm)**	**Median (mm)**
LB	8.9-12.1	10.5 ± 0.2	11.0
LO	3.0-4.9	3.8 ± 0.5	4.6
H	1.3-3.1	2.2 ± 0.3	2.2
WO	2.5-3.9	3.3 ± 0.3	3.7
WB	6.9-10.3	9.0 ± 0.4	10.0
LB/LO	2.2-3.5	2.8 ± 0.3	2.4
LB/H	3.5-8.1	5.0 ± 0.6	5.1
LB/WO	2.8-3.9	3.2 ± 0.3	3.0
LB/WB	0.9-1.4	1.2 ± 0.1	1.1
LO/H	1.3-2.3	1.8 ± 0.0	2.1
LO/WO	1.0-1.4	1.2 ± 0.2	1.2
LO/WB	0.4-0.6	0.4 ± 0.1	0.5
WO/H	1.2-2.1	1.5 ± 0.4	1.7
WB/H	2.6-6.0	4.3 ± 0.8	4.7
WB/WO	2.2-3.8	2.8 ± 0.1	2.7

*LB=Basal length; LO=Orifice length; H=Carinal height; WO=Orifice width; WB=Basal width.

Supplementary Table 9. Measurements for *Euraphia hembeli* (n=1).

Parameter*	Value (mm)
LB	37.4
LO	18.3
H	14.3
WO	15.2
WB	28.6
LB/LO	2.1
LB/H	2.6
LB/WO	2.5
LB/WB	1.3
LO/H	1.3
LO/WO	1.2
LO/WB	0.6
WO/H	1.1
WB/H	2.0
WB/WO	1.9

*LB=Basal length; LO=Orifice length; H=Carinal height; WO=Orifice width; WB=Basal width.

Supplementary Table 10. Measurements for *Microeuraphia* sp. (n=2).

Parameter*	Range (mm)	Mean ± SD (mm)	Median (mm)
LB	3.6-9.9	6.8 ± 4.4	6.8
LO	1.5-4.5	3.0 ± 2.2	3.0
H	1.2-2.2	1.7 ± 0.8	1.7
WO	0.7-3.6	2.1 ± 2.0	2.1
WB	3.0-9.1	6.0 ± 4.3	6.0
LB/LO	2.2-2.5	2.3 ± 0.2	2.3
LB/H	3.1-4.3	3.7 ± 0.9	3.7
LB/WO	2.8-5.0	3.9 ± 1.5	3.9
LB/WB	1.1-1.2	1.1 ±0.1	1.1
LO/H	1.2-2.0	1.6 ± 0.5	1.6
LO/WO	1.3-2.0	1.6 ± 0.5	1.6
LO/WB	0.4-0.5	0.5 ± 0.0	0.5
WO/H	0.6-1.6	1.1 ± 0.7	1.1
WB/H	2.6-4.0	3.3 ± 1.0	3.3
WB/WO	2.1-4.0	3.3 ± 1.2	3.3

*LB=Basal lenght; LO=Orifice lenght; H=Carinal height; WO=Orifice width; WB=Basal width.

Supplementary Table 11. Measurements for *Chthamalus moro* (n=25).

**Parameter***	**Range (mm)**	**Mean ± SD (mm)**	**Median (mm)**
LB	2.4-5.1	3.6 ± 0.8	4.5
LO	1.0-3.4	1.7 ± 1.5	2.4
H	0.8-1.7	1.3 ± 0.0	1.7
WO	0.7-1.7	1.2 ± 0.1	1.1
WB	1.4-4.1	2.8 ± 0.5	3.7
LB/LO	1.4-3.3	2.3 ± 1.0	2.2
LB/H	2.0-4.4	2.9 ± 0.5	2.7
LB/WO	1.7-4.3	3.2 ± 0.4	4.1
LB/WB	1.1-1.7	1.3 ± 0.0	1.2
LO/H	0.8-2.4	1.4 ± 0.9	1.4
LO/WO	0.8-2.9	1.5 ± 1.1	2.1
LO/WB	0.4-1.2	0.6 ± 0.3	0.6
WO/H	0.6-1.4	0.9 ± 0.1	0.7
WB/H	1.2-3.2	2.3 ± 0.3	2.2
WB/WO	1.0-3.5	2.5 ± 0.2	3.4

*LB=Basal length; LO=Orifice length; H=Carinal height; WO=Orifice width; WB=Basal width.

Supplementary Table 12. Measurements for *Tetraclitella divisa* (n=1).

**Parameter**	**Value (mm)**
LB	11.1
LO	4.9
H	2.4
WO	3.5
WB	9.7
LB/LO	2.3
LB/H	4.7
LB/WO	3.1
LB/WB	1.2
LO/H	2.1
LO/WO	1.4
LO/WB	0.5
WO/H	1.5
WB/H	4.1
WB/WO	2.7

*LB=Basal length; LO=Orifice length; H=Carinal height; WO=Orifice width; WB=Basal width.

Supplementary Table 13. Measurements for *Tetraclitella karandei* (n=3).

**Parameter***	**Range (mm)**	**Mean ± SD (mm)**	**Median (mm)**
LB	10.1-17.4	14.2 ± 3.7	12.7
LO	3.8-5.6	4.5 ± 3.7	4.0
H	0.4-0.7	0.6 ± 0.1	0.7
WO	2.7-5.2	3.8 ± 0.6	3.2
WB	8.2-18.2	13.4 ± 4.0	11.1
LB/LO	2.7-3.6	3.1 ± 0.7	3.1
LB/H	13.6-48.3	29.3 ± 8.7	19.7
LB/WO	3.3-4.3	3.8 ± 0.4	4.0
LB/WB	1.0-1.2	1.1 ± 0.1	1.2
LO/H	5.1-15.4	9.2 ± 1.5	6.2
LO/WO	1.1-1.4	1.2 ± 0.2	1.3
LO/WB	0.3-0.5	0.4 ± 0.1	0.4
WO/H	3.7-14.5	8.1 ± 1.7	4.9
WB/H	11.1-50.5	28.4 ± 8.8	17.3
WB/WO	3.0-3.9	3.5 ± 0.6	3.5

*LB=Basal length; LO=Orifice length; H=Carinal height; WO=Orifice width; WB=Basal width.

Supplementary Table 14. Measurements for *Tesseropora rosea* (n=15).

**Parameter***	**Range (mm)**	**Mean ± SD (mm)**	**Median (mm)**
LB	9.7-25.6	16.6 ± 7.5	19.1
LO	2.9-7.8	6.1 ± 0.1	6.0
H	4.4-13.0	8.4 ± 2.8	9.0
WO	2.3-6.9	5.3 ± 0.5	5.0
WB	9.7-24.5	15.2 ± 10.5	17.1
LB/LO	1.5-4.9	2.8 ± 1.2	3.2
LB/H	1.4-3.6	2.0 ± 0.2	2.1
LB/WO	1.8-5.4	3.3 ± 1.9	3.9
LB/WB	0.8-1.4	1.1 ± 0.3	1.2
LO/H	0.3-1.5	0.8 ± 0.2	0.7
LO/WO	1.1-1.3	1.2 ± 0.2	1.2
LO/WB	0.2-0.8	0.4 ± 0.3	0.4
WO/H	0.3-1.4	0.7 ± 0.3	0.6
WB/H	1.2-4.2	1.9 ± 0.6	1.8
WB/WO	1.7-5.3	3.0 ± 2.5	3.5

*LB=Basal length; LO=Orifice length; H=Carinal height; WO=Orifice width; WB=Basal width.

Supplementary Table 15. Measurements for *Tetraclita kuroshioensis* (n=5).

Parameter*	Range (mm)	Mean ± SD (mm)	Median (mm)
LB	12.1-21.6	17.2 ± 5.8	16.2
LO	3.2-5.3	3.9 ± 0.2	3.8
H	7.3-10.4	8.4 ± 1.1	8.0
WO	2.4-4.2	2.9 ± 0.3	2.6
WB	18.1-21.8	17.7 ± 5.0	14.5
LB/LO	3.3-5.4	4.5 ± 1.3	4.3
LB/H	1.4-2.8	2.1 ± 1.0	2.1
LB/WO	4.4-8.4	6.0 ± 2.9	6.4
LB/WB	0.8-1.1	1.0 ± 0.0	1.1
LO/H	0.4- 0.5	0.5 ± 0.1	0.5
LO/WO	1.2-1.6	1.3 ± 0.2	1.5
LO/WB	0.2-0.3	0.2 ± 0.1	0.3
WO/H	0.3-0.4	0.4 ± 0.0	0.3
WB/H	1.3-2.5	2.1 ± 0.9	1.9
WB/WO	3.9-7.5	6.1 ± 2.5	5.7

*LB=Basal length; LO=Orifice length; H=Carinal height; WO=Orifice width; WB=Basal width.

Supplementary Table 16. Measurements for *Tetraclita squamosa* (n=5).

Parameter*	Range (mm)	Mean ± SD (mm)	Median (mm)
LB	20.1-30.2	25.4 ± 3.4	27.6
LO	4.4-7.9	6.1 ± 1.7	6.7
H	11.9-14.2	12.9 ± 0.3	12.5
WO	3.4-7.2	5.3 ± 1.4	5.7
WB	19.3-28.3	24.8 ± 0.2	26.0
LB/LO	3.8-4.6	4.2 ± 0.6	4.2
LB/H	1.6-2.4	2.0 ± 0.2	2.2
LB/WO	4.2-5.9	4.9 ± 0.6	4.9
LB/WB	0.9-1.2	1.0 ± 0.1	1.1
LO/H	0.4-0.6	0.5 ± 0.1	0.5
LO/WO	1.0-1.3	1.2 ± 0.0	1.2
LO/WB	0.2-0.3	0.3 ± 0.1	0.3
WO/H	0.3-0.5	0.4 ± 0.1	0.5
WB/H	1.6-2.1	1.9 ± 0.0	2.1
WB/WO	3.9-5.7	4.9 ± 1.1	4.7

*LB=Basal length; LO=Orifice length; H=Carinal height; WO=Orifice width; WB=Basal width.

Supplementary Table 17. Measurements for *Yamaguchiella coerulescens* (n=25).

**Parameter***	**Range (mm)**	**Mean ± SD (mm)**	**Median (mm)**
LB	8.3 – 29.0	19.5 ± 12.6	17.2
LO	4.2 – 11.5	7.1 ± 3.3	6.8
H	5.7 – 17.7	11.0 ± 5.2	9.3
WO	3.2 – 11.8	7.1 ± 4.1	7.4
WB	8.5 – 27.8	19.7 ±12.0	18.6
LB/LO	1.8 – 3.5	2.8 ± 0.7	2.3
LB/H	1.4 – 2.7	1.8 ±0.4	1.7
LB/WO	1.9 – 3.7	2.8 ± 0.5	2.2
LB/WB	0.8 – 1.3	0.1 ± 0.1	0.9
LO/H	0.5 – 1.0	0.7 ± 0.1	0.8
LO/WO	0.9 – 1.3	1.0 ± 0.1	0.9
LO/WB	0.3 – 0.5	0.4 ± 0.1	0.4
WO/H	0.5 – 0.9	0.7 ± 0.0	0.8
WB/H	1.4 – 2.9	1.8 ± 0.2	1.9
WB/WO	2.1 – 3.8	2.8 ± 0.3	2.4

*LB=Basal length; LO=Orifice length; H=Carinal height; WO=Orifice width; WB=Basal width.

Supplementary Table 18. Measurements for *Neonrosella vitiata* (n=4).

**Parameter***	**Range (mm)**	**Mean ± SD (mm)**	**Median (mm)**
LB	15.4-22.8	18.9 ± 5.3	19.1
LO	5.2-6.9	5.9 ± 0.5	5.8
H	5.3-6.9	5.8 ± 0.3	5.5
WO	5.0-5.4	5.1 ± 0.2	5.2
WB	13.8-22.8	18.8 ± 6.3	18.3
LB/LO	2.8-3.7	3.2 ± 0.6	3.3
LB/H	2.9-4.0	3.3 ± 0.8	3.4
LB/WO	3.1-4.3	3.7 ± 0.9	3.7
LB/WB	0.9-1.1	1.0 ± 0.1	1.1
LO/H	1.0-1.1	1.0 ± 0.0	1.0
LO/WO	1.1-1.3	1.2 ± 0.0	1.1
LO/WB	0.3-0.4	0.3 ± 0.1	0.3
WO/H	0.8-0.9	0.9 ± 0.0	0.9
WB/H	2.6-4.0	3.2 ± 1.0	3.3
WB/WO	2.8-4.3	3.7 ± 1.1	3.5

*LB=Basal length; LO=Orifice length; H=Carinal height; WO=Orifice width; WB=Basal width.

Supplementary Table 19. Measurements for *Newmanella spinosus* (n=5).

Parameter*	Range (mm)	Mean ± SD (mm)	Median (mm)
LB	17.4-21.0	19.3 ± 1.1	18.2
LO	5.3-7.2	6.5 ± 0.7	5.8
H	6.8-8.9	8.1 ± 1.5	7.8
WO	5.0-6.7	5.9 ± 0.2	5.1
WB	15.9-20.5	18.8 ± 2.5	17.6
LB/LO	2.7-3.3	3.0 ± 0.2	3.2
LB/H	2.0-2.8	2.4 ± 0.6	2.4
LB/WO	2.9-3.6	3.3 ± 0.1	3.5
LB/WB	1.0-1.1	1.0 ± 0.1	1.0
LO/H	0.6-0.9	0.8 ± 0.2	0.8
LO/WO	1.0-1.2	1.1 ± 0.1	1.1
LO/WB	0.3-0.4	0.4 ± 0.0	0.3
WO/H	0.6-0.8	0.7 ± 0.2	0.7
WB/H	1.8-2.9	2.4 ± 0.8	2.3
WB/WO	2.9-3.7	3.2 ± 0.4	3.4

*LB=Basal length; LO=Orifice length; H=Carinal height; WO=Orifice width; WB=Basal width.

Supplementary Table 20. Measurements for *Amphibalanus amphitrite* (n=15).

Parameter*	Range (mm)	Mean ± SD (mm)	Median (mm)
LB	3.1-17.8	10.3 ± 1.2	9.2
LO	1.5-8.1	5.1 ± 1.5	4.0
H	2.1-10.8	5.4 ± 0.4	4.7
WO	1.5-5.4	3.8 ± 1.2	2.9
WB	2.8-17.6	8.6 ± 1.6	6.5
LB/LO	1.4-3.3	2.0 ± 0.7	2.6
LB/H	1.2-5.8	2.0 ± 0.1	2.0
LB/WO	1.7-4.9	2.2 ± 1.2	3.7
LB/WB	0.8-2.7	1.2 ± 0.2	1.5
LO/H	0.6-3.2	1.0 ± 0.3	0.8
LO/WO	0.9-1.6	1.3 ± 0.1	1.4
LO/WB	0.3-1.1	0.6 ± 0.1	0.6
WO/H	0.4-2.0	0.8 ± 0.2	0.6
WB/H	0.6-4.7	1.7 ± 0.2	1.4
WB/WO	1.4-3.5	2.3 ± 0.5	2.5

*LB=Basal length; LO=Orifice length; H=Carinal height; WO=Orifice width; WB=Basal width.

Supplementary Table 21. Measurements for *Amphibalanus reticulatus* (n=5).

**Parameter***	**Range (mm)**	**Mean ± SD (mm)**	**Median (mm)**
LB	7.7-16.3	12.1 ± 2.7	10.7
LO	3.5-8.9	6.8 ± 1.2	5.7
H	3.2-10.5	6.9 ± 0.9	5.2
WO	2.5-7.1	4.9 ± 0.8	3.5
WB	2.9-15.5	10.0 ± 2.4	9.6
LB/LO	1.5-2.5	1.8 ± 0.1	1.9
LB/H	1.3-3.2	1.8 ± 0.2	2.0
LB/WO	1.8-3.5	2.5 ± 0.1	3.1
LB/WB	0.9-4.9	1.4 ± 0.0	1.1
LO/H	0.8-1.4	1.0 ± 0.0	1.1
LO/WO	1.1-1.9	1.4 ± 0.1	1.7
LO/WB	0.5-3.0	0.8 ± 0.0	0.7
WO/H	0.5-1.0	0.7 ± 0.0	0.7
WB/H	0.3-2.5	1.5 ± 0.2	1.8
WB/WO	0.4-3.0	2.1 ± 0.1	2.7

*LB=Basal length; LO=Orifice length; H=Carinal height; WO=Orifice width; WB=Basal width.

Supplementary Table 22. Measurements for *Amphibalanus variegatus* (n=10).

**Parameter***	**Range (mm)**	**Mean ± SD (mm)**	**Median (mm)**
LB	8.3-11.8	10.3 ± 1.1	9.3
LO	4.8-8.8	6.4 ± 0.4	5.2
H	4.3-8.4	5.7 ± 0.8	5.1
WO	3.4-5.3	4.2 ± 0.3	3.9
WB	6.9-10.4	9.1 ± 1.4	8.3
LB/LO	1.3-2.0	1.7 ± 0.1	1.8
LB/H	1.4-2.1	1.9 ± 0.1	1.8
LB/WO	2.2-2.8	2.5 ± 0.1	2.4
LB/WB	1.0-1.2	1.1 ± 0.1	1.1
LO/H	0.9-1.4	1.1 ± 0.1	1.0
LO/WO	1.3-1.8	1.5 ± 0.0	1.3
LO/WB	0.6-0.9	0.7 ± 0.1	0.6
WO/H	0.6-0.9	0.8 ± 0.1	0.8
WB/H	1.2-1.9	1.7 ± 0.0	1.6
WB/WO	1.9-2.4	2.2 ± 0.2	2.1

*LB=Basal length; LO=Orifice length; H=Carinal height; WO=Orifice width; WB=Basal width.

Supplementary Table 23. Measurements for *Amphibalanus zhujiangensis* (n=11).

**Parameter***	**Range (mm)**	**Mean ± SD (mm)**	**Median (mm)**
LB	5.8-21.6	12.6 ± 7.9	13.7
LO	3.0-10.0	6.5 ± 3.1	6.1
H	2.8-16.5	8.7 ± 4.1	6.9
WO	2.6-7.6	4.9 ± 1.8	4.8
WB	4.8-19.2	10.5 ± 3.7	8.8
LB/LO	1.2-2.4	1.9 ± 0.2	2.2
LB/H	0.9-2.1	1.5 ± 0.1	2.0
LB/WO	2.0-3.4	2.5 ± 0.7	2.6
LB/WB	1.0-1.7	1.2 ± 0.3	1.4
LO/H	0.5-1.1	0.8 ± 0.1	0.9
LO/WO	1.0-1.7	1.3 ± 0.2	1.2
LO/WB	0.5-0.9	0.7 ± 0.1	0.7
WO/H	0.4-1.1	0.6 ± 0.2	0.8
WB/H	0.7-1.8	1.3 ± 0.4	1.5
WB/WO	1.6-3.1	2.1 ± 0.1	1.8

*LB=Basal length; LO=Orifice length; H=Carinal height; WO=Orifice width; WB=Basal width.

Supplementary Table 24. Measurements for *Amphibalanus* sp. (n=4).

**Parameter***	**Range (mm)**	**Mean ± SD (mm)**	**Median (mm)**
LB	7.4-12.2	10.8 ± 2.3	11.9
LO	3.6-8.3	6.0 ± 1.9	6.9
H	5.5-9.4	7.1 ± 1.6	6.8
WO	2.6-5.9	4.3 ± 1.4	4.3
WB	6.3-11.8	9.4 ± 2.3	9.8
LB/LO	1.4-2.1	1.9 ± 0.3	2.0
LB/H	1.2-1.8	1.5 ± 0.3	1.5
LB/WO	1.9-3.2	2.6 ± 0.5	2.7
LB/WB	1.0-1.3	1.2 ± 0.1	1.2
LO/H	0.7-0.9	0.8 ± 0.1	0.9
LO/WO	1.3-1.6	1.4 ± 0.1	1.4
LO/WB	0.6-0.7	0.6 ± 0.1	0.6
WO/H	0.5-0.7	0.6 ± 0.1	0.6
WB/H	1.1-1.5	1.3 ± 0.1	1.3
WB/WO	2.0-2.6	2.3 ± 0.3	2.2

*LB=Basal lenght; LO=Orifice lenght; H=Carinal height; WO=Orifice width; WB=Basal width.

Supplementary Table 25. Measurements for *Megabalanus tintinnabulum* (n=4).

Parameter*	Range (mm)	Mean ± SD (mm)	Median (mm)
LB	26.0-49.2	37.3 ± 1.1	1.1
LO	13.7-16.3	15.2 ± 0.1	0.1
H	20.1-49.4	39.3 ± 16.5	16.5
WO	10.4-15.9	13.1 ± 3.7	3.7
WB	29.0-43.1	37.4 ± 9.9	10.0
LB/LO	1.6-3.1	2.5 ± 0.1	0.1
LB/H	0.6-1.9	1.1 ± 0.8	0.8
LB/WO	2.3-3.7	2.9 ± 0.9	0.9
LB/WB	0.8-1.3	1.0 ± 0.3	0.3
LO/H	0.3-0.8	0.4 ± 0.3	0.3
LO/WO	0.9-1.4	1.2 ± 0.4	0.4
LO/WB	0.4-0.5	0.4 ± 0.1	0.1
WO/H	0.3-0.5	0.4 ± 0.1	0.1
WB/H	0.8-1.4	1.0 ± 0.3	0.3
WB/WO	2.6-3.1	2.9 ± 0.0	0.0

*LB=Basal lenght; LO=Orifice lenght; H=Carinal height; WO=Orifice width; WB=Basal width.

Supplementary Table 26. Measurements for *Megabalanus zebra* (n=8).

Parameter*	Range (mm)	Mean ± SD (mm)	Median (mm)
LB	5.4-19.0	14.3 ± 5.0	8.9
LO	3.1-7.6	6.2 ± 2.8	5.1
H	4.4-13.1	9.2 ± 5.4	8.2
WO	1.7-6.2	4.4 ± 2.4	3.4
WB	5.0-20.0	13.6 ± 5.6	8.9
LB/LO	1.7-3.0	2.3 ± 0.0	1.7
LB/H	1.0-2.3	1.6 ± 0.1	1.1
LB/WO	2.5-3.9	3.2 ± 0.5	2.8
LB/WB	0.9-1.2	1.1 ± 0.1	1.0
LO/H	0.6-1.1	0.7 ± 0.1	0.7
LO/WO	1.2-1.8	1.5 ± 0.3	1.6
LO/WB	0.4-0.6	0.5 ± 0.1	0.6
WO/H	0.4-0.7	0.5 ± 0.0	0.4
WB/H	1.1-2.3	1.5 ± 0.0	1.1
WB/WO	2.5-3.8	3.1 ± 0.3	2.7

*LB=Basal length; LO=Orifice length; H=Carinal height; WO=Orifice width; WB=Basal width.

Supplementary Table 27. Kimura 2-parameter (K2P) distances of COI sequences between species

!Title: *Microeuraphia* sp1;
!Format DataType=Distance DataFormat=LowerLeft NTaxa=29;
!Description
Analysis ====================
Analysis = ====================
Scope = Pairs of taxa
Estimate Variance = ====================
Variance Estimation Method = Bootstrap method
No. of Bootstrap Replications = 1000
Substitution Model = ====================
Substitutions Type = Nucleotide
Model/Method = Kimura 2-parameter model
Substitutions to Include = d: Transitions + Transversions
Rates and Patterns = ====================
Rates among Sites = Uniform Rates
Pattern among Lineages = Same (Homogeneous)
Data Subset to Use = ====================
Gaps/Missing Data Treatment = Pairwise deletion
Select Codon Positions = 1st,2nd,3rd,Non-Coding
No. of Sites:641
d:Estimate
S.E:Standard error
[1] #Microeuraphia_sp1_Bcl3917PM1359_{Microeuraphia_sp1}
[2] #Microeuraphia_sp1_Bcl3917PM1358_{Microeuraphia_sp1}
[3] #Microeuraphia_sp2_JX083873.1_{Microeuraphia_sp2}
[4] #Microeuraphia_sp2_Bcl4917PM1510_{Microeuraphia_sp2}
[5] #Microeuraphia_sp2_Bcl5517PM1641_{Microeuraphia_sp2}
[6] #Microeuraphia_sp2_X4PM1494_{Microeuraphia_sp2}
[7] #Microeuraphia_sp2_Bcl6217PM1725_{Microeuraphia_sp2}
[8] #Microeuraphia_sp2_Bcl5317PM1625_{Microeuraphia_sp2}
[9] #Microeuraphia_sp2_X5PM1581_{Microeuraphia_sp2}
[10] #Chthamalus_moro_Bcl2116PM787_{Chthamalus_moro}
[11] #Chthamalus_moro_Bcl4317PM1388_{Chthamalus_moro}
[12] #Chthamalus_moro_Bcl4116PM902_{Chthamalus_moro}
[13] #Chthamalus_moro_Bcl0417PM14_{Chthamalus_moro}
[14] #Chthamalus_moro_Bcl1417PM289_{Chthamalus_moro}
[15] #Chthamalus_moro_Bcl3316PM872_{Chthamalus_moro}
[16] #Chthamalus_moro_Bcl8816PM1217_{Chthamalus_moro}
[17] #Chthamalus_moro_Bcl1217PM243_{Chthamalus_moro}
[18] #Chthamalus_moro_KJ010437.1_{Chthamalus_moro}
[19] #Chthamalus_moro_KJ010460.1_{Chthamalus_moro}
[20] #Chthamalus_moro_Bcl2717PM613_{Chthamalus_moro}
[21] #Chthamalus_moro_Bcl4317PM1389_{Chthamalus_moro}
[22] #Chthamalus_moro_Bcl5916PM1052_{Chthamalus_moro}
[23] #Chthamalus_moro_Bcl8816PM1218_{Chthamalus_moro}
[24] #Chthamalus_malayensis_EU304446.1_{Chthamalus_malayensis}
[25] #Chthamalus_malayensis_EU304427.1_{Chthamalus_malayensis}
[26] #Pseudoctomeris_sulcata_KC138504.1_{Pseudoctomeris_sulcata}
[27] #Pseudoctomeris_sulcata_KC138503.1_{Pseudoctomeris_sulcata}
[28] #Hexechamaesipho_pilbryi_KC896285.1_{Hexechamaesipho_pilsbry}
[29] #Hexechamaesipho_pilbryi_KC896196.1_{Hexechamaesipho_pilsbry}

[1 2 3 4 5 6 7 8 9 10 11 12 13 14 15 16 17 18 19
20 21 22 23 24 25 26 27 28 29
[1]

[2] 0.01741
[3] 0.11657 0.11824
[4] 0.10524 0.11066 0.10892
[5] 0.10143 0.10682 0.10321 0.00628
[6] 0.09957 0.10494 0.10135 0.00786 0.00156
[7] 0.10847 0.11391 0.11219 0.01262 0.00942 0.00942
[8] 0.11078 0.11625 0.11070 0.01262 0.00944 0.00944 0.00628
[9] 0.10506 0.11047 0.10873 0.01103 0.00785 0.00785 0.00784 0.00470
[10] 0.18576 0.18549 0.20097 0.16661 0.16669 0.16669 0.17069 0.17088 0.17088
[11] 0.17655 0.17837 0.19770 0.16001 0.16008 0.16008 0.16590 0.16606 0.16414 0.02269
[12] 0.18663 0.18845 0.19734 0.16398 0.16377 0.16377 0.16962 0.17010 0.16787 0.02436 0.01266
[13] 0.18037 0.18635 0.19521 0.16199 0.16178 0.16178 0.16761 0.16808 0.16586 0.02436 0.01106 0.00470
[14] 0.18037 0.18219 0.19734 0.16001 0.15980 0.15980 0.16561 0.16606 0.16386 0.02269 0.01266 0.01264 0.01104
[15] 0.18453 0.18635 0.19734 0.16799 0.16778 0.16778 0.17366 0.17416 0.17192 0.02269 0.00946 0.00945 0.00786 0.00945
[16] 0.18245 0.18427 0.19521 0.16199 0.16178 0.16178 0.16962 0.17010 0.16787 0.01939 0.00946 0.00945 0.00786 0.00628 0.00628
[17] 0.19313 0.19282 0.19322 0.16808 0.16787 0.16787 0.17579 0.17427 0.17203 0.01772 0.03056 0.03383 0.03217 0.02886 0.03051 0.02721
[18] 0.18419 0.18152 0.19693 0.16432 0.16648 0.16648 0.17357 0.17171 0.16902 0.00774 0.02317 0.02908 0.02709 0.02510 0.02510 0.02116 0.02112
[19] 0.17917 0.17653 0.19437 0.16192 0.16166 0.16166 0.16871 0.16927 0.16417 0.00774 0.02317 0.02709 0.02510 0.02313 0.02313 0.01920 0.01916 0.00951
[20] 0.18663 0.18635 0.19948 0.16598 0.16577 0.16577 0.17164 0.17212 0.16989 0.00638 0.01911 0.02233 0.02070 0.02233 0.01908 0.01908 0.01744 0.00951 0.00759
[21] 0.18663 0.18635 0.19734 0.16598 0.16577 0.16577 0.17164 0.17212 0.16989 0.00478 0.02074 0.02397 0.02233 0.02070 0.02070 0.01746 0.01583 0.00759 0.00568 0.00470
[22] 0.18453 0.18427 0.19734 0.16799 0.16778 0.16778 0.17366 0.17416 0.17192 0.01284 0.02565 0.02890 0.02725 0.02561 0.02561 0.02233 0.01103 0.01530 0.01336 0.01264 0.01104
[23] 0.18873 0.19056 0.20163 0.16799 0.16778 0.16778 0.17366 0.17416 0.17192 0.01122 0.02074 0.02397 0.02233 0.02070 0.02070 0.01746 0.01262 0.01530 0.01336 0.01104 0.00945 0.00786
[24] 0.16067 0.15864 0.14139 0.14065 0.14068 0.13873 0.14844 0.14653 0.14653 0.14783 0.15481 0.15481 0.15481 0.15888 0.16092 0.15481 0.15489 0.14955 0.14718 0.15078 0.14878 0.15279 0.15481
[25] 0.19383 0.19145 0.17812 0.18701 0.18498 0.18710 0.19108 0.19126 0.18701 0.17194 0.18325 0.17903 0.17903 0.17903 0.18537 0.17903 0.18953 0.17079 0.17079 0.17693 0.17693 0.17693 0.18325 0.12208
[26] 0.22366 0.22552 0.22039 0.21550 0.20647 0.20429 0.21455 0.21754 0.21511 0.21094 0.22280 0.22018 0.21798 0.22018 0.22018 0.22239 0.22478 0.21882 0.22416 0.21360 0.21798 0.21360 0.22239 0.22431 0.20447
[27] 0.22798 0.22984 0.22470 0.21310 0.20849 0.20630 0.21659 0.21958 0.21714 0.20451 0.21633 0.21375 0.21157 0.21375 0.21375 0.21593 0.21829 0.20849 0.21372 0.20724 0.21157 0.20724 0.21593 0.22220 0.20434 0.01266
[28] 0.21973 0.22442 0.20993 0.21598 0.21341 0.21109 0.22207 0.22285 0.22025 0.21070 0.21634 0.21827 0.21592 0.21358 0.22299 0.21592 0.22320 0.21187 0.20923 0.21827 0.21827 0.22062 0.22299 0.19075 0.20642 0.20163 0.19951
[29] 0.21341 0.21076 0.21460 0.22582 0.22320 0.22082 0.23194 0.23283 0.23017 0.20402 0.20738 0.21395 0.21162 0.20929 0.20929 0.21162 0.21887 0.20944 0.20680 0.20929 0.20929 0.21162 0.21395 0.18828 0.20182 0.20911 0.20698 0.05390

Estimates of Evolutionary Divergence between Sequences

The number of base substitutions per site from between sequences are shown. Standard error estimate(s) are shown above the diagonal and were obtained by a bootstrap procedure (1000 replicates). Analyses were conducted using the Kimura 2-parameter model [1]. This analysis involved 30 nucleotide sequences. Codon positions included were 1st+2nd+3rd+Noncoding. All ambiguous positions were removed for each sequence pair (pairwise deletion option). There was a total of 641 positions in the final dataset. Evolutionary analyses were conducted in MEGA X [2] The presence of n/c in the results denotes cases in which it was not possible to estimate evolutionary distances.

Supplementary Table 28. Kimura 2-parameter (K2P) distances of COI sequences between species

Title: *Amphibalanus* sp. with Balanidae_COI
Description
Analysis ====================
Analysis = ====================
Scope = Pairs of taxa
Estimate Variance = ====================
Variance Estimation Method = Bootstrap method
No. of Bootstrap Replications = 1000
Substitution Model = ====================
Substitutions Type = Nucleotide
Model/Method = Kimura 2-parameter model
Substitutions to Include = d: Transitions + Transversions
Rates and Patterns = ====================
Rates among Sites = Uniform Rates
Pattern among Lineages = Same (Homogeneous)
Data Subset to Use = ====================
Gaps/Missing Data Treatment = Pairwise deletion
Select Codon Positions = 1st,2nd,3rd,Non-Coding
No. of Sites:641
d:Estimate
S.E:Standard error
[1] #Amphibalanus_sp_Bcl4117PM1378_{Amphibalanus_sp}
[2] #Amphibalanus_sp_Bcl4117PM1379_{Amphibalanus_sp}
[3] #Amphibalanus_sp_Bcl4216PM937_{Amphibalanus_sp}
[4] #Amphibalanus_sp_Bcl5217PM1550_{Amphibalanus_sp}
[5] #Amphibalanus_sp_Bcl1117PM125_{Amphibalanus_sp}
[6] #Amphibalanus_amphitrite_Bcl6717PM1780_{Amphibalanus_amphitrite}
[7] #Amphibalanus_amphitrite_KM211494.1_{Amphibalanus_amphitrite}
[8] #Amphibalanus_amphitrite_JQ035517.1_{Amphibalanus_amphitrite}
[9] #Amphibalanus_amphitrite_Bcl6317PM1730_{Amphibalanus_amphitrite}
[10] #Amphibalanus_variegatus_X3PM922_{Amphibalanus_variegatus}
[11] #Amphibalanus_variegatus_Bcl7717PM1874_{Amphibalanus_variegatus}
[12] #Amphibalanus_variegatus_Bcl4416PM953_{Amphibalanus_variegatus}
[13] #Amphibalanus_variegatus_X2PM732_{Amphibalanus_variegatus}
[14] #Amphibalanus_variegatus_Bcl5316PM1004_{Amphibalanus_variegatus}
[15] #Amphibalanus_variegatus_KC138446.1_{Amphibalanus_variegatus}
[16] #Amphibalanus_variegatus_JQ035521.1_{Amphibalanus_variegatus}
[17] #Amphibalanus_zhujiangensis_Bcl4517PM1405_{Amphibalanus_zhujiangensis}
[18] #Amphibalanus_zhujiangensis_Bcl2316PM803_{Amphibalanus_zhujiangensis}
[19] #Amphibalanus_zhujiangensis_Bcl4517PM1407_{Amphibalanus_zhujiangensis}
[20] #Amphibalanus_zhujiangensis_KC138448.1_{Amphibalanus_zhujiangensis}
[21] #Amphibalanus_zhujiangensis_Bcl4517PM1406_{Amphibalanus_zhujiangensis}
[22] #Amphibalanus_zhujiangensis_Bcl1716PM775_{Amphibalanus_zhujiangensis}
[23] #Amphibalanus_zhujiangensis_Bcl3317PM1307_{Amphibalanus_zhujiangensis}
[24] #Amphibalanus_zhujiangensis_Bcl9016PM1222_{Amphibalanus_zhujiangensis}
[25] #Amphibalanus_zhujiangensis_Bcl4517PM1404_{Amphibalanus_zhujiangensis}
[26] #Megabalanus_tintinnabulum_KC138488.1_{Megabalanus_tintinnabulum}
[27] #Megabalanus_tintinnabulum_KC138487.1_{Megabalanus_tintinnabulum}
[28] #Megabalanus_zebra_KX538962.1_{Megabalnus_zebra}
[29] #Megabalanus_zebra_KC138491.1_{Megabalnus_zebra}
[30] #Balanus_trigonus_KU204234.1_{Balanus_trigonus}
[31] #Balanus_trigonus_KU204228.1_{Balanus_trigonus}

[1 2 3 4 5 6 7 8 9 10 11
12 13 14 15 16 17 18 19 20 21 22 23
24 25 26 27 28 29 30 31]
[1] [0.0027203239][0.0027203239][0.0027203239][0.0021470889][0.0163870832][0.0169572036
][0.0164800858][0.0164581631][0.0150734784][0.0148853128][0.0149658258][0.0149862603][0.0149658258
][0.0156201472][0.0150525082][0.0193402819][0.0194672878][0.0194878611][0.0190547212][0.0193368273
][0.0192186041][0.0194476690][0.0192186041][0.0192186041][0.0193728797][0.0197399521][0.0177318654
][0.0172050236][0.0177973206][0.0179709810]
[2] 0.0046966974 [0.0000000000][0.0000000000][0.0016551544][0.0164387962][0.0169862196
][0.0164481263][0.0164158272][0.0151328766][0.0149621674][0.0150276614][0.0150461009][0.0150276614
][0.0156716558][0.0150885071][0.0193328954][0.0194806599][0.0194821695][0.0190560821][0.0193209599
][0.0192014184][0.0194247013][0.0192014184][0.0192014184][0.0194291114][0.0197770490][0.0177400219
][0.0173374427][0.0178503279][0.0180220074]
[3] 0.0046966974 0.0000000000 [0.0000000000][0.0016551544][0.0164387962][0.0169862196
][0.0164481263][0.0164158272][0.0151328766][0.0149621674][0.0150276614][0.0150461009][0.0150276614
][0.0156716558][0.0150885071][0.0193328954][0.0194806599][0.0194821695][0.0190560821][0.0193209599
][0.0192014184][0.0194247013][0.0192014184][0.0192014184][0.0194291114][0.0197770490][0.0177400219
][0.0173374427][0.0178503279][0.0180220074]
[4] 0.0046966974 0.0000000000 0.0000000000 [0.0016551544][0.0164387962][0.0169862196
][0.0164481263][0.0164158272][0.0151328766][0.0149621674][0.0150276614][0.0150461009][0.0150276614
][0.0156716558][0.0150885071][0.0193328954][0.0194806599][0.0194821695][0.0190560821][0.0193209599
][0.0192014184][0.0194247013][0.0192014184][0.0192014184][0.0194291114][0.0197770490][0.0177400219
][0.0173374427][0.0178503279][0.0180220074]
[5] 0.0031268374 0.0015625013 0.0015625013 0.0015625013 [0.0164637380][0.0169629271
][0.0164430821][0.0164164018][0.0150978453][0.0149141680][0.0149908357][0.0150103882][0.0149908357
][0.0156408747][0.0150796726][0.0193032739][0.0194372170][0.0194505723][0.0190154763][0.0192937268
][0.0191685932][0.0193957002][0.0191685932][0.0191685932][0.0193721487][0.0197394879][0.0176753478
][0.0172907304][0.0177869289][0.0179527814]
[6] 0.1481065093 0.1499921937 0.1499921937 0.1499921937 0.1480542068 [0.0094555782
][0.0086465512][0.0078733224][0.0166872899][0.0164196824][0.0163928960][0.0165801745][0.0165508226
][0.0164086139][0.0159750893][0.0195555574][0.0197214971][0.0197999103][0.0195089180][0.0196034112
][0.0198198088][0.0197999103][0.0196034112][0.0196034112][0.0197458871][0.0201601852][0.0198404902
][0.0198949386][0.0175754589][0.0173187862]
[7] 0.1540002534 0.1539343061 0.1539343061 0.1539343061 0.1518893134 0.0476872705
[0.0040746599][0.0045095804][0.0173190035][0.0171670193][0.0170978367][0.0172487556][0.0172487556
][0.0181083253][0.0176084539][0.0205799638][0.0205903308][0.0206962059][0.0203055526][0.0204793653
][0.0206655608][0.0206962059][0.0204793653][0.0204793653][0.0204954790][0.0211430233][0.0196465331
][0.0197028965][0.0187935937][0.0186730238]
[8] 0.1506313451 0.1505695796 0.1505695796 0.1505695796 0.1486202594 0.0421422092 0.0098870053
[0.0027910836][0.0164774660][0.0163106626][0.0162759696][0.0164360045][0.0164127935][0.0170803427
][0.0166382471][0.0200469158][0.0200961733][0.0201848617][0.0198396763][0.0199964494][0.0202036648
][0.0201848617][0.0199964494][0.0199964494][0.0200061706][0.0205834516][0.0197164000][0.0192197126
][0.0180767109][0.0180859806]
[9] 0.1501760779 0.1501095597 0.1501095597 0.1501095597 0.1481640320 0.0370236881 0.0115707643
0.0047114497 [0.0164340532][0.0161942379][0.0161660278][0.0163294162][0.0163047465
][0.0165756112][0.0161373645][0.0200310676][0.0200749267][0.0201536609][0.0198916473][0.0199612211
][0.0201643822][0.0201536609][0.0199612211][0.0199612211][0.0194711483][0.0200600794][0.0194321009
][0.0190463534][0.0178133503][0.0176562801]
[10] 0.1320893711 0.1339470093 0.1339470093 0.1339470093 0.1320386100 0.1454147087 0.1526468513
0.1434116583 0.1415311940 [0.0034725341][0.0015096842][0.0000000000][0.0000000000
][0.0055528015][0.0054583057][0.0194580020][0.0196559067][0.0195424522][0.0190785481][0.0193308308
][0.0191754862][0.0193691996][0.0191754862][0.0191754862][0.0172386246][0.0174469355][0.0196200705
][0.0175034845][0.0162798411][0.0165089449]
[11] 0.1294040438 0.1312415920 0.1312415920 0.1312415920 0.1293476504 0.1424806507 0.1500426640
0.1409893246 0.1386418603 0.0078940078 [0.0030738622][0.0034565516][0.0034511954
][0.0057197866][0.0057835924][0.0192876854][0.0195525360][0.0194038630][0.0190127787][0.0191897770

][0.0190329633][0.0192205475][0.0190329633][0.0190329633][0.0174788509][0.0176988018][0.0188068238][0.0175412856][0.0162419618][0.0161106137]
[12] 0.1311865825 0.1330305084 0.1330305084 0.1330305084 0.1311365668 0.1424806507 0.1500426640 0.1409893246 0.1386418603 0.0015723283 0.0062671794 [0.0015024289][0.0015003460][0.0053290060][0.0052307562][0.0190534077][0.0192637659][0.0191486927][0.0187893214][0.0189473717][0.0187980671][0.0189911976][0.0187980671][0.0187980671][0.0173853952][0.0175926718][0.0191776279][0.0175962334][0.0163314003][0.0162034047]
[13] 0.1314111174 0.1332584523 0.1332584523 0.1332584523 0.1313609168 0.1446607380 0.1520918472 0.1431634535 0.1407997149 0.0000000000 0.0078567944 0.0015649465 [0.0000000000][0.0055424034][0.0054493397][0.0193080947][0.0195059609][0.0193907698][0.0190336370][0.0191927165][0.0190371555][0.0192282884][0.0190371555][0.0190371555][0.0172228685][0.0174361602][0.0194705398][0.0174781079][0.0161780995][0.0164093396]
[14] 0.1311865825 0.1330305084 0.1330305084 0.1330305084 0.1311365668 0.1444111551 0.1520918472 0.1429161093 0.1405575714 0.0000000000 0.0078444677 0.0015625013 0.0000000000 [0.0055345819][0.0054407380][0.0192799757][0.0194774941][0.0193636825][0.0190058227][0.0191669382][0.0190111561][0.0192022511][0.0190111561][0.0190111561][0.0171889803][0.0173995404][0.0194705398][0.0174477435][0.0161499297][0.0163799183]
[15] 0.1393695352 0.1412336481 0.1412336481 0.1412336481 0.1392994097 0.1411012193 0.1582902736 0.1468567159 0.1411012193 0.0175475102 0.0190665065 0.0158761879 0.0175195597 0.0174916982 [0.0030743056][0.0190327479][0.0192450920][0.0190979381][0.0187311393][0.0189354614][0.0187991886][0.0189802020][0.0187991886][0.0187991886][0.0175879378][0.0177665301][0.0185879866][0.0181830432][0.0165038111][0.0163001310]
[16] 0.1317522722 0.1336002405 0.1336002405 0.1336002405 0.1316918412 0.1373112915 0.1521708290 0.1411012193 0.1353955803 0.0175251242 0.0190492388 0.0158565501 0.0174972452 0.0174694547 0.0062912298 [0.0188867678][0.0191192123][0.0189650208][0.0185984057][0.0187848052][0.0186586884][0.0188550660][0.0186586884][0.0186586884][0.0175932934][0.0176645435][0.0185402687][0.0179568123][0.0161893364][0.0159697234]
[17] 0.1818428275 0.1817388880 0.1817388880 0.1817388880 0.1796854970 0.1958726603 0.2026672331 0.2009364512 0.2003390586 0.1832552970 0.1819523093 0.1798904508 0.1822763051 0.1819523093 0.1804250026 0.1764158181 [0.0040851228][0.0035857935][0.0035597855][0.0038230480][0.0039098076][0.0039071329][0.0035506240][0.0035506240][0.0195997074][0.0195510828][0.0194240002][0.0183544702][0.0196728011][0.0192437511]
[18] 0.1839089462 0.1838007466 0.1838007466 0.1838007466 0.1817388880 0.2000920946 0.2026672331 0.2009364512 0.2003390586 0.1853429827 0.1840227057 0.1819523093 0.1843509969 0.1840227057 0.1824911212 0.1784692091 0.0094491001 [0.0035707469][0.0035525551][0.0038279762][0.0039251395][0.0039225783][0.0035607254][0.0035607254][0.0192823004][0.0192125343][0.0194462417][0.0180351736][0.0195758351][0.0192164712]
[19] 0.1818428275 0.1817388880 0.1817388880 0.1817388880 0.1796854970 0.2000920946 0.2026672331 0.2009364512 0.2003390586 0.1811762919 0.1798904508 0.1778370598 0.1802101864 0.1798904508 0.1783673865 0.1743708255 0.0078617972 0.0078617972 [0.0031606720][0.0034766762][0.0035136677][0.0027265842][0.0031599672][0.0031599672][0.0194732433][0.0194144965][0.0191465966][0.0183114693][0.0196521468][0.0192756694]
[20] 0.1763182033 0.1762261216 0.1762261216 0.1762261216 0.1741894589 0.1965775720 0.1982128607 0.1966901412 0.1968084919 0.1770440370 0.1764158181 0.1743708255 0.1767293661 0.1764158181 0.1742773842 0.1703057625 0.0078865988 0.0078865988 0.0062992959 [0.0027359803][0.0028424893][0.0028259279][0.0022944165][0.0022944165][0.0191311237][0.0190851324][0.0185852402][0.0178969899][0.0195837810][0.0191799545]
[21] 0.1818428275 0.1817388880 0.1817388880 0.1817388880 0.1796854970 0.1979779265 0.2004350866 0.1988087885 0.1982159132 0.1791058956 0.1778370598 0.1757920672 0.1781525704 0.1778370598 0.1763182033 0.1723341628 0.0094491001 0.0094491001 0.0078617972 0.0047170161 [0.0021691738][0.0021744601][0.0014562994][0.0014562994][0.0193744726][0.0193340828][0.0188955785][0.0181880834][0.0194529169][0.0190603609]
[22] 0.1797852114 0.1796854970 0.1796854970 0.1796854970 0.1776405044 0.2000920946 0.2026672331 0.2009364512 0.2003390586 0.1770440370 0.1757920672 0.1737554045 0.1761033872 0.1757920672 0.1742773842 0.1703057625 0.0094491001 0.0094491001 0.0078617972 0.0047170161 0.0031299007 [0.0022126512][0.0015891449][0.0015891449][0.0193138669][0.0192624396][0.0189971992][0.0181461927][0.0193437138][0.0189529137]

[23] 0.1818428275 0.1817388880 0.1817388880 0.1817388880 0.1796854970 0.2000920946 0.2026672331 0.2009364512 0.2003390586 0.1791058956 0.1778370598 0.1757920672 0.1781525704 0.1778370598 0.1763182033 0.1723341628 0.0094491001 0.0094491001 0.0047022290 0.0047170161 0.0031299007 0.0031299007 [0.0016375586][0.0016375586][0.0194732433][0.0194144965][0.0192293861][0.0183744474][0.0196206270][0.0192451575]
[24] 0.1797852114 0.1796854970 0.1796854970 0.1796854970 0.1776405044 0.1979779265 0.2004350866 0.1988087885 0.1982159132 0.1770440370 0.1757920672 0.1737554045 0.1761033872 0.1757920672 0.1742773842 0.1703057625 0.0078617972 0.0078617972 0.0062795174 0.0031397277 0.0015625013 0.0015625013 0.0015625013 [0.0000000000][0.0193138669][0.0192624396][0.0189971992][0.0181461927][0.0193437138][0.0189529137]
[25] 0.1797852114 0.1796854970 0.1796854970 0.1796854970 0.1776405044 0.1979779265 0.2004350866 0.1988087885 0.1982159132 0.1770440370 0.1757920672 0.1737554045 0.1761033872 0.1757920672 0.1742773842 0.1703057625 0.0078617972 0.0078617972 0.0062795174 0.0031397277 0.0015625013 0.0015625013 0.0015625013 0.0000000000 [0.0193138669][0.0192624396][0.0189971992][0.0181461927][0.0193437138][0.0189529137]
[26] 0.1811443398 0.1830983642 0.1830983642 0.1830983642 0.1810106785 0.1970624698 0.1963987291 0.1993348192 0.1930840283 0.1571649812 0.1587847912 0.1586036706 0.1568898057 0.1566155964 0.1625130923 0.1624278429 0.1841793867 0.1799510317 0.1841793867 0.1799510317 0.1841793867 0.1820607395 0.1841793867 0.1820607395 0.1820607395 [0.0032700255][0.0187442918][0.0112497462][0.0203508620][0.0203120752]
[27] 0.1832363931 0.1851948033 0.1851948033 0.1851948033 0.1830983642 0.1991946692 0.2031102393 0.2058004266 0.1994807040 0.1591609919 0.1607888019 0.1605996812 0.1588818402 0.1586036706 0.1645211271 0.1624278429 0.1820607395 0.1778501883 0.1820607395 0.1778501883 0.1820607395 0.1799510317 0.1820607395 0.1799510317 0.1799510317 0.0062992959 [0.0192920869][0.0112824294][0.0201076136][0.0200796057]
[28] 0.1514179949 0.1533350821 0.1533350821 0.1533350821 0.1513310714 0.1913834748 0.1939194566 0.1943235990 0.1915982140 0.1788101577 0.1694309108 0.1754321725 0.1775198583 0.1775198583 0.1697317196 0.1698291312 0.1784738060 0.1806014687 0.1784738060 0.1727681533 0.1742454510 0.1763551587 0.1784738060 0.1763551587 0.1763551587 0.1617377892 0.1680669876 [0.0185028930][0.0202375222][0.0200911423]
[29] 0.1548007064 0.1566995534 0.1566995534 0.1566995534 0.1547154239 0.1927042520 0.1852943740 0.1844567601 0.1824911212 0.1572495808 0.1568832618 0.1586915880 0.1569740831 0.1566995534 0.1646157670 0.1625130923 0.1673404115 0.1612303558 0.1673404115 0.1612303558 0.1673404115 0.1652954189 0.1673404115 0.1652954189 0.1652954189 0.0731271784 0.0731271784 0.1615694039 [0.0202809524][0.0203104232]
[30] 0.1642434099 0.1661845656 0.1661845656 0.1661845656 0.1641561653 0.1639981665 0.1750834737 0.1689184426 0.1663727911 0.1491672134 0.1482185610 0.1500807670 0.1483832908 0.1481238174 0.1505648833 0.1486436780 0.1901570607 0.1880650075 0.1880650075 0.1866531274 0.1859816711 0.1839069793 0.1859816711 0.1839069793 0.1839069793 0.2003876052 0.1982136887 0.2017444704 0.1961974277 [0.0035837854]
[31] 0.1662759330 0.1682212283 0.1682212283 0.1682212283 0.1661845656 0.1619860914 0.1750834737 0.1709760587 0.1643361284 0.1511396026 0.1462616114 0.1481238174 0.1503440776 0.1500807670 0.1486040965 0.1466867283 0.1859816711 0.1839069793 0.1839069793 0.1824950993 0.1818408607 0.1797832446 0.1818408607 0.1797832446 0.1797832446 0.1982136887 0.1960491831 0.1995985443 0.1983666285 0.0079619517

Estimates of Evolutionary Divergence between Sequences

The number of base substitutions per site from between sequences are shown. Standard error estimate(s) are shown above the diagonal. Analyses were conducted using the Kimura 2-parameter model [1]. This analysis involved 31 nucleotide sequences. Codon positions included were 1st+2nd+3rd+Noncoding. All ambiguous positions were removed for each sequence pair (pairwise deletion option). There was a total of 641 positions in the final dataset. Evolutionary analyses were conducted in MEGA X [2]

1. Kimura M. (1980). A simple method for estimating evolutionary rate of base substitutions through comparative studies of nucleotide sequences. Journal of Molecular Evolution 16:111-120.
2. Kumar S., Stecher G., Li M., Knyaz C., and Tamura K. (2018). MEGA X: Molecular Evolutionary Genetics Analysis across computing platforms. Molecular Biology and Evolution 35:1547-1549.

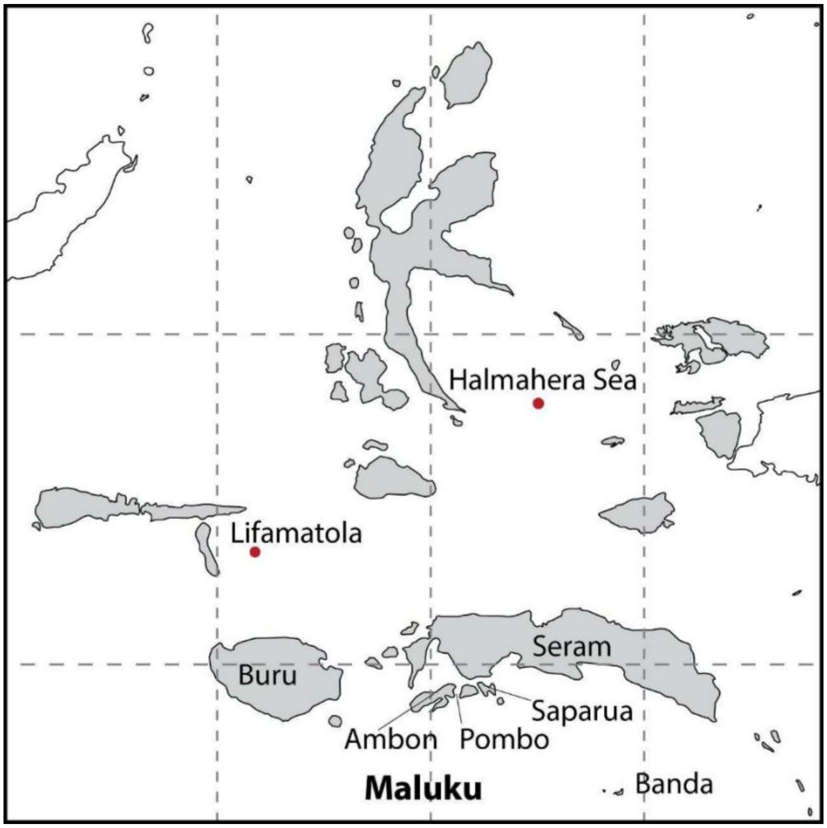

Supplementary Figure 1. The occurrence of *Heteralepas japonica* in the Moluccas (red dots).

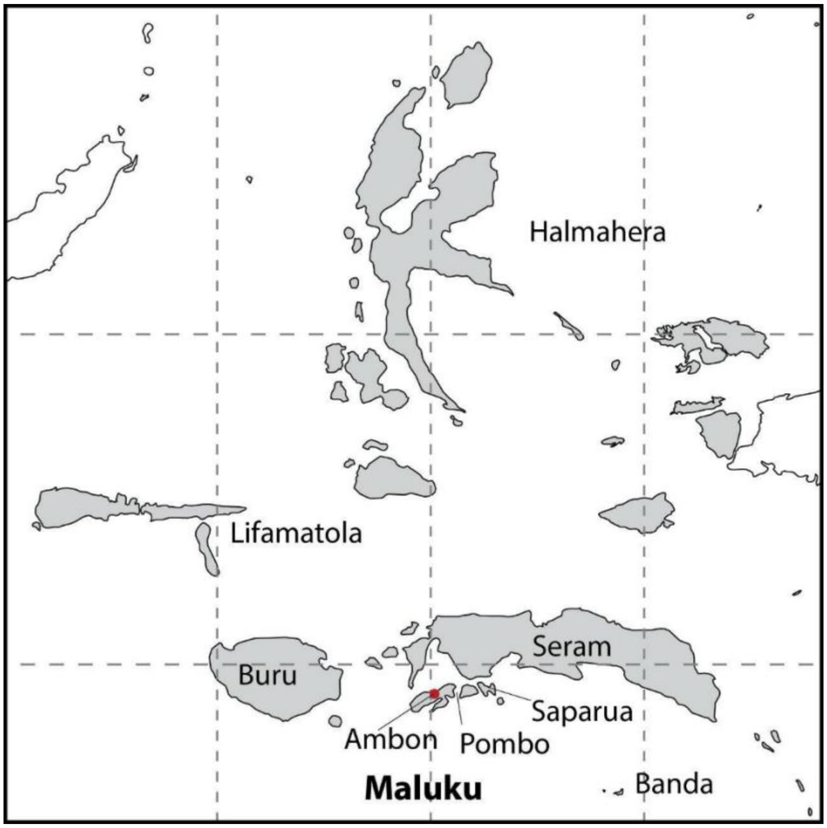

Supplementary Figure 2. The occurrence of *Amphibalanus reticulatus*, *Dosima fascicularis*, *Euraphia hembeli*, *Hexechamaesipho pilsbryi*, *Megabalanus zebra*, *Nesochthamalus intertextus*, *Newmanella spinosus*, *Pseudoctomeris sulcata*, *Tetraclitella divisa and Tetraclitella karandei* in the Moluccas (the red dot indicates that the species occurs on the coastline of the island).

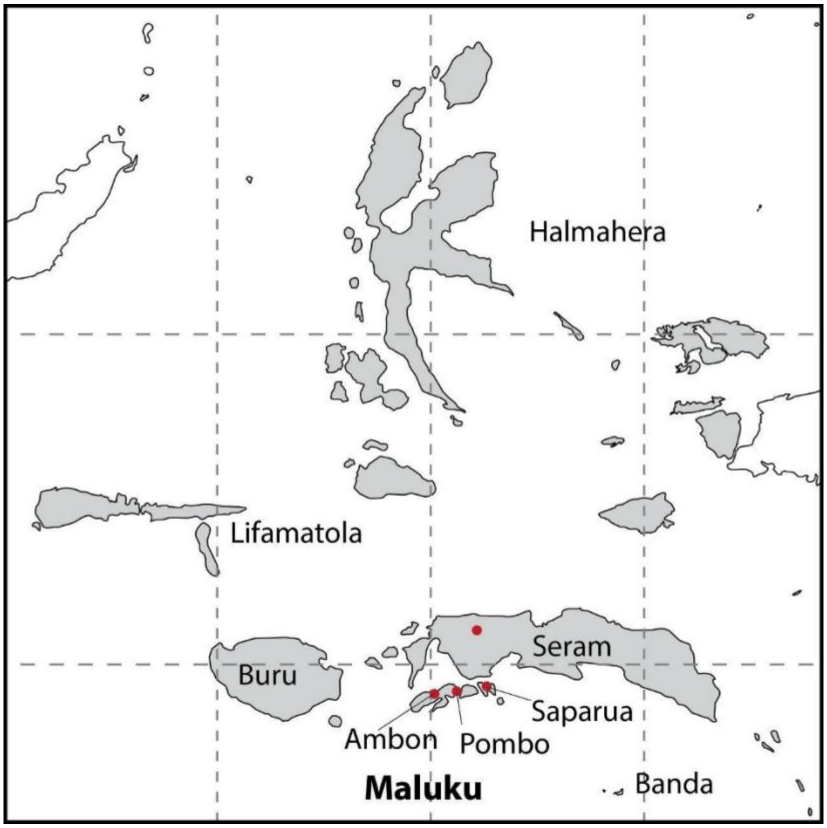

Supplementary Figure 3. The occurrence of *Chthamalus moro* and *Lepas anserifera* in the Moluccas (the red dots indicate that the species occurs on the coastline of the islands).

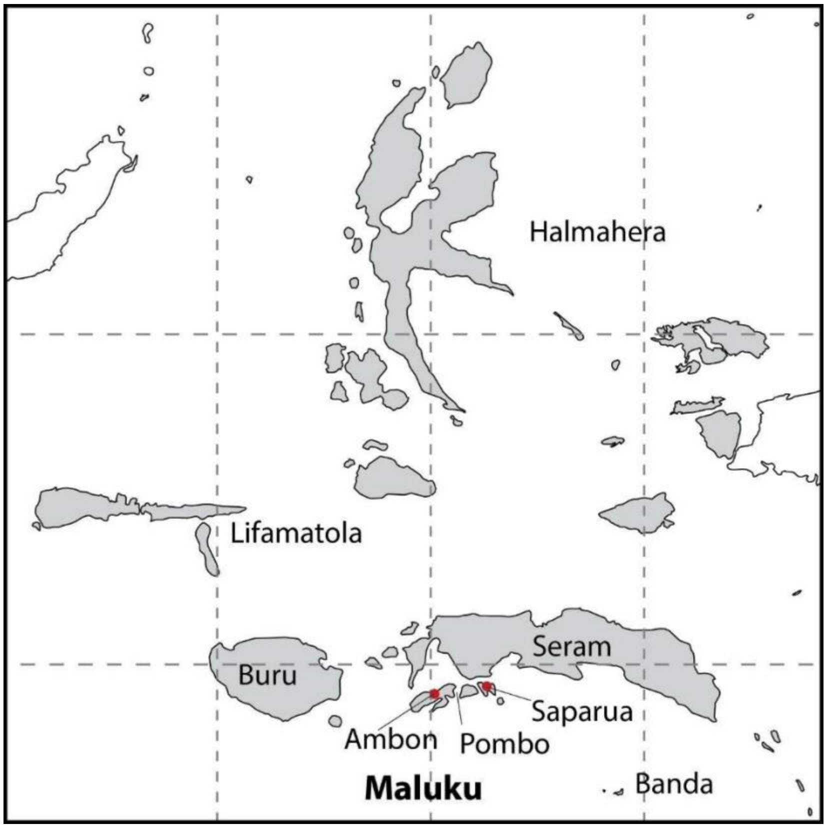

Supplementary Figure 4. The occurrence of *Amphibalanus amphitrite, Amphibalanus variegatus, Capitulum mitella, Megabalanus tintinnabulum, Tesseropora rosea, Tetraclita kuroshioensis* and *Tetraclita squamosa* in the Moluccas (the red dots indicate that the species occurs on the coastline of the islands).

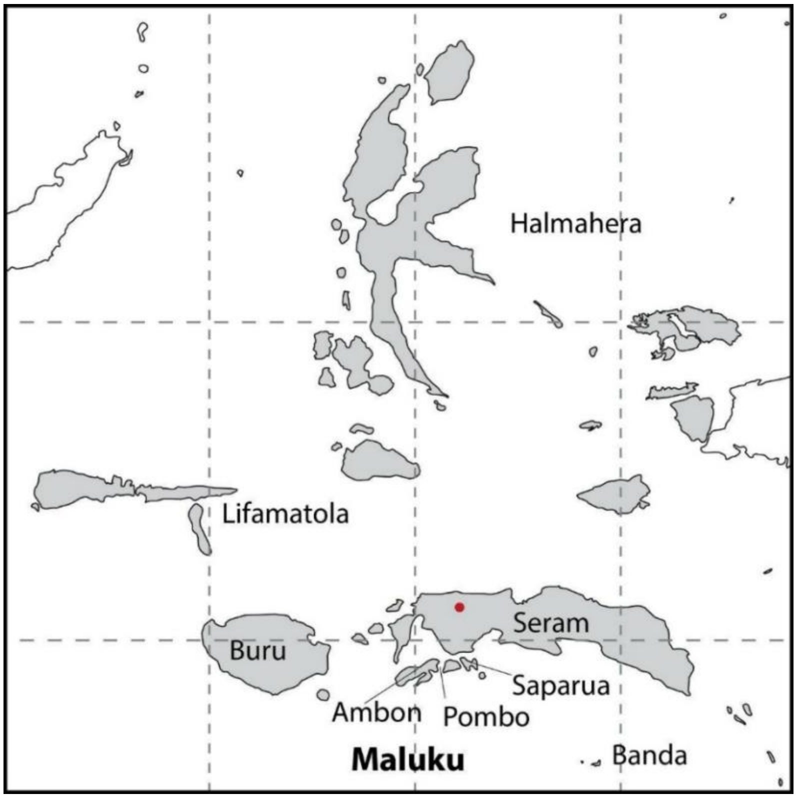

Supplementary Figure 5. The occurrence of *Microeuraphia* sp. in the Moluccas (the red dot indicates that the species occurs on the coastline of the island).

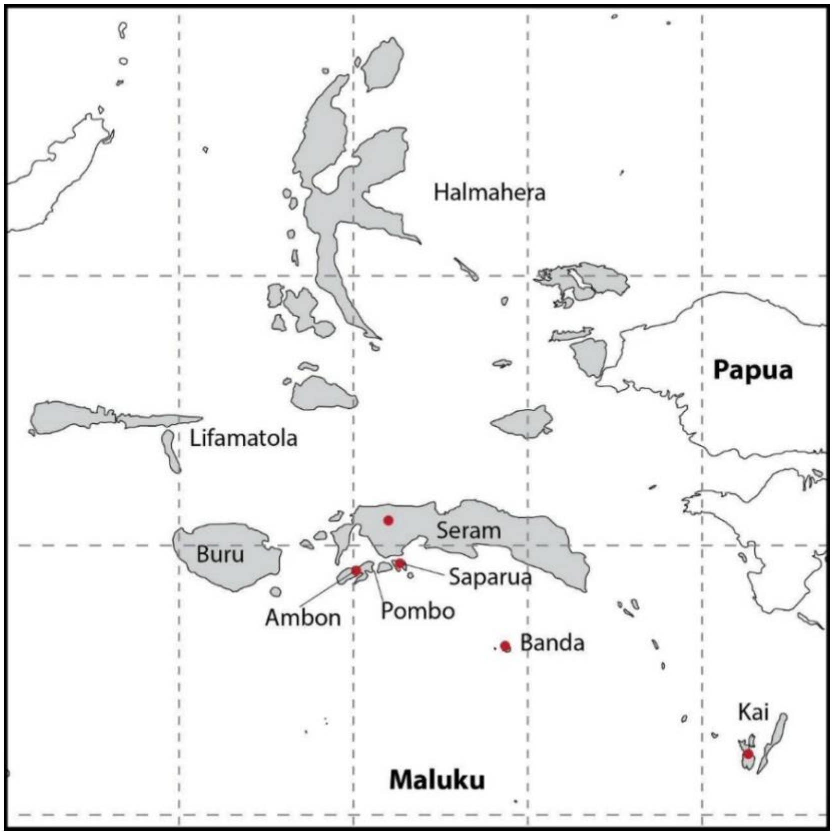

Supplementary Figure 6. The occurrence of *Yamaguchiella coerulescens* in the Moluccas (the red dots indicate that the species occurs on the coastline of the islands).

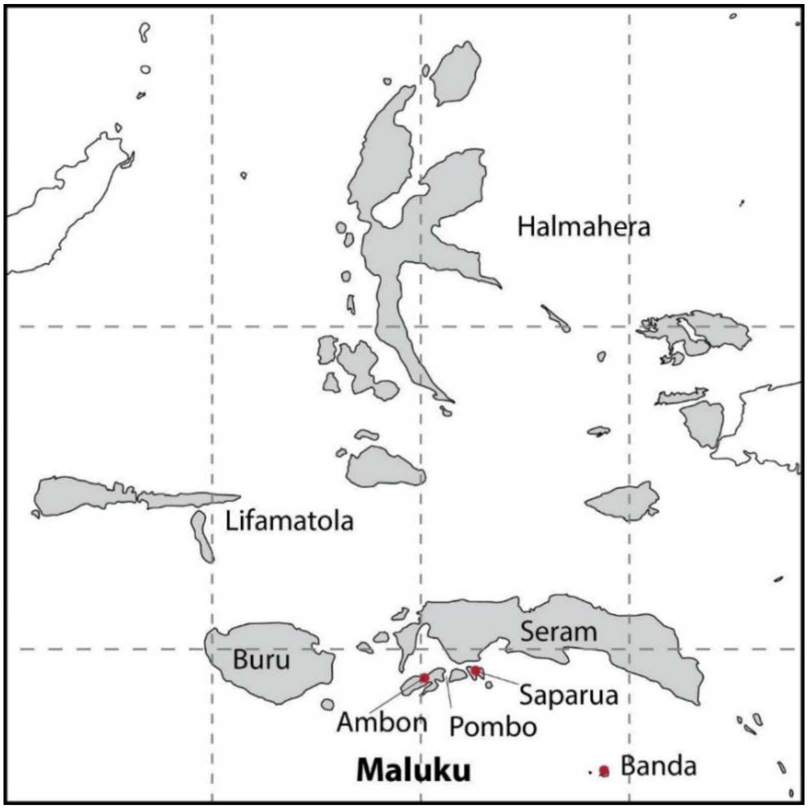

Supplementary Figure 7. The occurrence of *Neonrosella vitiata* in the Moluccas (the red dots indicate that the species occurs on the coastline of the islands).

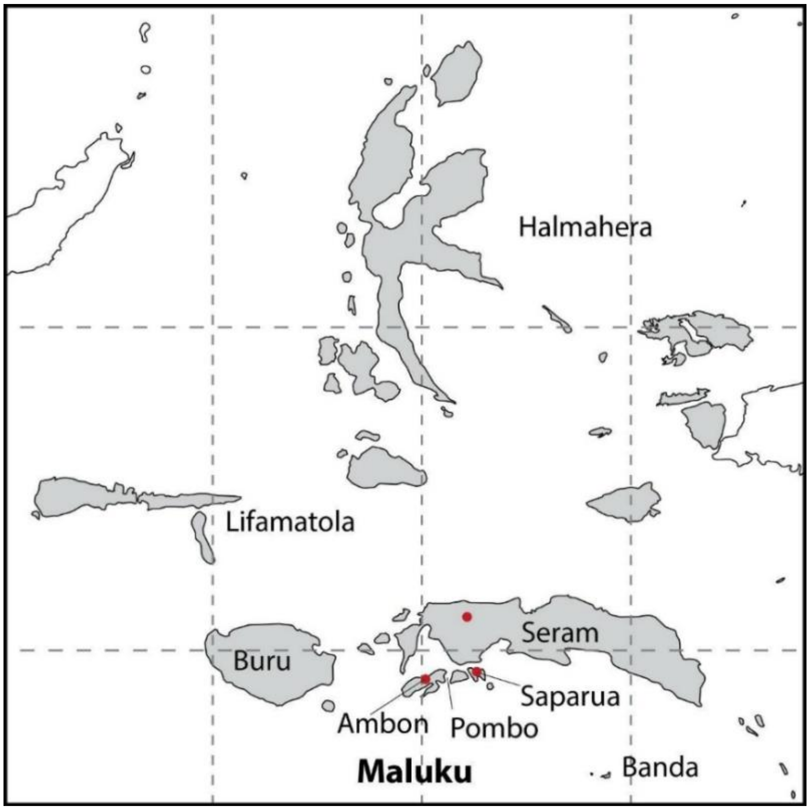

Supplementary Figure 8. The occurrence of *Amphibalanus zhujiangensis* in the Moluccas (the red dots indicate that the species occurs on the coastline of the islands).

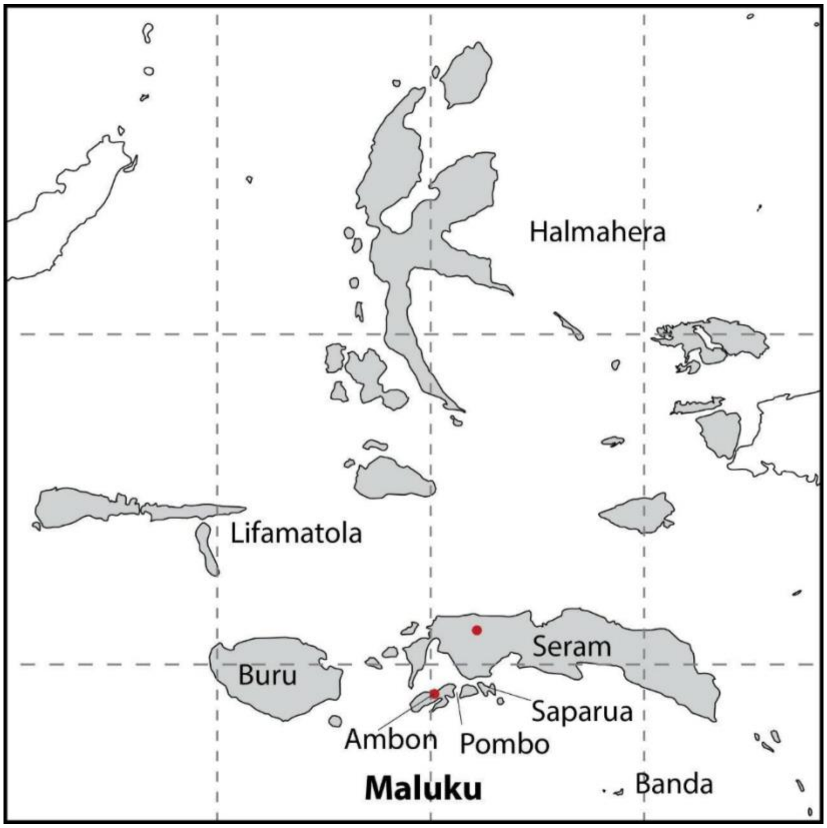

Supplementary Figure 9. The occurrence of *Amphibalanus* sp. in the Moluccas (the red dots indicate that the species occurs on the coastline of the islands).

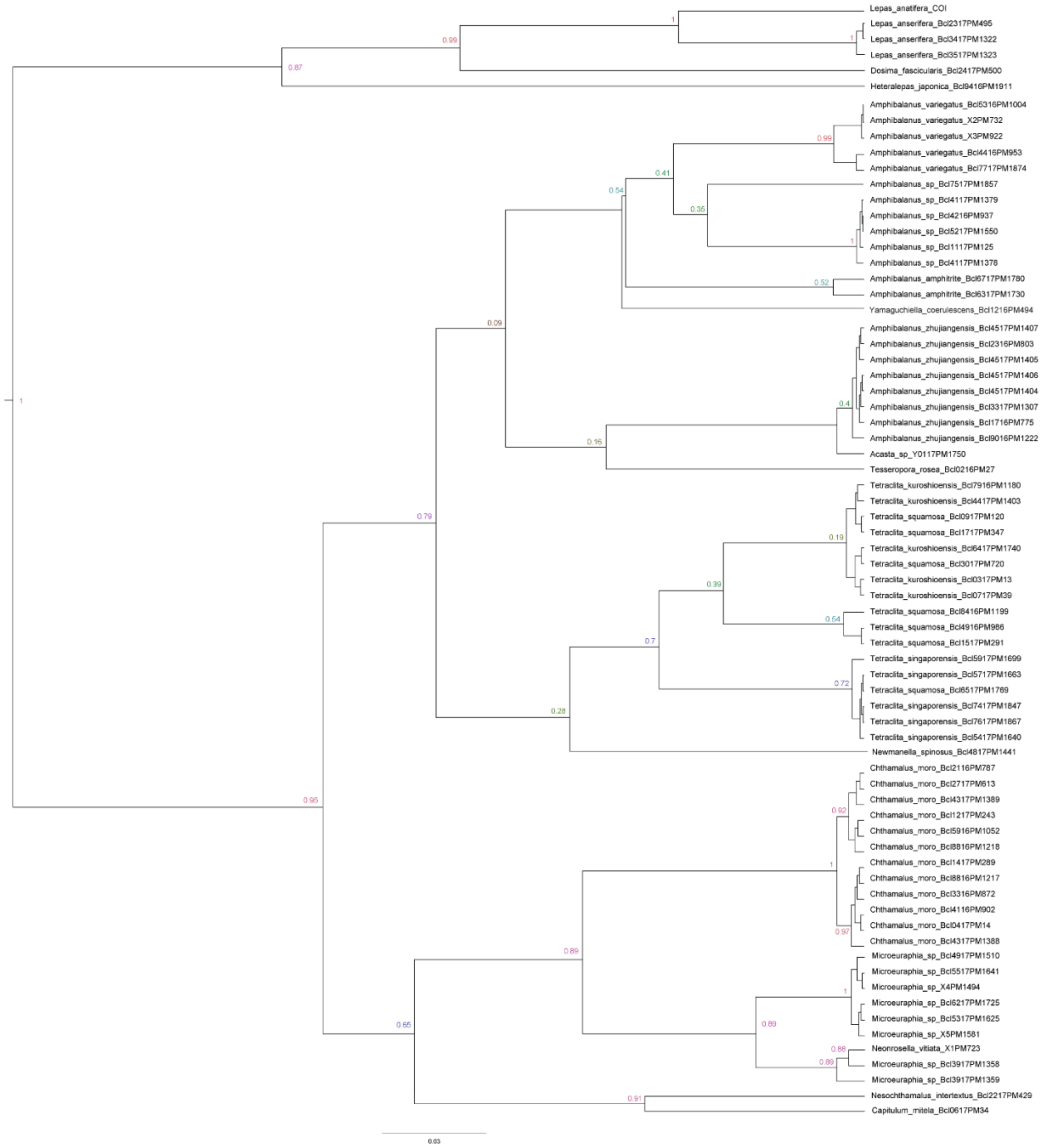

Supplementary Figure 10. Bayesian phylogeny of concatenated COI and 18S gene sequences. Values next to nodes are support values.

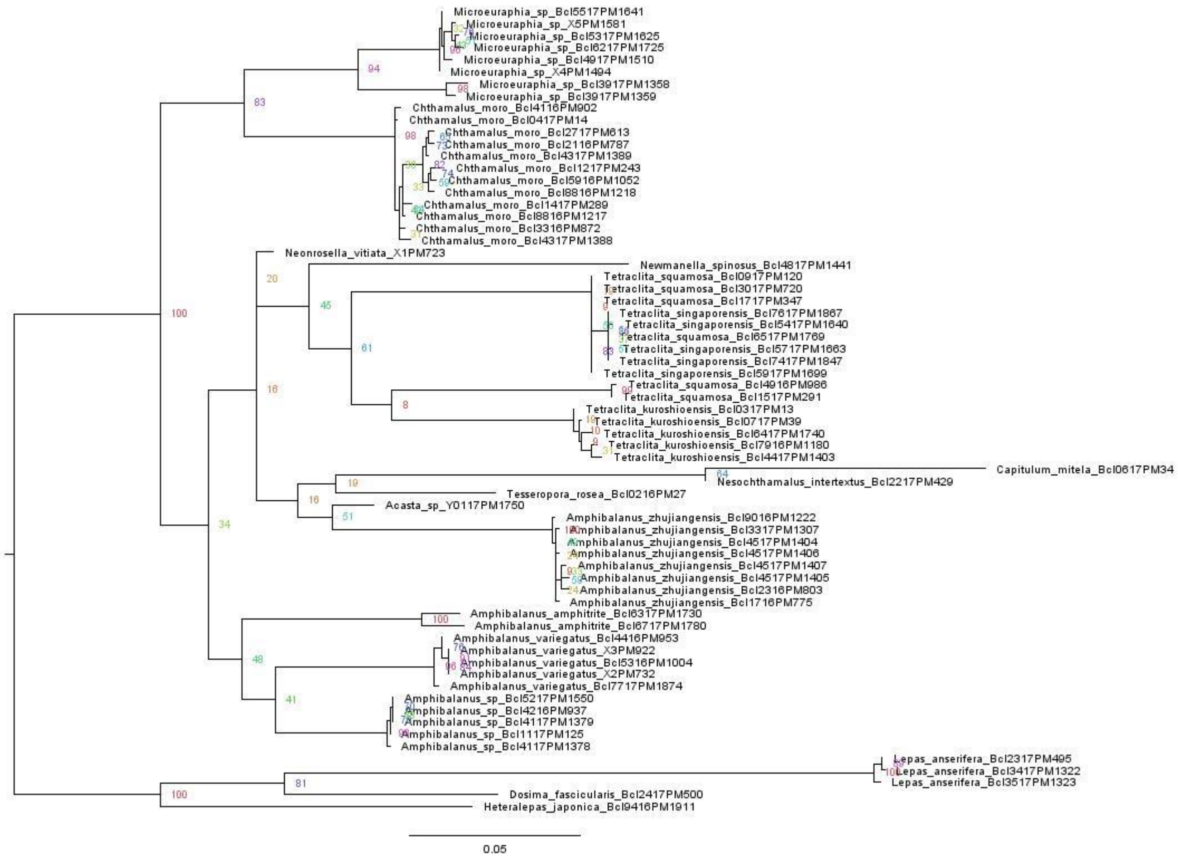

Supplementary Figure 11. ML phylogeny of concatinated COI and 18S gene sequences. Values next to nodes are support values.

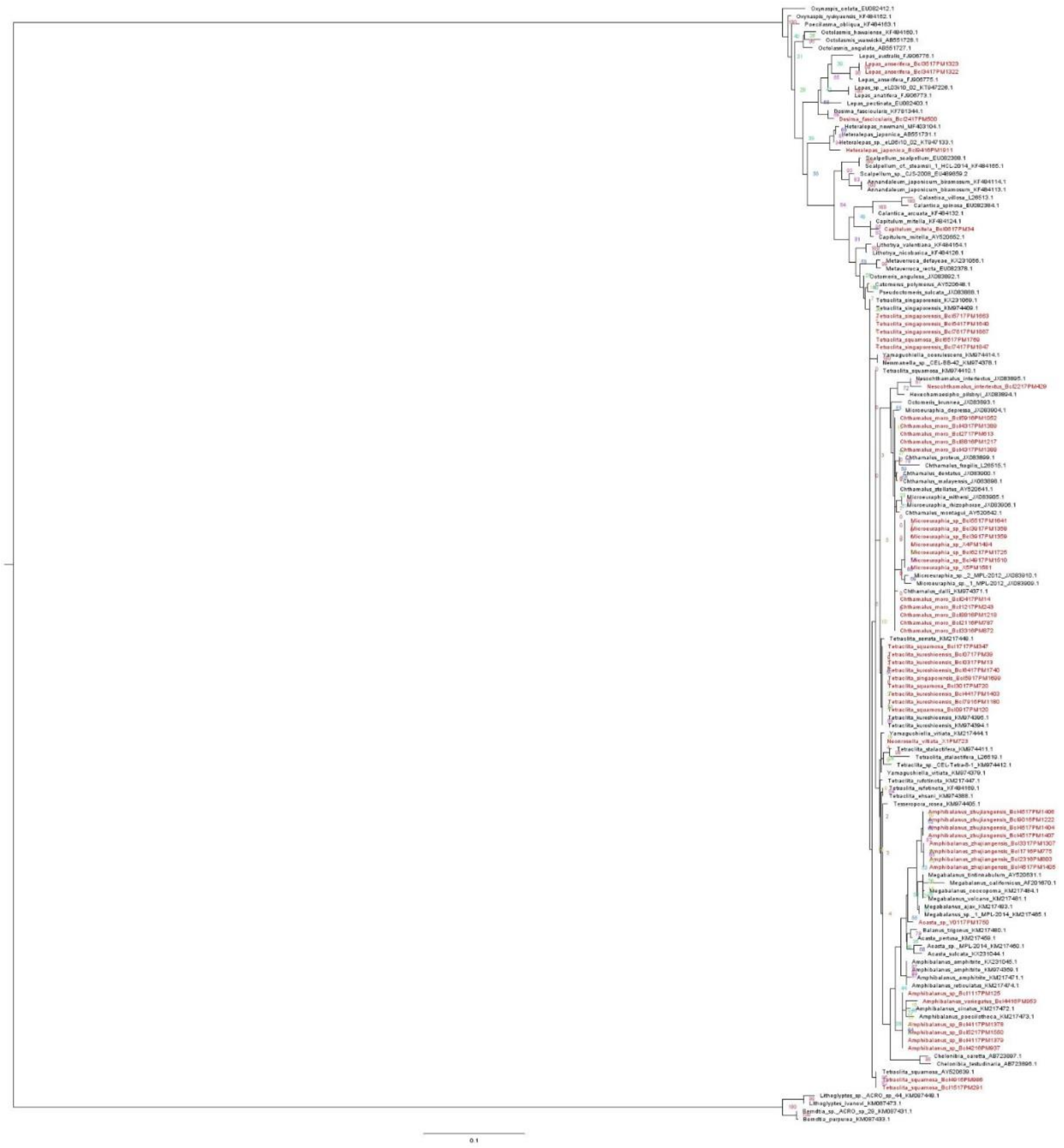

Supplementary Figure 12. ML phylogeny of 18S gene sequences. Tips coloured in red are the new samples sequenced for this manuscript. Values next to nodes are support values.

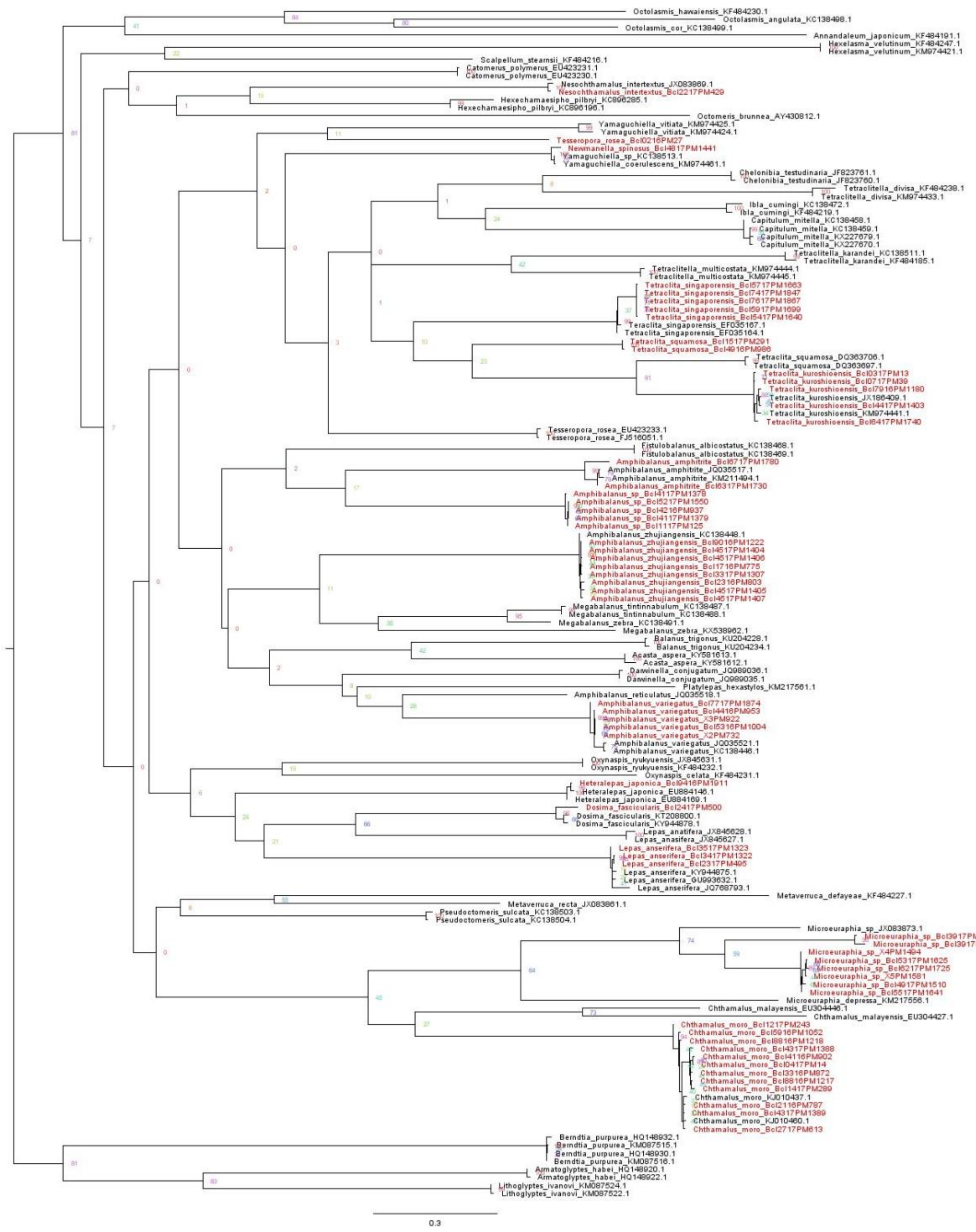

Supplementary Figure 13. ML phylogeny of COI gene sequences. Tips coloured in red are the new samples sequenced for this manuscript. Values next to nodes are support values.
